# Origin of the nuclear proteome on the basis of pre-existing nuclear localization signals in prokaryotic proteins

**DOI:** 10.1186/s13062-020-00263-6

**Published:** 2020-04-28

**Authors:** Olga M. Lisitsyna, Margarita A. Kurnaeva, Eugene A. Arifulin, Maria Y. Shubina, Yana R. Musinova, Andrey A. Mironov, Eugene V. Sheval

**Affiliations:** 1 Belozersky Institute of Physico-Chemical Biology, Lomonosov Moscow State University, 119991 Moscow, Russia; 2 Faculty of Bioengineering and Bioinformatics, Lomonosov Moscow State University, 119991 Moscow, Russia; 3 Koltzov Institute of Developmental Biology, Russian Academy of Sciences, 119334 Moscow, Russia; 4 Skobelkin State Scientific Center of Laser Medicine FMBA, 121099 Moscow, Russia; 5 Skolkovo Institute of Science and Technology, 121205, Moscow, Russia; 6 Institute for Information Transmission Problems, Russian Academy of Sciences, 127051 Moscow, Russia; 7 Faculty of Computer Science, National Research University Higher School of Economics, 101000 Moscow, Russia; 8 Department of Cell Biology and Histology, Faculty of Biology, Lomonosov Moscow State University, 119991 Moscow, Russia; 9 LIA 1066 LFR2O French-Russian Joint Cancer Research Laboratory, 94805 Villejuif, France

**Keywords:** Nucleus, Nuclear proteome, Nuclear localization signal, Evolution

## Abstract

**Background:**

The origin of the selective nuclear protein import machinery, which consists of nuclear pore complexes and adaptor molecules interacting with the nuclear localization signals (NLSs) of cargo molecules, is one of the most important events in the evolution of eukaryotic cells. How proteins were selected for import into the forming nucleus remains an open question.

**Results:**

Here, we demonstrate that functional NLSs may be integrated in the nucleotide-binding domains of both eukaryotic and prokaryotic proteins and may coevolve with these domains.

**Conclusion:**

The presence of sequences similar to NLSs in the DNA-binding domains of prokaryotic proteins might have created an advantage for nuclear accumulation of these proteins during evolution of the nuclear-cytoplasmic barrier, influencing which proteins accumulated and became compartmentalized inside the forming nucleus (i.e., the content of the nuclear proteome).

**Reviewers:**

This article was reviewed by Sergey Melnikov and Igor Rogozin.

**Open peer review:**

Reviewed by Sergey Melnikov and Igor Rogozin. For the full reviews, please go to the Reviewers’ comments section.

## Background

Acquisition of a cell nucleus enabled the spatial segregation of transcription and translation and likely permitted the evolution of more sophisticated mechanisms of gene expression regulation [1]. Because proteins are translated in the cytoplasm, the emergence of a reliable and efficient nuclear import mechanism was the essential event leading to the origin of the eukaryotic cell. Nucleocytoplasmic transport across the nuclear envelope occurs predominantly through nuclear pore complexes (NPCs). Although small proteins can freely diffuse through NPCs, globular molecules larger than ~ 40 kDa are selectively transported via an energy-dependent mechanism that requires additional transport factors, called karyopherins, which recognize nuclear localization signals (NLSs) in cargo proteins [2]. Past studies have revealed some important events in the evolution of the nuclear envelope and possible ancestors of the key elements of the import machinery: NPCs and karyopherins [3–7]. However, it remains unclear how the proteins were selected for import into the forming nucleus, i.e., how the nuclear proteome evolved.

## Methods

Human proteins containing NLSs were collected from NLSdb (https://www.rostlab.org/services/nlsdb1/browse.php/) and the UniProt database. Annotations of protein domain structure were obtained from the UniProt/Swiss-Prot database. Regions between the nearest annotated domains were analyzed as out-of-domain regions. Orthologs of human proteins with NLSs were found in the *Branchiostoma floridae*, *Danio rerio*, *Xenopus laevis*, *Pelodiscus sinensis*, and *Gallus gallus* proteomes using OrthoDB release 10 (https://www.orthodb.org/) (Supplementary Table S1).

Multiple alignment of orthologous sequences was performed with Clustal Omega. The conservation degree of multiple alignments was evaluated as the information content (IC) [8], which was calculated as follows:

1 $$ IC=\frac{\sum_j Ij}{L} $$

2 $$ Ij=\sum \limits_bI\left(b,j\right) $$

3 $$ I\;\left(b,j\right)=F\;\left(b,j\right)\times {\mathit{\log}}_2\;\frac{F\;\left(b,j\right)}{p_b} $$

4 $$ F\left(b,j\right)=\frac{N\left(b,j\right)+{p}_b}{r+1} $$

where *I*_*j*_ is the IC of the *j*^*th*^ alignment column, *L* is the length of multiple alignment, *I*_*(b,j)*_ is the *IC* of amino acid residues type "b" in the *j*^*th*^ alignment column, *F*_*(b, j)*_ is the frequency of amino acid residues type "b" in the *j*^*th*^ alignment column, *N*_*(b, j)*_ is the number of amino acid residue type 'b' in the *j*^*th*^ alignment column, *(p*_*b*_*)* (pseudo count) is the base frequency of amino acid residue type "b", and *r* is the number of rows in the alignment [9].

The *Thermococcus sibiricus* lineage was kindly provided by E.A. Bonch-Osmolovskaya*.* Genomic DNA of *Synechococcus* sp. and *Anabaena* sp. was provided by O.A. Koksharova and that of *Vibrio harveyi* by Y.V. Bertsova and A.V. Bogachev. Genes encoding target prokaryotic proteins were amplified by PCR from corresponding genomic DNA and inserted into the pEGFP-C1 vector (Clontech). Mutated genes of prokaryotic proteins were obtained by PCR site-directed mutagenesis. Double-stranded oligonucleotides encoding predicted NLSs of prokaryotic proteins were inserted into the pEGFP-C1 vector (Clontech). DNA fragments encoding M9M and Bimax2 peptides were inserted into the pTagRFP-C vector (Evrogen).

HeLa cells were grown in Dulbecco’s modified Eagle’s medium supplemented with L-glutamine (Paneco), 10% fetal calf serum (HyClone) and an antibiotic/antimycotic solution (Gibco). Cell transfection was performed using Lipofectamine 2000 reagent (Thermo Fisher Scientific) according to the manufacturer’s instructions. Images of at least 20 living HeLa cells expressing EGFP-fused proteins were acquired in two different experiments using a Nikon C2 confocal laser scanning microscope. The ratio of nucleoplasmic EGFP concentration to cytoplasmic EGFP concentration (F_nuc_/F_cyt_) was measured as described elsewhere [10]. Statistical analysis and graph preparations were performed using Prism 6 (GraphPad software).

## Results

**Fig. 1:**
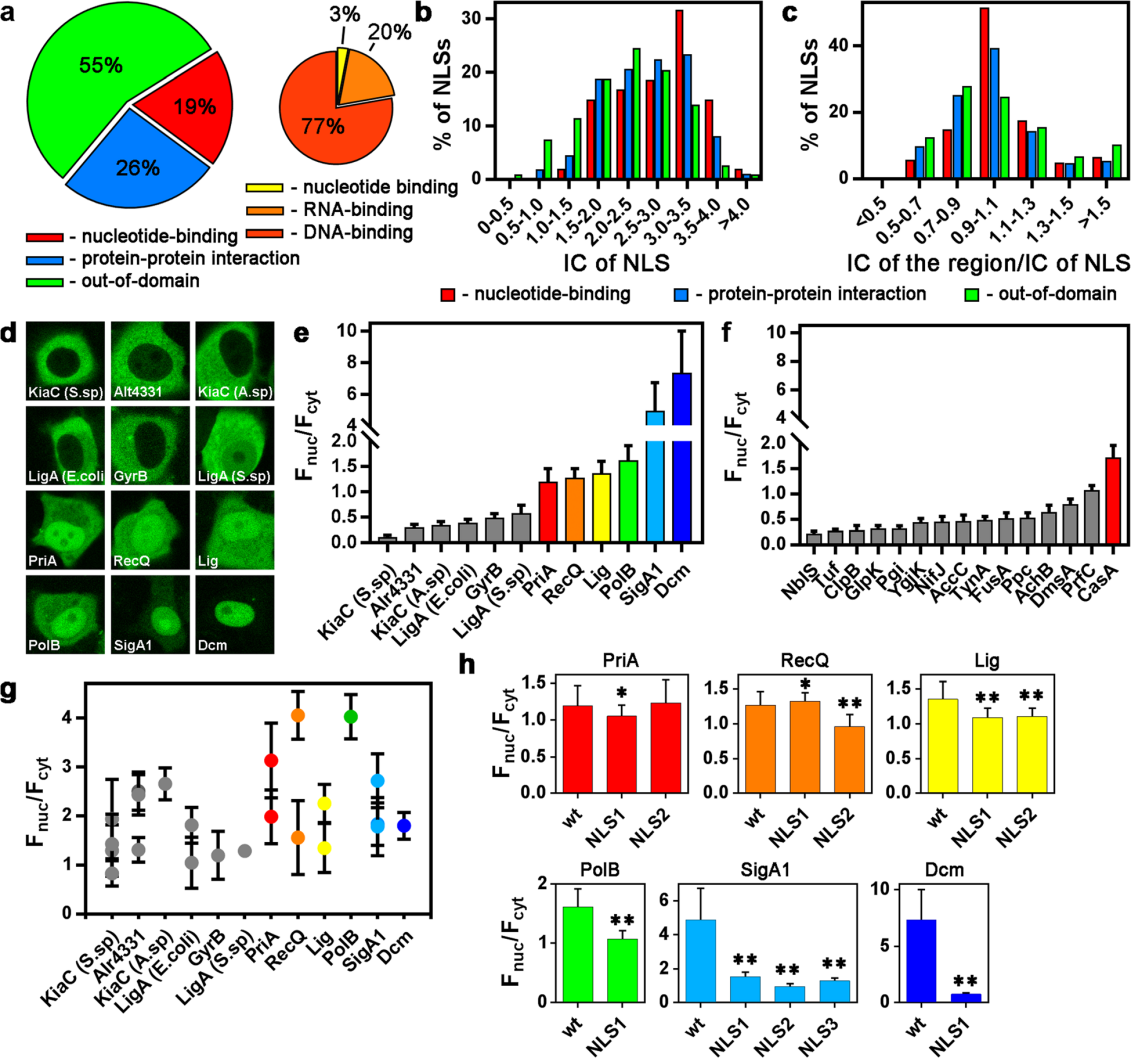
Evolutionary integration of NLSs in the annotated domains of eukaryotic and prokaryotic proteins. **a** Distribution of NLSs according to their localization in protein sequences relative to annotated protein domains. **b** IC distributions of NLSs that overlap with either nucleotide-binding domains, domains involved in protein-protein interactions or out-of-domain regions. The shift of distributions of NLSs overlapping with nucleotide-binding domains and domains involved in protein-protein interactions toward higher values of IC suggests that in-domain NLSs are more conservative relative to out-of-domain regions (one-way ANOVA test, *p* < 0.05, followed by the Bonferroni correction for multiple comparisons) **c** Distribution of the ratio of IC of the surrounding NLS region to that of the NLS. **d** Localization of prokaryotic proteins expressed as EGFP fusions in living HeLa cells. **e** Estimation of nuclear accumulation (F_nuc_/F_cyt_) of prokaryotic proteins fused to EGFP. The results are presented as the mean ± s.d. (*n* > 20). Proteins with an F_nuc_/F_cyt_ ≤ 1.16 were classified as non-accumulated inside nuclei (gray bars); those with an F_nuc_/F_cyt_ > 1.16 were classified as accumulated inside nuclei (colored bars). **f** Estimation of the nuclear accumulation of different prokaryotic proteins for which the presence of NLS(s) was not predicted using cNLS Mapper software (mean ± s.d.) (n > 20). **g** Estimation of the nuclear accumulation of EGFP fused to predicted NLSs from different prokaryotic proteins (mean ± s.d.) (n > 20). **h** Mutations in predicted NLSs influence the nuclear accumulation (F_nuc_/F_cyt_) of prokaryotic proteins. Each value represents the mean ± s.d. (n > 20). single asterisk: p < 0.05, double asterisk: *p* < 0.0001, Mann-Whitney test

To detect possible mechanisms of NLS origin, we analyzed data for NLSs localization relative to protein domains in modern organisms. We collected a dataset consisting of 592 annotated NLSs from 496 human proteins, among which 234 NLSs were identified experimentally and the other 358 NLS sequences were annotated in silico (Supplementary Table S1). Forty-five percent of all NLSs overlapped with some annotated domains (19% with nucleotide-binding domains and 26% with domains involved in protein-protein interactions); the other 55% of NLSs exhibited out-of-domain localization (Fig. 1a). The majority (77%) of the nucleotide-binding domains matching with NLSs are annotated as DNA-binding domains (Fig. 1a). Our data are in agreement with published data about colocalization of NLSs with DNA- and RNA-binding domains [11, 12].

We hypothesized the existence of an evolutionary link between NLSs and domains. To test this hypothesis, the conservation of NLSs and surrounding regions was analyzed by comparing the human protein sequences with their orthologs from five different species of phylum Chordata (*Branchiostoma floridae*, *Danio rerio*, *Xenopus laevis*, *Pelodiscus sinensis* and *Gallus gallus*). The degree of conservation of NLSs and surrounding regions (domains or out-of-domain regions) was evaluated as the IC of the obtained multiple alignment when the most conserved position in the alignment had a higher IC value. Comparison of the calculated IC distribution in three groups of NLSs demonstrated that NLSs overlapping annotated domains (both nucleotide-binding domains and domains involved in protein-protein interactions) are more conserved than NLSs located outside annotated domains (one-way ANOVA test, *p* < 0.05, followed by the Bonferroni correction for multiple comparisons) (Fig. 1b). To compare conservation between protein regions and NLSs overlapping these regions, the ratio of IC of each region to the IC of the corresponding NLS was calculated (Fig. 1c). Approximately half of all NLSs overlapping nucleotide-binding domains had the same conservation degree as the corresponding domains (for 51% of NLSs, the ratio was within the 0.9–1.1 interval). These NLSs are integrated into nucleotide-binding domains, and their evolution might depend on the evolution of the domains. Those NLSs overlapping domains involved in protein-protein interactions exhibited lower similarity with the surrounding protein regions (for 39% of NLSs, the ratio was within the 0.9–1.1 interval); NLSs located outside domains did not demonstrate substantial similarity with the surrounding regions (the ratio was within the 0.9–1.1 interval only for 25% of NLSs).

NLSs are short and structurally simple sequences. For example, a monopartite ‘classical’ NLS has a degenerate consensus sequence of K(K/R)X(K/R) [13]. As nucleotide-binding domains are enriched in positively charged amino acids, the occasional appearance of such NLSs in such domains seems probable. As similar nucleotide-binding domains may be found in prokaryotic proteins, it seems plausible that these domains already contain sequences that potentially function as NLSs. If this supposition is correct, then such prokaryotic proteins would accumulate inside nuclei after expression in eukaryotic cells. We cloned 12 large (> 45 kDa) prokaryotic proteins with nucleotide-binding domains. The NLSs of all of the these proteins were predicted using cNLS Mapper [14], and at least one overlapped with a nucleotide-binding domain (Supplementary Table S2). To produce a control group of proteins, we cloned and analyzed 15 large (> 45 kDa) proteins without predicted NLSs (Supplementary Table S2). The proteins were fused to enhanced green fluorescent protein (EGFP), and their localization was investigated in living HeLa cells. Approximately half of all the proteins accumulated inside nuclei, though to different degrees (Fig. 1d). To quantify the efficiency of nuclear accumulation in the nucleus, the ratio of nucleoplasmic to cytoplasmic (F_nuc_/F_cyt_) fluorescence was measured for all the proteins, as described elsewhere [10]. Proteins with F_nuc_/F_cyt_ values higher than that of EGFP, i.e., > 1.16, were classified as accumulating inside nuclei (Fig. 1e, Supplementary Table S2). No correlation between the efficiency of nuclear accumulation and the molecular weight of prokaryotic proteins was detected (Pearson correlation coefficient = 0.13), indicating that the transfer of proteins into the nucleus was not due to diffusion but rather due to an active process. Among control proteins, only one accumulated inside nuclei (F_nuc_/F_cyt_ = 1.71 ± 0.25), and this protein was the only one among the 15 control proteins with DNA-binding activity (Fig. 1f; Supplementary Table S2). These data are in agreement with published results indicating that NLSs are present not only in the proteins of eukaryotes but also in the proteins of prokaryotes [15–22] and bacteriophages [23].

It is possible that nuclear import of large (> 40 kDa) proteins depends on the presence of NLSs, which were predicted in all investigated prokaryotic proteins using cNLS Mapper [13] (Supplementary Table S2). To confirm that these protein regions are indeed functionally active NLSs, we constructed plasmids coding the predicted NLSs fused to EGFP. All of the predicted NLSs were able to accumulate EGFP inside nuclei (Fig. 1g, Supplementary Table S3); however, the F_nuc_/F_cyt_ values of the predicted NLSs did not correlate with the F_nuc_/F_cyt_ values of the corresponding full-length proteins (Supplementary Fig. S1) (Pearson’s correlation coefficient between the F_nuc_/F_cyt_ of the strongest among all predicted NLSs and the F_nuc_/F_cyt_ of the proteins = 0.14). Therefore, these results can be considered only an indication of potential NLS activity. We also employed site-directed mutagenesis to directly detect the presence of NLSs.

Substitutions of all positively charged amino acids in each predicted NLS with alanine decreased the nuclear accumulation (F_nuc_/F_cyt_) of all proteins that had been classified as accumulated inside nuclei (F_nuc_/F_cyt_ > 1.16) (Fig. 1h, Supplementary Table S4).

Nuclear import of proteins containing a classical NLS depends on interaction of the NLS sequence with karyopherin-*α* and karyopherin-*β*; nonclassical NLSs directly interact with karyopherin-*β* for nuclear import. We next applied the inhibitors Bimax2 [24] and M9M [25], which bind highly specifically to karyopherin-*α* and karyopherin-*β2*, respectively. Nuclear accumulation of PriA, Lig, PolB and SigA1 was decreased by coexpression of both Bimax2 (Supplementary Fig. S2) and M9M (Supplementary Fig. S3), whereas nuclear accumulation of Dcm was reduced only by Bimax2. Accordingly, these proteins accumulate inside nuclei via the ‘classical’ karyopherin-*α*/*β*-dependent pathway. Additionally, nuclear accumulation of RecQ was decreased by coexpression of M9M but not Bimax2, indicating the presence of a nonclassical NLS in this protein.

## Discussion

Overall, our data indicate that regions enriched with the positively charged amino acids of nucleotide-binding domains can indeed serve as genuine NLSs. These NLSs are integrated into domains, and their evolution might depend on the evolution of the corresponding domains. Such NLSs, even if they are present in prokaryotic proteins, can interact with karyopherins. Karyopherins have many functions in the cell and, in particular, can act as chaperones [26, 27]. The protein domains interacting with karyopherins might have evolved before the origin of the nuclear envelope, with these domains containing sequences that potentially play a role in NLSs. The presence of sequences similar to NLSs in DNA-binding domains of prokaryotic proteins might create an advantage for nuclear accumulation of these proteins during evolution of the nuclear-cytoplasmic barrier, influencing which proteins accumulated and became compartmentalized inside the forming nucleus (the content of the nuclear proteome). Proteins that did not harbor such integrated NLSs might have acquired them de novo after nuclear envelope formation, and such NLSs can be considered separate units of genome evolution. Interestingly, sequences that are similar to NLS can also be predicted and experimentally defined as being present in some cytoplasmic proteins of modern organisms. This indicates that during evolution, some proteins, albeit possibly resident inside nuclei due to the presence of an integrated NLS, were excluded from the nucleus via different mechanisms, as discussed elsewhere [28].

**Reviewers’ comments.**

**Reviewer’s report 1.**

**Sergey Melnikov.**

**Reviewer comments:**

I reviewed this manuscript in detail when it was submitted to *Molecular Biology and Evolution*. I recommended the authors to make numerous changes, and they addressed every single of my comments. I therefore have no reason to criticize this work any further. This study is important to the field as it shows that the nuclear localization signals in modern eukaryotic proteins could simply emerge from DNA−/RNA-binding domains of cellular proteins, because having a DNA- or RNA-binding domain is frequently sufficient for a protein to be recognized as a nucleus resident. This is an important finding and I encourage you to publish this work as is.

In this concise and thought-provoking manuscript, Olga Lisitsyna et al. investigate a central evolutionary enigma: the origin of the cell nucleus. The authors convincingly show that, in most instances, all that a protein needs to enter the cell nucleus is a DNA-binding domain. For instance, in their experiments with prokaryotic proteins, they show that – even in the absence of predicted NLS sequences – some DNA-binding prokaryotic proteins are actively transported into the cell nucleus (Fig. 1). This experiment, along with their analysis of NLS overlaps with DNA-binding domains in protein structures, suggests that NLSs have initially evolved from (and within) DNA-binding domains of chromatin-binding proteins – the conclusion that makes the perfect sense from the point of evolutionary contingency. Furthermore, in their supplementary data, the authors have collected a wonderful review of the experimentally identified and predicted nuclear localization signals. This information alone will be very useful for other scientists working in the field of the origin of eukaryotes and origin of the nucleus.

My only suggestion to the authors is to divide their data set of NLSs into two groups – experimentally-defined vs in silico predicted: when they describe their statistics on the % of NLSs overlap with RNA/DNA-binding domains, it seems useful to me to provide it first for the experimentally-defined NLSs (as a more reliable data), and then complement these numbers with additional data for in silico-identified NLSs.

**Author’s response:**

We thank the reviewer for the critical evaluation of our work and the positive feedback. Of course, we agree that results based only on analysis of experimentally defined NLSs should be more robust and reliable than those based on analysis of consolidated datasets (both experimentally defined and in silico-predicted NLSs). Unfortunately, the number of experimentally defined NLSs is not as large as necessary for the appropriate statistical analysis. Therefore, we used a dataset of NLSs, including both experimentally defined and in silico-predicted NLSs.

**Reviewer’s report 2.**

**Igor Rogozin.**

**Reviewer comments:**

The authors demonstrated that NLS and NLS-like motifs may be integrated inside nucleotide binding domains of both eukaryotic and prokaryotic proteins and may co-evolve with these domains. They proposed that there are NLS-like motifs inside prokaryotic proteins that may be functionally important.

The authors need to choose the theoretical framework. If the authors would like to operate within the framework of evolutionary biology, they cannot use sentences like: “We propose that the pre-existence of NLSs inside prokaryotic proteins dictated, at least partially, the nuclear proteome composition.”. Prokaryotes do not have nucleus thus they do not have NLS and those NLS-like sequences cannot “... dictated, at least partially, the nuclear proteome composition” (due to the absence of the nucleus). Those NLS-like sequences may have some functional roles, this is possible. Just an example, fragments of mobile elements (MEs) may be a part of promoter or protein coding regions. However I doubt that the “pre-existence” of MEs “dictated” regulatory pathways or functions of protein coding genes. According to Wojtek Makalowski it is something like scrap yard (Makałowski W. Genomic scrap yard: how genomes utilize all that junk. Gene. 2000, 259(1–2):61–7). I think that the authors need to use something like “prokaryotic sequences similar to NLSs or NLS like signals etc.” (if they are willing to operate within the framework of evolutionary biology). If the authors would like to operate within frameworks of alternative hypotheses, it is better to notify readers about that. Otherwise a careful correction of logic and language is required.

This structure: … However, it remains unclear how the proteins were selected for import into the forming nuclei, i.e., how the nuclear proteome evolved." The Methods section The Results section To address this question, we analysed data on NLSs and their localization relative to protein domains. .. does not look good to me. The question and attempts to answer are separated by the Methods section.

**Author’s response:**

We thank the reviewer for taking the time to review our manuscript and for providing these comments.

We substantially modified the sentence “We propose that the pre-existence of NLSs inside prokaryotic proteins dictated, at least partially, the nuclear proteome composition”. Our logic was based on the data presented as well as on some published results (references [15–23]), which indicate that the NLSs in modern eukaryotic proteins might have evolved from the DNA-binding domains of prokaryotic proteins. As a result, some DNA-binding domains are sufficient for interaction with karyopherins, and as a consequence, a protein may have had features of a nuclear protein before the origin of the cell nucleus. Of course, these features would not be useful before the origin of the nuclear envelope. Interestingly, sequences that are similar to NLSs can also be found in some domains of cytoplasmic proteins of modern organisms (Kharitonov A.V., Shubina M.Y., Nosov G.A., Mamontova A.V., Arifulin E.A., Lisitsyna O.M., Nalobin D.S., Musinova Y.R., Sheval E.V. Switching of cardiac troponin I between nuclear and cytoplasmic localization during muscle differentiation. Biochimica et Biophysica Acta – Molecular Cell Research. 2020. 1867(2):118601). We described this as follows: “The presence of sequences similar to NLSs in DNA-binding domains of prokaryotic proteins might create an advantage for nuclear accumulation of these proteins during evolution of the nuclear-cytoplasmic barrier, influencing which proteins accumulated and became compartmentalized inside the forming nucleus (the content of the nuclear proteome). Proteins that did not harbor such integrated NLSs might have acquired them de novo after nuclear envelope formation, and such NLSs can be considered separate units of genome evolution. Interestingly, sequences that are similar to NLS can also be predicted and experimentally defined as being present in some cytoplasmic proteins of modern organisms. This indicates that during evolution, some proteins, albeit possibly resident inside nuclei due to the presence of an integrated NLS, were excluded from the nucleus via different mechanisms, as discussed elsewhere [28]”.

We modified the first sentence of the “Results” section as follows: “To detect possible mechanisms of NLS origin, we analyzed data for NLSs localization relative to protein domains in modern organisms.”

Finally, it should be noted that the manuscript was edited by American Journal Experts to improve phrasing and remove grammar and writing errors.

## Supplementary information

**Additional file 1: Supplementary Table S1.** NLSs identified experimentally or predicted in silico.

**Additional file 2: Supplementary Table S2.** Prokaryotic proteins with or without predicted NLSs.

**Additional file 3: Supplementary Table S3.** Predicted NLSs from prokaryotic proteins are able to target EGFP to the cell nucleus.

**Additional file 4: Supplementary Table S4.** Detection of NLSs inside prokaryotic proteins by site-directed mutagenesis.

**Additional file 5: Supplementary Fig. S1.** Comparison of nuclear accumulation (F_n__uc_/ F_c__yt_) of all predicted NLSs and nuclear accumulation (F_nuc_ /F_cyt_) of the full-length proteins fused with EGFP in living HeLa cells. **Supplementary Fig. S2.** Decrease in nuclear accumulation of prokaryotic proteins by a peptide inhibitor of karyopherin-*α* (Bimax2). NLS from the T antigen of SV40 virus (NLS^SV40^) fused with EGFP was used as a positive control. Expression of TagRFP-Bimax2 leads to a decrease in the nuclear accumulation of NLS^SV40^. A decrease in nuclear accumulation was also detected for five prokaryotic proteins, namely, PriA, Lig, PolB, SigA1 and Dcm. **Supplementary Fig. S3.** Decrease in the nuclear accumulation of prokaryotic proteins by a peptide inhibitor of karyopherin-*β2* (M9M). The NLS from FUS protein (NLS^FUS^) fused with EGFP was used as a positive control. The expression of TagRFP-M9M leads to a decrease in the nuclear accumulation of NLS^FUS^. A decrease in nuclear accumulation was also detected for five prokaryotic proteins, namely, PriA, RecQ, Lig, PolB and SigA1.

## Data Availability

All supporting data are submitted in Supplementary Materials.
