# Supplementary material for: Origin of the nuclear proteome on the basis of pre-existing nuclear localization signals in prokaryotic proteins
Source: Biol Direct. 2020 Apr 28;15:9. doi: 10.1186/s13062-020-00263-6 (PMC7189692; doi:10.1186/s13062-020-00263-6)
Supplement: Supplementary file 1 — Additional file 1: Supplementary Table S1. NLSs identified experimentally or predicted in silico. [file 13062_2020_263_MOESM1_ESM.pdf]

Supplementary Table S1. NLSs identified experimentally or predicted *in silico*.

NLSs identified experimentally

№ of NLS	NLS sequence	№ of protein	Uniprot ID	Protein name	Protein function	Ortholog from *Danio rerio* (Uniprot/Uniprac ID)	Ortholog from *Branchiostoma floridae* (Uniprot/Uniprac ID)	Ortholog from *Xenopus leavis* (Uniprot/Uniprac ID)	Ortholog from *Pelodiscus sinesis* (Uniprot/Uniprac ID)	Ortholog from *Gallus gallus* (Uniprot/Uniprac ID)
1	KKRK LE	1	P02545	Prelamin-A/C	Nuclear assembly, chromatin organization, nuclear membrane and telomere dynamics	Q90XD7	C3ZXL7	P11048	K7G3U5	P13648
2	RRKLKN RV	2	P17861	X-box-binding protein 1	Transcription factor, regulates the unfolded protein response.	Q90X27	C3XVS7	Q7ZYC2	-	F1NAC1
3	RKKR	3	Q9BZS1	Forkhead box protein P3	Crucial for the development and inhibitory function of regulatory T-cells	A8WFW7	C3XQK2	Q5W1J5	K7FP67	Q58NQ4
4	KSGKAPRR	4	O43524	Forkhead box protein O3	Transcriptional activator which triggers apoptosis in the absence of survival factors, including neuronal cell death upon oxidative stress	Q5I0F7	C3YI59	Q6EUW1	K7F1W1	F1NNE8
5	QSSNFGPMKG GNFGGRSSGP YGGGGQYFAK PRNQGGY	5	P09651	Heterogeneous nuclear ribonucleoprotein A1	Involved in the packaging of pre-mRNA into hnRNP particles, transport of poly(A) mRNA from the nucleus to the cytoplasm and may modulate splice site selection	Q803K3	-	Q7ZX33	-	A0A1D5P5G1
6	KKRK	6	Q13620	Cullin-4B	Core component of multiple cullin-RING-based E3 ubiquitin-protein ligase complexes which mediate the ubiquitination and subsequent proteasomal degradation of target proteins	F8W428	C3ZF52	Q498E8	K7GFE2	E1BQK9
7	KRK	7	Q15911	Zinc finger homeobox protein 3	Transcriptional regulator which can act as an activator or a repressor	A0A0R4ISP1	C3ZBH5	A0A1L8GKV0	-	F1N9R6
8	NYK RPMDGTYGPP AKRHEGE	8	O14497	AT-rich interactive domain-containing protein 1A	Involved in transcriptional activation and repression of select genes by chromatin remodeling (alteration of DNA-nucleosome topology)	A0A0R4IVD0	C3ZM58	-	K7GH06	A0A1D5NZ44
9	KRK	9	O95644	Nuclear factor of activated T-cells, cytoplasmic 1	Plays a role in the inducible expression of cytokine genes in T-cells, especially in the induction of the IL-2 [illegible]	Q19AW4	-	A0A1L8FSH1	-	A0A1D5PI24
10	KRKR									
11	KVPWLKPGRSPLPSHARSQPG LCNMYK	10	P02686	Myelin basic protein	The most abundant protein component of the myelin membrane in the CNS. Protein has a role in both its formation and stabilization	E7EYA2	-	A0A1L8FY91	-	A0A1D5NWU0
12	RRVPQRKEVS RCRKCRK	11	Q9NUL5	Repressor of yield of DENV protein	Exhibits antiviral activity against dengue virus (DENV) and can inhibit the replication of all DENV serotypes	A7YY07	-	A0A1L8H2R0	-	-
13	RKKSIPLSI KNLKRKHKRK KNKITR	12	Q9C0C9	E3-independent) E2 ubiquitin-conjugating enzyme	E2/E3 hybrid ubiquitin-protein ligase that displays both E2 and E3 ligase activities and mediates monoubiquitination of target proteins	A0A0G2KHN8	C3Z6Y3	A0A1L8EMX4	-	F1P5I5
14	KKQKKQHEG SKKK	13	P49591	Serine-tRNA ligase, cytoplasmic	Catalyzes the attachment of serine to tRNA(Ser) in a two-step reaction: serine is first activated by ATP to form Ser-AMP and then transferred to the acceptor end of tRNA(Ser)	Q6DRC0	C3YPI3	Q7ZY49	K7FUL9	Q5ZM75
15	RKRSPKDK KEKDLDGAGK RRKT	14	Q7RTP6	[F-actin]-monooxygenase MICAL3	Monooxygenase that promotes depolymerization of F-actin by mediating oxidation of specific methionine residues on actin to form methionine-sulfoxide, resulting in actin filament disassembly and preventing repolymerization	E7F9T0	C3Z6G9	-	-	Q5F3F3
16	PKVRRRI	15	Q3KP66	Innate immunity activator protein	Expressed in peripheral macrophages and intestinal myeloid-derived cells, is required for optimal PRR (pattern recognition receptor)-induced signaling, [illegible]	F1QAI0	C3Z2X7	-	K7FHM9	A0A1L1RYD9
17	PYEKPRK									
18	RRRPTH									
19	KRAR	16	O15381	Nuclear valosin-containing protein-like	Participates in the assembly of the telomerase holoenzyme and effecting of telomerase activity via its [illegible]	Q803I9	C3XWH4	Q7ZXI4	K7FKS9	A0A1L1RMM8
20	RK GKLKNKGSKR KK									
21	KRANS NLVAAYEKAK KK	17	P23508	Colorectal mutant cancer protein	Suppresses cell proliferation and the Wnt/b-catenin pathway in colorectal cancer cells. Inhibits DNA binding of b-catenin/TCF/LEF transcription factors. Involved in cell migration independently of RAC1, CDC42 and p21-activated kinase (PAK) activation	A0A075BWY1	C3YKE8	-	-	F1NS46
22	LSKKR R	18	P22314	Ubiquitin-like modifier-activating enzyme 1	Catalyzes the first step in ubiquitin conjugation to mark cellular proteins for degradation through the ubiquitin-proteasome system	Q6P9P3	C3ZZI8	Q6P285	K7F4G1	F1NPI6

23	RP LQSEGAAALV TKGCQRLAAQ GARPEAPKRK WAEDGGDAPS PSKR	19	Q96KS0	Egl nine homolog 2	Cellular oxygen sensor that catalyzes, under normoxic conditions, the post-translational formation of 4-hydroxyproline in hypoxia-inducible factor (HIF) alpha proteins	E7F8S1	C3YH16	A0A1L8F3X2	K7GGI0	E1C8C0
24	RKRRKR	20	Q6X4W1	NMDA receptor synaptonuclear signaling and neuronal migration factor	Couples NMDA-sensitive glutamate receptor signaling to the nucleus and triggers long-lasting changes in the cytoarchitecture of dendrites and spine synapse processes	B8PYG1	C3ZTG8	A0A1L8F0Y0	A0A1V4J7D7	-
25	NKRKR K	21	Q9HAJ7	Histone deacetylase complex subunit SAP30L	unctions as transcription repressor	Q6NYV5	C3YZU5	A0A1L8HU31	-	R4GLF5
26	PP ARRRR	22	Q8NAG6	Ankyrin repeat and LEM domain-containing protein 1	Endonuclease that probably plays a role in the DNA damage response and DNA repair.	Q6NUU1	C3YQ02	A0A1L8HWS5	-	-
27	KKPKW DDFKKKKK	23	Q15397	Pumilio homolog 3	Inhibits the poly(ADP-ribosyl)ation activity of PARP1 and the degradation of PARP1 by CASP3 following genotoxic stress. Binds to double-stranded RNA or DNA without sequence specificity	X1WGX5	C3YCI8	Q6DFI0	K7GDL9	E1C1R5
28	RKRK	24	Q6NTE8	MRN complex-interacting protein	Plays a role in the cellular response to DNA damage and the maintenance of genome stability through its association with the MRN damage-sensing complex	Q32PP1	-	-	-	-
29	PRRRVQR KR	25	Q5TAQ9	DDB1- and CUL4-associated factor 8	May function as a substrate receptor for CUL4-DDB1 E3 ubiquitin-protein ligase complex	F1QF18	C3XZJ2	Q6NRH1	K7FA09|	A0A1D5PZY7
30	KRK	26	Q5T5X7	BEN domain-containing protein 3	Transcriptional repressor which associates with the NoRC (nucleolar remodeling complex) complex and plays a key role in repressing rDNA transcription	B7ZD94	-	A0A1L8G991	-	F1NMS3
31	RKVKKLR	27	Q86TI2	Dipeptidyl peptidase 9	Dipeptidyl peptidase that cleaves off N-terminal dipeptides from proteins having a Pro or Ala residue at position 2	B0V1L8	C3YDM8	Q6GR22	K7FG08	A0A1D5P419
32	KKR K	28	Q5T3J3	Ligand-dependent nuclear receptor-interacting factor 1	Together with SMCHD1, involved in chromosome X inactivation in females by promoting the compaction of	E7F853	-	A0A1L8HBT1	K7G507	-
33	KKRK									
34	NF QDDPHAGL	29	O43257	Zinc finger HIT domain-containing protein 1	Seems to play a role in p53-mediated apoptosis induction. Binds to NR1D2 and relieves it of its inhibitory effect on the transcription of APOC3 without affecting its DNA-binding activity	Q561S5	-	A0A1L8H473	-	-
35	KRGRKREDK ED	30	Q9NVM9	Integrator complex subunit 13	Crucial regulator of the mitotic cell cycle and development. At prophase, required for dynein anchoring to the nuclear envelope important for proper centrosome-nucleus coupling. At G2/M phase, may be required for proper spindle formation and execution of cytokinesis. Probable component of the Integrator (INT) complex, a complex involved in the small nuclear RNAs (snRNA) U1 and U2 transcription and in their 3'-box-dependent processing	Q7ZVZ8	C3YV85	Q6GLY5	K7GIR3	F1P1V8
36	KKPRKCDLTPFLVLKARKK	31	Q9NR20	Dual specificity tyrosine-phosphorylation-regulated kinase 4	Possible non-essential role in spermiogenesis	A0A0R4ITS5	C3YLM7	Q5PPW5	K7F6T0	Q5ZIU3
37	KKE IARQMKAYEK QRGTRREEQK E	32	Q9Y314	Nitric oxide synthase-interacting protein	E3 ubiquitin-protein ligase that is essential for proper development of the forebrain, the eye, and the face. Negatively regulates nitric oxide production by inducing NOS1 and NOS3 translocation to actin cytoskeleton and inhibiting their enzymatic activity	B2GNS3	C3Y5M1	A0A1L8FGZ1	-	-
38	RPKGKTLQ KRKPK	33	Q6ZN17	Protein lin-28 homolog B	Suppressor of microRNA (miRNA) biogenesis, including that of let-7 and possibly of miR107, miR-143 and miR-200c. Binds primary let-7 transcripts (pri-let-7), including pri-let-7g and pri-let-7a-1, and sequester them in the nucleolus, away from the microprocessor complex, hence preventing their processing into mature miRNA	S0BDM9	-	A0A1L8G938	K7F3U2	A0A1D5PHV1
39	RKR QR	34	O75937	DnaJ homolog subfamily C member 8	Suppresses polyglutamine (polyQ) aggregation of ATXN3 in neuronal cells	Q1ED27	C3XW14	A0A1L8HE93	K7GI79	F1ND15
40	KRER									
41	R HRKIYTKKHH	35	Q8NFU5	Inositol polyphosphate multikinase	Inositol phosphate kinase with a broad substrate specificity. Has a preference for inositol 1,4,5-trisphosphate (Ins(1,4,5)P3) and inositol 1,3,4,6-tetrakisphosphate (Ins(1,3,4,6)P4)	Q6PBN6	C3Z3L6	Q6GQ51	K7G124	F1NFX3

42	KRRRCA	36	Q53H80	Akirin-2	Required for the innate immune response. Downstream effector of the Toll-like receptor (TLR), TNF and IL-1 beta signaling pathways leading to the production of IL-6	A5WVY2	C3YH63	Q6GQB5	-	E1C249
43	KK	37	Q9C0C6	CLOCK-interacting pacemaker	Transcriptional repressor which may act as a negative-feedback regulator of CLOCK-ARNTL/BMAL1 transcriptional activity in the circadian-clock mechanism	E7FAN7	-	A0A1L8F9G1	-	F1NAE8
44	RRRQKQKGGA SRRR	38	Q9BWC9	Coiled-coil domain-containing protein 106	Promotes the degradation of p53/TP53 protein and inhibits its transactivity	Q5EB04	-	-	-	-
45	PKKRRKKK	39	Q12796	Proline-rich nuclear receptor coactivator 1	Nuclear receptor coactivator. May play a role in signal transduction	Q5TZ15	-	-	-	Q5ZII5
46	SRERK	40	O75467	Zinc finger protein 324A	May be involved in transcriptional regulation. May be involved in regulation of cell proliferation	A2RUZ2	C3XSR4	A0A1L8HBB8	K7FDC2	A0A1D5PJZ9
47	PLLKKIKQ	41	Q708E1	C-myb v-myb myeloblastosis viral oncogene homologue (avian), exon 1 and joined CDS	DNA binding	F1QP24	C3ZI75	Q05935	K7FZH8	P01103
48	KRPMNAFMVWAQAARRK	42	P48436	Transcription factor SOX-9	Transcriptional regulator that plays a role in chondrocytes differentiation and skeletal development	Q9DFH2	C3Z730	B7ZR65	K7G5M4	P48434
49	PRRRK									
50	PPQKKIKS	43	Q9UMQ5	N-myc protein	DNA-binding transcription factor activity	Q6PBB3	-	-	-	-
51	PAAKRVKLD	44	P01106	Myc proto-oncogene protein	Transcription factor that binds DNA in a non-specific manner, yet also specifically recognizes the core sequence 5'-CAC[GA]TG-3'. Activates the transcription of growth-related genes. Binds to the VEGFA promoter, promoting VEGFA production and subsequent sprouting angiogenesis	B2GQB7	C3Y235	Q2TAU1	K7GG15	F1NXY9
52	QRKRQK	45	P19838	Nuclear factor NF-kappa-B p105 subunit	NF-kappa-B is a pleiotropic transcription factor present in almost all cell types and is the endpoint of a series of signal transduction events that are initiated by a vast array of stimuli related to many biological processes such as inflammation, immunity, differentiation, cell growth, tumorigenesis and apoptosis	A0A0R4IN79	C3XR39	A0A1L8F873	K7FNJ5	Q04861
53	RRSMKRK	46	P11473	Vitamin D3 receptor	Nuclear receptor for calcitriol, the active form of vitamin D3 which mediates the action of this vitamin on cells. Enters the nucleus upon vitamin D3 binding where it forms heterodimers with the retinoid X receptor/RXR. The VDR-RXR heterodimers bind to specific response elements on DNA and activate the transcription of vitamin D3-responsive target genes. Recruited to promoters via its interaction with BAZ1B/WSTF which mediates the interaction with acetylated histones, an essential step for VDR-promoter association. Plays a central role in calcium homeostasis	Q1L673	-	B7ZPA5	-	A0A1D5NUY7
54	SKRVAKRKL	47	P10827	Thyroid hormone receptor alpha	Nuclear hormone receptor that can act as a repressor or activator of transcription. High affinity receptor for thyroid hormones, including triiodothyronine and thyroxine	A0A286Y807	C3YYX7	A0A1L8EKN8	K7FLZ6	A0A1D5PCG7
55	VNEAFETLKRC	48	P15172	Myoblast determination protein 1	Acts as a transcriptional activator that promotes transcription of muscle-specific target genes and plays a [illegible]	Q90477	-	Q6GN48	E5RLQ9	P16075
56	CKRKTTNADRRKA									
57	RRMKWKK	49	Q9UMQ3	Homeobox protein BarH-like 2	Transcription factor. Binds optimally to the DNA consensus sequence 5'-YYTAATGRTTTTY-3'	F6NKZ9	C3Y0U7	A0A1L8FLC4	K7F6T6	Q9W694
58	KRKx{11}KKKSKK	50	P09874	Poly [ADP-ribose] polymerase 1	Involved in the base excision repair (BER) pathway, by catalyzing the poly(ADP-ribosyl)ation of a limited number of acceptor proteins involved in chromatin architecture and in DNA metabolism.	Q5RHR0	C3Z2P3	Q2NLA7	-	A0A1L1RQW
59	KRMRNRIAASKCRKRKL	51	P05412	Transcription factor AP-1	Transcription factor that recognizes and binds to the enhancer heptamer motif 5'-TGA[CG]TCA-3'. Promotes activity of NR5A1 when phosphorylated by HIPK3 leading to increased steroidogenic gene expression upon cAMP signaling pathway stimulation. Involved in activated KRAS-mediated transcriptional activation of USP28 in colorectal cancer (CRC) cells	B0V1Q6	C3YQP4	A0JMS2	K7EY56	P18870

60	KHRKHPG	52	P46776	60S ribosomal protein L27a	RNA binding	Q6P2M0	C3ZKJ7	P47830	-	A0A1D5PSU0
61	HKKKKIRTSPTFTTPKTLRLRR QPKYPRKSAPRRNKLDHY	53	P62750	60S ribosomal protein L23a	Binds a specific region on the 26S rRNA. May promote p53/TP53 degradation possibly through the stimulation of MDM2-mediated TP53 polyubiquitination	Q6Q416	C3YD43	A0A1L8H4S4	-	E1BS06
62	KKKHPDASVNFSEFSKK	54	P09429	High mobility group protein B1	In the nucleus is one of the major chromatin-associated non-histone proteins and acts as a DNA chaperone	X1WFI9	C3YZU6	Q6GNQ5	K7FJB3	Q9YH06
63	EKSKKKK									
64	RKG AKSRLSSSKS KSRTSPYPQY	55	P81133	Single-minded homolog 1	Transcriptional factor that may have pleiotropic effects during embryogenesis and in the adult.	F1QMF7	-	A0A1L8G2Y4	-	E1C5G3
65	KGKYK EGDTGGPAVW KTRLR	56	Q00978	Interferon regulatory factor 9	Transcription factor that mediates signaling by type I IFNs (IFN-alpha and IFN-beta)	F1Q573	C3XS91	A0A1L8HPG8	K7FYQ2	Q5ZJM5
66	CRLRKC YEAGMTLGAR KLKKL	57	D3YPQ2	Androgen receptor isoform 1 transcript variant 1	DNA binding	A4GVF3	-	-	-	-
67	CRLQKCFE VGMSKESVRN DRNKKKKEVP KP	58	Q6I9R7	RARA protein	DNA binding	T2DKU4	Q8MX80	Q92019	K7F2Z9	Q90966
68	RRRHSDEN DGGQPHKRRK	59	Q09161	Nuclear cap-binding protein subunit 1	Component of the cap-binding complex (CBC), which binds cotranscriptionally to the 5'-cap of pre-mRNAs and is involved in various processes such as pre-mRNA splicing, translation regulation, nonsense-mediated mRNA decay, RNA-mediated gene silencing (RNAi) by microRNAs (miRNAs) and mRNA export	UPI0007EEE498	C3Y2C6	A0A1L8HX89	-	Q5ZJZ6
69	GKK RKRPHVFESN PSIRKR	60	Q16656	Nuclear respiratory factor 1	Transcription factor that activates the expression of the EIF2S1 (EIF2-alpha) gene	Q90X44	C3ZAI3	A0A1L8GZK2	K7GFU4	Q5ZL67
70	KKPKR	61	Q9Y6D6	Brefeldin A-inhibited guanine nucleotide-exchange protein 1	Promotes guanine-nucleotide exchange on ARF1 and ARF3	E7FGL2	C3Y295	A0A1L8FT76	K7FVJ2	A0A1D5P6U6
71	KP KKLKV	62	P55263	Adenosine kinase	ATP dependent phosphorylation of adenosine and other related nucleoside analogs to monophosphate derivatives. Serves as a potential regulator of concentrations of extracellular adenosine and intracellular adenine nucleotides.	Q6P3J9	C3XQH1	Q6DJM1	-	Q5ZMK9
72	RQHMRK AVPFQVAQK	63	Q8WTP8	Apoptosis-enhancing nuclease	Exonuclease with activity against single- and double-stranded DNA and RNA	E7F5Q4	C3YLI2	A0A1L8H0Y1	K7GFG9	A0A1L1RN59
73	MDSLLMNRRK FLYQFKNVRW AKGRRETYLC	64	Q9GZX7	Single-stranded DNA cytosine deaminase	Involved in somatic hypermutation (SHM), gene conversion, and class-switch recombination (CSR) in B-lymphocytes by deaminating C to U during transcription of Ig-variable (V) and Ig-switch (S) region DNA.	Q5YF88	-	Q4VUI3	-	E1BVI0
74	KRGKKGAV AED	65	P27695	DNA-(apurinic or apyrimidinic site) lyase	The two major activities in DNA repair and redox regulation of transcriptional factors.	F1QTR9	C3YX76	Q6IP62	-	-
75	KLV KPKNTKMKTK LRTNPY	66	Q14190	Single-minded homolog 2	Transcription factor that may be a master gene of CNS development in cooperation with Arnt. It may have pleiotropic effects in the tissues expressed during development	C1JIE2	C3XTH0	A0A1L8HCG5	-	E1BSJ1
76	RRVRLK	67	Q13568	Interferon regulatory factor 5	Transcription factor involved in the induction of interferons IFNA and INFB and inflammatory cytokines upon virus infection. Activated by TLR7 or TLR8 signaling	B0S682	C3ZAX5	A0A1L8GTJ1	K7F6Y8	E1BUT7
77	KKHRRRPSKK KRHWKPY	68	O94992	Protein HEXIM1	Transcriptional regulator which functions as a general RNA polymerase II transcription inhibitor	A5D8S8	C3Y140	-	-	-
78	YWE ECPREGKSFK AKYKLVNHIR VH	69	O60481	Zinc finger protein ZIC 3	Acts as transcriptional activator. Required in the earliest stages in both axial midline development and left-right (LR) asymmetry specification. Binds to the minimal GLI-consensus sequence 5'-GGGTGGTC-3'	Q9IAT0	-	O57311	K7FBG1	A0A1D5PRL4
79	PFPGCGKIFA RSENLKIHKR TH									
80	RV RYNPYTTRPN RRGDTWHDRD RIHVTVRRDR	70	Q9UBU9	Nuclear RNA export factor 1	Involved in the nuclear export of mRNA species bearing retroviral constitutive transport elements (CTE) and in the export of mRNA from the nucleus to the cytoplasm (TAP/NFX1 pathway)	Q0P3Y8	C3ZF40	Q6IRQ2	-	-
81	KSDKFL IIPLHSLM	71	Q9H2U1	ATP-dependent RNA helicase DHX36	Proposed to have a global role in regulating mRNA expression including transcriptional regulation and mRNA stability. Binds with high affinity to and resolves tetramolecular RNA and DNA quadruplex structures	A2BIE5	C3Y332	A0A1L8GAK8	K7FY91	A0A1D5PHB6
82	KRARCRR HQR	72	Q8NHV9	Rhox homeobox family member 1	Transcription factor maybe involved in reproductive processes. Modulates expression of target genes encoding proteins involved in processes relevant to spermatogenesis	Q7SZN8	-	-	-	-

83	RRRx{12}KRPRR	73	O14746	Telomerase reverse transcriptase	Telomerase is a ribonucleoprotein enzyme essential for the replication of chromosome termini in most eukaryotes	A2THE9	-	Q9DE32	-	A0A1D5PY42
84	RR KEKKRRRK	74	Q96B42	Transmembrane protein 18	Transcription repressor. Sequence-specific ssDNA and dsDNA binding protein, with preference for GCT end CTG repeats	Q641M3	C3XYA5	A0A1L8G500	-	Q5F410
85	KPSKGQ KRHLSTCDGQ NPPKK	75	O15226	NF-kappa-B-repressing factor	Interacts with a specific negative regulatory element (NRE) 5'-AATTCCTCTGA-3' to mediate transcriptional repression of certain NK-kappa-B responsive genes	A0A0R4IUL8	C3YZJ2	A0A1L8F334	-	F1NL52
86	KYRKGAC ENCGAMTHKK KDCFER	76	O95391	Pre-mRNA-splicing factor SLU7	Participates in the second catalytic step of pre-mRNA splicing, when the free hydroxyl group of exon I attacks the 3'-splice site to generate spliced mRNA and the excised lariat intron	F1RCR0	C3XZQ3	A0A1L8GX04	-	Q5ZIG2
87	KKRKH	77	Q9Y2G1	Myelin regulatory factor	Myelin regulatory factor: Constitutes a precursor of the transcription factor. Mediates the autocatalytic cleavage	UPI000383813F	C3Y630	Q66IV1	-	E1C867
88	R KKGK									
89	RRAMK RNARLRCPFR KGACEITRKT RR	78	O75469	Nuclear receptor subfamily 1 group I member 2	Nuclear receptor that binds and is activated by variety of endogenous and xenobiotic compounds. Transcription factor that activates the transcription of multiple genes involved in the metabolism and secretion of potentially harmful xenobiotics, drugs and endogenous compounds	B0S5T9	-	A0A1L8HIC5	-	Q9DFH3
90	LKRKLQR	79	P26367	Paired box protein Pax-6	Competes with PAX4 in binding to a common element in the glucagon, insulin and somatostatin promoters	A0A0R4IXU2	C3ZKE8	A0A1L8GJ29	K7G268	O42348
91	RKKRGGRRYR KMKER	80	Q8WWY3	U4/U6 small nuclear ribonucleoprotein Prp31	Involved in pre-mRNA splicing as component of the spliceosome. Required for the assembly of the U4/U5/U6 tri-snRNP complex, one of the building blocks of the spliceosome	B2GS36	C3Z7L6	Q5U5C5	-	-
92	RRKNYPTL ANIERKKKLK	81	Q9UHK0	Nuclear fragile X mental retardation-interacting protein 1	Binds RNA	A8KB24	C3YJZ2	A0A1L8HHI9	K7FJF1	E1C0G7
93	RKRTAPE DFLGKGPTKK	82	Q96FV9	THO complex subunit 1	Required for efficient export of polyadenylated RNA. Acts as component of the THO subcomplex of the TREX complex which is thought to couple mRNA transcription, processing and nuclear export, and which specifically associates with spliced mRNA and not with unspliced pre-mRNA	Q7SYB2	C3Y1Y4	Q498H9	-	-
94	RKRKK	83	P20264	POU domain, class 3, transcription factor 3	Transcription factor that acts synergistically with SOX11 and SOX4. Plays a role in neuronal development. Is implicated in an enhancer activity at the embryonic met-mesencephalic junction; the enhancer element contains the octamer motif (5'-ATTTGCAT-3')	A2VD21	C3ZW98	A0A1L8H8P3	K7EWD4	-
95	KRVL QVWFQNARAK FRR	84	P50458	LIM/homeobox protein Lhx2	Acts as a transcriptional activator. Stimulates the promoter of the alpha-glycoprotein gene. Transcriptional regulatory protein involved in the control of cell differentiation in developing lymphoid and neural cell types	B0R107	C3ZG21	A0A1L8F6B6	K7FB66	A0A1D5PPZ9
96	RQRK	85	Q8IWS0	PHD finger protein 6	Transcriptional regulator that associates with ribosomal RNA promoters and suppresses ribosomal RNA (rRNA) transcription	Q803W6	C3ZZY1	A0A1L8F3A3	-	V9GVP7
97	PQPKKKP	86	P04637	Cellular tumor antigen p53	Acts as a tumor suppressor in many tumor types; induces growth arrest or apoptosis depending on the physiological circumstances and cell type. Involved in cell cycle regulation as a trans-activator that acts to negatively regulate cell division by controlling a set of genes required for this process	G1K2L5	C3XPU2	P07193	K7G3P4	A0A167VDT2
98	LEKKVKKKFDW	87	P60900	Proteasome subunit alpha type-6	Component of the 20S core proteasome complex involved in the proteolytic degradation of most intracellular proteins	Q7SXN6	C3XZ66	Q6AZR7	-	F1NEQ6
99	YLTQETNKVETYKEQPLKTPG KKKKGKP	88	P12272	Parathyroid hormone-related protein	Neuroendocrine peptide which is a critical regulator of cellular and organ growth, development, migration, differentiation and survival and of epithelial calcium ion transport. Regulates endochondral bone development and epithelial-mesenchymal interactions during the formation of the mammary glands and teeth	Q1L5E7	-	A0A1L8GR30	-	P17251

100	RPRRK	89	Q05066	Sex-determining region Y protein	Promotes DNA bending. SRY HMG box recognizes DNA by partial intercalation in the minor groove. Also	Q6EJB7	-	-	-	-
101	KRPMNAFIVWSRDQRRK									
102	RRERNKMAAAKCRNRRR	90	P01100	Proto-oncogene c-Fos	Nuclear phosphoprotein which forms a tight but non-covalently linked complex with the JUN/AP-1 transcription factor. In the heterodimer, FOS and JUN/AP-1 basic regions each seems to interact with symmetrical DNA half sites.	A2CEM8	C3Y0C5	Q91639	K7GHV7	F1NFV6
103	KKKRERLD	91	P11171	Protein 4.1	Protein 4.1 is a major structural element of the erythrocyte membrane skeleton. It plays a key role in regulating membrane physical properties of mechanical stability and deformability by stabilizing spectrin-actin interaction. Recruits DLG1 to membranes. Required for dynein-dynactin complex and NUMA1 recruitment at the mitotic cell cortex during anaphase	E7F634	C3ZA69	P11434	K7FVB5	P12264
104	KRPACTLKPECVQQLLVCSQE AKK	92	P32320	Cytidine deaminase	This enzyme scavenges exogenous and endogenous cytidine and 2'-deoxycytidine for UMP synthesis	Q6P0R4	C3XPZ1	A0A1L8FHN2	K7FC96	E1C6V1
105	KRQRx{20}KKSKK	93	P49454	Centromere protein F	Required for kinetochore function and chromosome segregation in mitosis. Required for kinetochore localization of dynein, LIS1, NDE1 and NDEL1	F1Q7X5	-	A0A1L8G630	-	A0A1L1RMT7
106	KRSAEGSNPPKPLKKLR	94	P06400	Retinoblastoma-associated protein	Key regulator of entry into cell division that acts as a tumor suppressor. Promotes G0-G1 transition when phosphorylated by CDK3/cyclin-C. Acts as a transcription repressor of E2F1 target genes	B3DI38	C3XU15	A0A1L8EML3	K7FQ20	Q90600
107	KRKCSQTQCPRKVIK	95	P29590	Protein PML	Functions via its association with PML-nuclear bodies (PML-NBs) in a wide range of important cellular processes, including tumor suppression, transcriptional regulation, apoptosis, senescence, DNA damage response, and viral defense mechanisms	F1RBJ0	-	-	-	A0A1L1RZK6
108	PGRKRKW	96	Q9HAN9	Nicotinamide/nicotinic acid mononucleotide adenylyltransferase 1	Catalyzes the formation of NAD+ from nicotinamide mononucleotide (NMN) and ATP	E9QH56	C3Y2B3	A0A1L8FKV8	K7G6T7	R4GGL5
109	ILIKRRQQ KIRKYTMRRL LQ	97	P04626	Receptor tyrosine-protein kinase erbB-2	Protein tyrosine kinase that is part of several cell surface receptor complexes, but that apparently needs a coreceptor for ligand binding. Essential component of a neuregulin-receptor complex, although neuregulins do not interact with it alone	Q7SZF7	C3YZ48	A0A1L8FRS8	-	A0A1D5PEH0
110	NKL PRRRVNIFGE ELERLLKKKY	98	P50616	Protein Tob1	Anti-proliferative protein; the function is mediated by association with deadenylase subunits of the CCR4-NOT complex	Q7SZR1	C3Z7P3	A0A1L8EUN5	K7FHV8	Q90VW0
111	RRQQEQLR YEQERRPGRR	99	Q14151	Scaffold attachment factor B2	Binds to scaffold/matrix attachment region (S/MAR) DNA. Can function as an estrogen receptor corepressor and can also inhibit cell proliferation.	Q803X6	C3Z0S4	A0A1L8HWX4	K7FD64	H9KZ12
112	ELCRRRSLLE LHKRRKALTE	100	P53350	Serine/threonine-protein kinase PLK1	Serine/threonine-protein kinase that performs several important functions throughout M phase of the cell cycle, including the regulation of centrosome maturation and spindle assembly, the removal of cohesins from chromosome arms, the inactivation of anaphase-promoting complex/cyclosome (APC/C) inhibitors, and the regulation of mitotic exit and cytokinesis	Q4KMI8	C3ZJT7	P70032	K7FID3	F1NB57
113	SKTFAMK ILKKRHIVDT RQQ	101	Q13976-2	cGMP-dependent protein kinase 1	Serine/threonine protein kinase that acts as key mediator of the nitric oxide (NO)/cGMP signaling pathway	A0A286Y9K1	C3Z4R1	Q6GPV8	-	E1C4W9
114	RKKEA PGPREELRSR GR	102	P54259	Atrophin-1	Transcriptional corepressor. Recruits NR2E1 to repress transcription	UPI00038385C7	-	A0A1L8FIG3	K7G1I3	-
115	RKPDRRKR SR	103	Q92560	Ubiquitin carboxyl-terminal hydrolase BAP1	Deubiquitinating enzyme that plays a key role in chromatin by mediating deubiquitination of histone H2A and HCFC1. Catalytic component of the PR-DUB complex, a complex that specifically mediates deubiquitination of histone H2A monoubiquitinated at 'Lys-119' (H2AK119ub1)	F1QJ12	C4A0D9	A0A1L8GHI0	-	Q5F3N6
116	KRFRREFGD SKRVFGERRR ND	104	Q9NRA8	Eukaryotic translation initiation factor 4E transporter	Nucleoplasmic shuttling protein, which inhibits translation initiation. Mediates the nuclear import of EIF4E by a piggy-back mechanism	E7FDR0	C3YCP5	A0A1L8I094	-	E1BTK8

117	HRKR	105	Q7Z6G8	Ankyrin repeat and sterile alpha motif domain-containing protein 1B	Ephrin receptor binding	A0A0R4IE55	C3ZFZ7	A0A1L8H6M2	-	A0A1D5PKC1
118	KRASEDT TSGSPPKKSS AGPKR	106	Q9BZZ5	Apoptosis inhibitor 5	Antiapoptotic factor that may have a role in protein assembly. Negatively regulates ACIN1. By binding to ACIN1, it suppresses ACIN1 cleavage from CASP3 and ACIN1-mediated DNA fragmentation. Also known to efficiently suppress E2F1-induced apoptosis. Its depletion enhances the cytotoxic action of the chemotherapeutic drugs	R4GDP5	C4A049	Q6DDM4	-	Q5ZMW3
119	RRPR RRTFPGVASR RNPERR	107	Q9BWT1	Cell division cycle-associated protein 7	Participates in MYC-mediated cell transformation and apoptosis; induces anchorage-independent growth and clonogenicity in lymphoblastoid cells. Insufficient to induce tumorigenicity when overexpressed but contributes to MYC-mediated tumorigenesis. May play a role as transcriptional regulator	B0S4Y9	C3Y254	A0JMU9	K7FL67	A0A1D5PK49
120	KEL LKHKFILRNA KK	108	Q9Y6E0	Serine/threonine-protein kinase 24	Serine/threonine-protein kinase that acts on both serine and threonine residues and promotes apoptosis in response to stress stimuli and caspase activation	A0A0R4IN78	C3YYD0	A0A1L8H969	K7FJN7	A0A1D5NWG8
121	KRRK	109	Q14678	KN motif and ankyrin repeat domain-containing protein 1	Involved in the control of cytoskeleton formation by regulating actin polymerization	E9QCJ6	C3ZZY7	A0A1L8HWJ6	K7G0R3	F1NA35
122	N ESTLKSIMKK KDGNKDSNGA KK									
123	KSKKRIR	110	Q6NYC1	Bifunctional arginine demethylase and lysyl-hydroxylase JMJD6	Dioxygenase that can both act as a histone arginine demethylase and a lysyl-hydroxylase	Q6PFM0	C3ZFA3	A0A1L8EUJ7	K7FSE8	Q5ZMK5
124	KRKYR									
125	RRKKRR									
126	PKRRK									
127	KRRP									
128	RKE KVKRE	111	P34947	G protein-coupled receptor kinase 5	Serine/threonine kinase that phosphorylates preferentially the activated forms of a variety of G-protein-coupled receptors (GPCRs	Q1XHM0	C3Y5N3	Q6DCW4	K7FMD2	Q5ZKL2
129	RQQRKR	112	Q14980	Nuclear mitotic apparatus protein 1	Microtubule (MT)-binding protein that plays a role in the formation and maintenance of the spindle poles and the alignement and the segregation of chromosomes during mitotic cell division	A0A0R4IH76	-	A0A1L8HJJ6	-	D8MIU8
130	KRKRKNSR VTFSEDDEI	113	Q12972	Nuclear inhibitor of protein phosphatase 1	Inhibitor subunit of the major nuclear protein phosphatase-1 (PP-1). It has RNA-binding activity but does not cleave RNA and may target PP-1 to RNA-	A5PF58	-	Q6P9H8	K7GAA8	F1N9I1
131	RFRNMVQTAV VPVKKKRVEG									
132	KRKR	114	Q6DJT9	Zinc finger protein PLAG1	Transcription factor whose activation results in up-regulation of target genes, such as IGFII, leading to uncontrolled cell proliferation: when overexpressed in cultured cells, higher proliferation rate and transformation are observed	Q29YB2	C3ZWC1	A0A1L8FZ09	K7GIK1	Q58NQ5
133	KKKYT R	115	Q13177	Serine/threonine-protein kinase PAK 2	Serine/threonine protein kinase that plays a role in a variety of different signaling pathways including cytoskeleton regulation, cell motility, cell cycle progression, apoptosis or proliferation. Acts as downstream effector of the small GTPases CDC42 and RAC1	Q6DG42	C3Z873	Q6PAZ2	K7FAU6	E1C3P6
134	RKSK	116	Q9H2X6	Homeodomain-interacting protein kinase 2	Serine/threonine-protein kinase involved in transcription regulation, p53/TP53-mediated cellular	F1Q4N0	C3Y5H6	A0A1L8H6D1	K7GA98	F1P4G3
135	KRVK									
136	KKAILKKKEE K	117	O00141	Serine/threonine-protein kinase Sgk1	Serine/threonine-protein kinase which is involved in the regulation of a wide variety of ion channels, membrane transporters, cellular enzymes, transcription factors, neuronal excitability, cell growth, proliferation, survival, migration and apoptosis	Q7ZTW4	C3Y291	Q6GPN6	K7G4F8	Q6U1I9
137	RGKK K	118	Q9UBP0	Spastin	ATP-dependent microtubule severing protein that specifically recognizes and cuts microtubules that are	Q6NW58	C3YR60	Q6AZT2	K7EXQ8	Q5ZK92
138	RK KK									
139	KRP R	119	Q9UQ84	Exonuclease 1	5'->3' double-stranded DNA exonuclease which may also possess a cryptic 3'->5' double-stranded DNA exonuclease activity	Q803U7	C3Z4R4	Q9W6K2	K7G1H9	E1C1G7

140	KLRRR	120	O95239	Chromosome-associated kinesin KIF4A	Motor protein that translocates PRC1 to the plus ends of interdigitating spindle microtubules during the metaphase to anaphase transition, an essential step for the formation of an organized central spindle midzone and midbody and for successful cytokinesis. May play a role in mitotic chromosomal positioning and bipolar spindle stabilization	F8W409	C3Z125	Q91784	K7FYR1	O95239
141	K VKTKKSTK	121	P42338	Phosphatidylinositol 4,5-bisphosphate 3-kinase catalytic subunit beta isoform	Phosphoinositide-3-kinase (PI3K) that phosphorylates PtdIns (Phosphatidylinositol), PtdIns4P (Phosphatidylinositol 4-phosphate) and PtdIns(4,5)P2 (Phosphatidylinositol 4,5-bisphosphate) to generate phosphatidylinositol 3,4,5-trisphosphate (PIP3).	F1QAD7	C3XYJ1	A0A1L8G3Z7	K7F3A1	E1C093
142	TKRPRW	122	Q86W56	Poly(ADP-ribose) glycohydrolase	Poly(ADP-ribose) synthesized after DNA damage is only present transiently and is rapidly degraded by poly(ADP-ribose) glycohydrolas	E7F2B6	C3ZES4	Q4KLP9	K7FAS7	F1NY76
143	KR KTKISRKTR	123	O00443	Phosphatidylinositol 4-phosphate 3-kinase C2 domain-containing subunit alpha	Generates phosphatidylinositol 3-phosphate (PtdIns3P) and phosphatidylinositol 3,4-bisphosphate (PtdIns(3,4)P2) that act as second messengers	F1QWN6	C3ZMJ4	A0A1L8GQ56	-	E1C1G0
144	KQKQRF EEKRFK	124	Q8NHY2	E3 ubiquitin-protein ligase COP1	E3 ubiquitin-protein ligase that mediates ubiquitination and subsequent proteasomal degradation of target	A4QP94	C3Y342	A0A1L8GMK1	K7FZ14	F1NQ64
145	GS RKR									
146	KRKK	125	Q13352	Centromere protein R	Transcription coregulator that can have both coactivator and corepressor functions	X1WGM0	-	A0A1L8GLX9	-	F1NP14
147	RKKRK GK	126	Q14118	Dystroglycan	The dystroglycan complex is involved in a number of processes including laminin and basement membrane assembly, sarcolemmal stability, cell survival, peripheral nerve myelination, nodal structure, cell migration, and epithelial polarization	Q8JHU7	C3YV90	A0A1L8GP44	K7GFA6	A4VAR9
148	KKKGK	127	O00757	Fructose-1,6-bisphosphatase isozyme 2	Catalyzes the hydrolysis of fructose 1,6-bisphosphate to fructose 6-phosphate in the presence of divalent cations and probably participates in glycogen synthesis from carbohydrate precursors, such as lactate.	Q68EJ6	-	Q7SZA0	K7F7B3	F1NYI2
149	R SAGCRKGKHK RKK	128	P56645	Period circadian protein homolog 3	Kinase binding. Ubiquitin protein ligase binding	Q9I8L4	-	A0A1L8FGK4	-	E1C8E2
150	RRKR	129	Q14934	Nuclear factor of activated T-cells, cytoplasmic 4	Plays a role in the inducible expression of cytokine genes in T-cells, especially in the induction of the IL-2	E7F059	C3YFD8	A0A1L8HZL8	K7FYL4	A0A1D5NT38
151	KRR Y									
152	PPVKRERTS	130	Q9H6Z4	Ran-binding protein 3	Acts as a cofactor for XPO1/CRM1-mediated nuclear export, perhaps as export complex scaffolding protein	Q6PCR4	C3ZFL9	Q6IP83	-	F1NJ42
153	PQSRKKLR	131	P61244	Protein max	Transcription regulator. Forms a sequence-specific DNA-binding protein complex with MYC or MAD which recognizes the core sequence 5'-CAC[GA]TG-3'.	Q6NY29	C3XRZ9	Q07016	K7FRK5	P52162
154	KVTKRKHDNEGSGSKRPK	132	P12956	X-ray repair cross-complementing protein 6	Single-stranded DNA-dependent ATP-dependent helicase. Has a role in chromosome translocation.	F1QZ60	C3Z1F8	Q6AZR6	K7GE42	O93257
155	RAIKRRPGLDFDDDGEGNSKFLR	133	P27540	Aryl hydrocarbon receptor nuclear translocator	Required for activity of the Ah (dioxin) receptor. This protein is required for the ligand-binding subunit to translocate from the cytosol to the nucleus after ligand binding.	Q9DG12	C3Y283	A0A1L8H0U4	K7G3H3	E1C1C5
156	RVHPYQR	134	Q96PU8	Protein quaking	RNA-binding protein that plays a central role in myelinization	Q6P104	C3XVZ1	A0A1L8G7T1	-	A0A1D5P2B1
157	KKQTTLAFKPIKKGKKR	135	P11388	DNA topoisomerase 2-alpha	Control of topological states of DNA by transient breakage and subsequent rejoining of DNA strands. Topoisomerase II makes double-strand breaks. Essential during mitosis and meiosis for proper segregation of daughter chromosomes. May play a role in regulating the period length of ARNTL/BMAL1 transcriptional oscillation	Q1LUT2	C3YYJ6	A0A1L8FW96	K7FJR3	E1BZ19
158	KKEKKKSKK	136	O60832	H/ACA ribonucleoprotein complex subunit DKC1	Catalytic subunit of H/ACA small nucleolar ribonucleoprotein (H/ACA snoRNP) complex, which catalyzes pseudouridylation of rRNA	F1Q749	C3Y7R6	-	-	Q5ZJH9
159	KKx{15}KKRK	137	P46063	ATP-dependent DNA helicase Q1	DNA helicase that may play a role in the repair of DNA that is damaged by ultraviolet light or other mutagens. Exhibits a magnesium-dependent ATP-dependent DNA-helicase activity that unwinds single- and double-stranded DNA in a 3'-5' direction	Q498Z7	C3YKR1	Q32NN1	K7F4Y6	Q8AYS4

160	SGYG KVSRRGGHQN SYKPY	138	Q14103	Heterogeneous nuclear ribonucleoprotein D0	Binds with high affinity to RNA molecules that contain AU-rich elements (AREs) found within the 3'-UTR of many proto-oncogenes and cytokine mRNAs.	Q7SXN2	C3ZWI1	Q7ZYE9	K7FUP0	F6QGM9
161	QR KVKPQPPLSD AYLSGMPAKR RKT	139	P46379	Large proline-rich protein BAG6	ATP-independent molecular chaperone preventing the aggregation of misfolded and hydrophobic patches-containing proteins.	A3KPW9	-	Q6PA26	-	-
162	KRKMDETDA SSAVKVKR	140	Q13148	TAR DNA-binding protein 43	DNA and RNA-binding protein which regulates transcription and splicing.	Q802C7	C3ZIE9	A0A1L8FFB1	K7FJ67	Q5ZLN5
163	RGGRGG GDRGGFGPGK MDSRGEHRQD RRERPY	141	P35637	RNA-binding protein FUS	Binds both single-stranded and double-stranded DNA and promotes ATP-independent annealing of complementary single-stranded DNAs and D-loop formation in superhelical double-stranded DNA. May play a role in maintenance of genomic integrity.	F1R0M4	C3Z8P8	Q7ZXQ2	-	Q6J4Y8
164	KKPK	142	P05230	Fibroblast growth factor 1	Plays an important role in the regulation of cell survival, cell division, angiogenesis, cell differentiation and cell migration	Q5TLE2	-	A0A1L8HW95	K7FNQ6	P48801
165	PSKRQRV	143	P07910	Heterogeneous nuclear ribonucleoproteins C1/C2	Binds pre-mRNA and nucleates the assembly of 40S hnRNP particles	Q802Y4	C3Z5T3	A0A1L8I0B0	K7FZZ4	-
166	KRKRPR	144	O60682	Musculin	Transcription repressor capable of inhibiting the transactivation capability of TCF3/E47. May play a role in regulating antigen-dependent B-cell differentiation.	Q32PV5	C3Y214	A0A1L8G8Q2	K7F6Z1	A0A1D5PSY8
167	KKKKKK	145	Q96GQ7	Probable ATP-dependent RNA helicase DDX27	Probable ATP-dependent RNA helicase. Component of the nucleolar ribosomal RNA (rRNA) processing machinery that regulates 3' end formation of ribosomal 47S rRNA	Q6DRN0	C3ZBT1	Q4V836	-	Q5ZLD0
168	RKRRR	146	Q9H8V3	Protein ECT2	Guanine nucleotide exchange factor (GEF) that catalyzes the exchange of GDP for GTP	A0A0R4IKD5	C3YJI7	Q5XGV4	K7FLF0	E1BWT7
169	PRKRP									
170	PSQQKRK	147	Q92547	DNA topoisomerase 2-binding protein 1	Required for DNA replication. Plays a role in the rescue of stalled replication forks and checkpoint control	F1R135	C3Z1H4	A0A1L8FWJ6	K7FLF0	A0A1D5P3M9
171	KRPR									
172	RKRARAEKKA LKKKKKI	148	Q15020	Squamous cell carcinoma antigen recognized by T-cells 3	U6 snRNP-binding protein that functions as a recycling factor of the splicing machinery. Promotes the initial reassembly of U4 and U6 snRNPs following their ejection from the spliceosome during its maturation	Q6ZM52	C3Y679	A0A1L8HZW2	-	A0A1D5P378
173	SGKRGGRKRR SSRRSAGGGA GPGGAAGGGV GGGDEPGSPA QGKRGKKSAG C	149	Q15672	Twist-related protein 1	Acts as a transcriptional regulator.	Q9PTE3	C3YC05	A0A1L8FR58	K7G204	Q9PVG4
174	LKKD KSRKRSYSPD GKESPSDKKS KTD	150	P43243	Matrin-3	May play a role in transcription or may interact with other nuclear matrix proteins to form the internal fibrogranular network	Q800H6	-	-	K7G839	Q8UWC5
175	KKKLKKVD									
176	RRGRK RKAEKQ	151	O75475	PC4 and SFRS1-interacting protein	Transcriptional coactivator involved in neuroepithelial stem cell differentiation and neurogenesis.	F8W309	C3Z0T2	Q5XXA8	K7GK32	R4GK64
177	NN GEQIIRRRTR KRLNPEAL	152	Q9UHF7-2	Zinc finger transcription factor Trps1	Transcriptional repressor. Binds specifically to GATA sequences and represses expression of GATA-regulated genes at selected sites and stages in vertebrate development.	D4P0Z5	-	A0A1L8FZU9	K7G5X8	A0A1D5PND7
178	MGLRTAKKRG LGGGGKWKRE	153	P28329	Choline O-acetyltransferase	Catalyzes the reversible synthesis of acetylcholine (ACh) from acetyl CoA and choline at cholinergic synapses	Q642H6	C3ZX27	A0A1L8GMF7	K7GIT1	Q90YJ9
179	KRAADE VLAEAKKPR	154	Q6P1J9	Parafibromin	Tumor suppressor probably involved in transcriptional and post-transcriptional control pathways	X1WHN7	C3ZG24	A0A1L8GND2	-	Q5ZLM0
180	LKRAGSPEVQ GAMGSPAPKR	155	O43593	Lysine-specific demethylase hairless	Histone demethylase that specifically demethylates both mono- and dimethylated 'Lys-9' of histone H3. May act as a transcription regulator controlling hair biology (via targeting of collagens), neural activity, and cell cycle	UPI0000F2058B	-	-	-	-
181	YRK CLQAGMNLEA RKTKKKIKGI QQAT	156	P04150	Glucocorticoid receptor	Has a dual mode of action: as a transcription factor that binds to glucocorticoid response elements (GRE), both for nuclear and mitochondrial DNA, and as a modulator of other transcription factors	Q1XHK0	-	A0A1L8GV81	K7GAE8	Q38HX7
182	KTR REKRAR	157	Q9Y266	Nuclear migration protein nudC	Necessary for correct formation of mitotic spindles and chromosome separation during mitosis. Necessary for cytokinesis and cell proliferation	Q7ZVD2	C3ZLJ1	Q6IRP6	-	Q5ZIN1

183	PVAQKRK	158	P41218	Myeloid cell nuclear differentiation antigen	May act as a transcriptional activator/repressor in the myeloid lineage. Plays a role in the granulocyte/monocyte cell-specific response to interferon. Stimulates the DNA binding of the transcriptional repressor protein YY1	UPI00038371A7	-	-	-	-
184	RRAAPT TPPPPVKRRD RE	159	Q9NZQ3	NCK-interacting protein with SH3 domain	Has an important role in stress fiber formation induced by active diaphanous protein homolog 1 (DRF1)	UPI0007EEE691	C3Z2V1	A0A1L8GHQ7	-	E1C7M3
185	KKKSRK	160	Q01954	Zinc finger protein basonuclin-1	Transcriptional activator	A0A0G2KIM8	C3ZGK9	A0A1L8GST0	K7FIP4	E1C4G7
186	RKRKKRKKGE KQIPGEEKGR KRRRVKEDKK KRDRDR	161	Q9NPI1	Bromodomain-containing protein 7	Acts both as coactivator and as corepressor	B0S7E1	C3YFI8	Q802C4	-	Q5ZKG2
187	KG VKRKADTTTP	162	Q58F21	Bromodomain testis-specific protein	Testis-specific chromatin protein that specifically binds histone H4 acetylated at 'Lys-5' and 'Lys-8' (H4K5ac and H4K8ac, respectively) and plays a key role in spermatogenesis	F1QQP6	C3ZPB3	A1L1G8	-	E1C8U8
188	K RRIRRRPR	163	P41002			Q6NYX6	C3Z7N7	Q7T0L6	K7G0H4	A0A1D5PBK0
189	RRT KRKR									
190	KLKRR	164	Q49MI3	Ceramide kinase-like protein	Has no detectable ceramide-kinase activity. Overexpression of CERKL protects cells from apoptosis in oxidative stress conditions	A5WVZ0	C3YV82	Q6GLV1	K7G4B6	Q5F3H3
191	RRTTRRHP NTQQRASKKK PK	165	Q15021	Condensin complex subunit 1	Regulatory subunit of the condensin complex, a complex required for conversion of interphase chromatin into mitotic-like condense chromosomes.	B0V123	C3XPS2	Q9YHY6	K7GDZ8	Q5F475
192	RKRL KSK	166	Q13309	S-phase kinase-associated protein 2	Substrate recognition component of a SCF (SKP1-CUL1-F-box protein) E3 ubiquitin-protein ligase complex which mediates the ubiquitination and subsequent proteasomal degradation of target proteins involved in cell cycle progression, signal transduction and transcription	A3KP53	C3YM20	Q075B2	-	F7BEK8
193	KKSKRGR	167	Q9UBS0	Ribosomal protein S6 kinase beta-2	Phosphorylates specifically ribosomal protein S6.	Q802Y7	C3YDD4	Q9W6Y9	K7FKX2	Q5ZLW5
194	KKKMKKH KNKSEAKKRK IEFLRPGYKA KRQK	168	O75691	Small subunit processome component 20 homolog	Involved in 18S pre-rRNA processing. Associates with U3 snoRNA.	A0A0R4IQW7	C3ZQ27	-	K7GES8	E1BVC5
195	KVLGVNVMLR K	169	P29373	Cellular retinoic acid-binding protein 2	Transports retinoic acid to the nucleus. Regulates the access of retinoic acid to the nuclear retinoic acid receptors.	A4VCG2	C3Y8B2	A0A1L8FCB0	K7FJ80	R4GL88
196	G KMDKGEHRQE RRDRPY	170	Q01844	RNA-binding protein EWS	Might normally function as a transcriptional repressor	Q4QRG0	C3Z8P8	Q2VPG6	-	A0A1D5NUX9
197	RKRR	171	Q14493	Histone RNA hairpin-binding protein	RNA-binding protein involved in the histone pre-mRNA processing	A1L2F4	C3Y0M4	A0A1L8HT76	-	Q6B7Z7
198	RYKRK									
199	KR GLNSSFETSP KKVKW	172	Q8IV63	Inactive serine/threonine-protein kinase VRK3	Inactive kinase that suppresses ERK activity by promoting phosphatase activity of DUSP3 which specifically dephosphorylates and inactivates ERK in the nucleus	Q5BLE9	C3XZ03	A0A1L8GK79	K7G2S1	F1NDK1
200	RRKSI KKKR	173	Q15303	Receptor tyrosine-protein kinase erbB-4	Tyrosine-protein kinase that plays an essential role as cell surface receptor for neuregulins and EGF family members and regulates development of the heart, the central nervous system and the mammary gland, gene transcription, cell proliferation, differentiation, migration and apoptosis	A0A0A0MPK4	C3YZ4	A0A1L8FRS8	-	A0A1D5PKH0
201	STKKRER	174	O60934	Nibrin	Component of the MRE11-RAD50-NBN (MRN complex) which plays a critical role in the cellular response to DNA damage and the maintenance of chromosome integrity	Q5I2W8	C3YFG1	Q6XV80	-	A0A1D5P1G5
202	K RKR	175	Q96C86	m7GpppX diphosphatase	Decapping scavenger enzyme that catalyzes the cleavage of a residual cap structure following the degradation of mRNAs by the 3'->5' exosome-mediated mRNA decay pathway	F1R776	C3ZTI1	Q4KLD2	-	-
203	RKRKPS	176	Q7Z5L9	Interferon regulatory factor 2-binding protein 2	Acts as a transcriptional corepressor in a IRF2-dependent manner; this repression is not mediated by histone deacetylase activities	Q6NZT6	-	A0A1L8G830	K7EX81	A0A1D5PGK9
204	SDPKGPPAKI ARLEQNGSPL	177	Q01826	DNA-binding protein SATB1	Binds to DNA at special AT-rich sequences, the consensus SATB1-binding sequence (CSBS), at nuclear matrix- or scaffold-associated regions.	E1ANH4	-	A0A1L8FW95	K7GJC9	E1BZL3

205	KRQRKRR	178	P10244	Myb-related protein B	Transcription factor involved in the regulation of cell survival, proliferation, and differentiation. Transactivates the expression of the CLU gene.	Q4KMF7	C3ZI75	Q08759	K7GDA9	Q03237
206	RRR	179	Q12778	Forkhead box protein O1	Transcription factor that is the main target of insulin signaling and regulates metabolic homeostasis in response to oxidative stress	A3RK75	C3YI59	A0A1L8HIX1	K7EZG6	F1NNE8
207	RKRTPRVDGQT GENDMNKRRR K	180	O94851	[F-actin]-monooxygenase MICAL2	Nuclear monooxygenase that promotes depolymerization of F-actin by mediating oxidation of specific methionine residues on actin to form methionine-sulfoxide, resulting in actin filament disassembly and preventing repolymerization	A0A0A0MPT7	C3Z6G9	-	-	Q5F3F3
208	KKRKK	181	Q969P5	F-box only protein 32	Substrate recognition component of a SCF (SKP1-CUL1-F-box protein) E3 ubiquitin-protein ligase complex which mediates the ubiquitination and subsequent proteasomal degradation of target proteins. Probably recognizes and binds to phosphorylated target proteins during skeletal muscle atrophy. Recognizes TERF1.	Q7ZTY2	C3Y232	A0A1L8FTW6	K7F351	Q5ZHM6
209	K AFFQSEDSPS PKRQRLSHSV F	182	Q9H0F5	E3 ubiquitin-protein ligase RNF38	Acts as an E3 ubiquitin-protein ligase able to ubiquitinate p53/TP53 which promotes its relocalization to discrete foci associated with PML	E7FDX7	-	A0A1L8GY19	K7GE35	F1NND0
210	RNRRSPP VRRQRGRRD R									
211	RKKNKY RPISHTPS	183	Q96K30	RBPJ-interacting and tubulin-associated protein 1	Tubulin-binding protein that acts as a negative regulator of Notch signaling pathway. Shuttles between the cytoplasm and the nucleus and mediates the nuclear export of RBPJ/RBPSUH, thereby preventing the interaction between RBPJ/RBPSUH and NICD product of Notch proteins (Notch intracellular domain), leading to down-regulate Notch-mediated transcription. May play a role in neurogenesis.	Q1JPZ6	C3Y4L6	A0A1L8I064	-	-
212	K KRGKKKKRGR	184	Q9HBH9	MAP kinase-interacting serine/threonine-protein kinase 2	Serine/threonine-protein kinase that phosphorylates SFPQ/PSF, HNRNPA1 and EIF4E	Q7ZVV9	C3YM17	A0A1L8HWS4	K7F5G5	F1NCK3
213	RKRKK	185	Q16666	Gamma-interferon-inducible protein 16	Binds double-stranded DNA. Binds preferentially to supercoiled DNA and cruciform DNA structures	UPI000383722A	-	-	-	-
214	KRK K									
215	KEK									
216	K GSK									
217	RKRGRGRPKG RGRGRPRGRP RGSKR	186	Q9UKY1	Zinc fingers and homeoboxes protein 1	Acts as a transcriptional repressor. Increases DNMT3B-mediated repressive transcriptional activity when DNMT3B is tethered to DNA. May link molecule between DNMT3B and other co-repressor proteins	A5D8R5	C3Z1B2	A0A1L8G021	K7FK68	A0A1D5PKX3
218	RKA KRAR	187	Q86WB0	Nuclear-interacting partner of ALK	Essential component of a SCF-type E3 ligase complex, SCF(NIPA), a complex that controls mitotic entry by mediating ubiquitination and subsequent degradation of cyclin B1 (CCNB1)	Q08BA2	-	A0A1L8GTM4	-	E1C3I1
219	RKK	188	Q9UNZ2	NSFL1 cofactor p47	Together with UBXN2B/p37, regulates the centrosomal levels of kinase AURKA/Aurora A during [illegible]	Q5U3U1	C3Y288	Q7ZYT4	K7G8S4	A0A1L1RX58
220	KRQH									
221	KPNKRK	189	P51858	Hepatoma-derived growth factor	Heparin-binding protein, with mitogenic activity for fibroblasts. Acts as a transcriptional repressor	Q5XXA7	C3Z0T2	A0A1L8HW52	K7FIL0	A0A1D5PAE4
222	KRRAGD LLEDSPKRPK									
223	KRQEME SGITTPPKMR R	190	P21359	Neurofibromin	Stimulates the GTPase activity of Ras. NF1 shows greater affinity for Ras GAP, but lower specific activity. May be a regulator of Ras activity.	E7FFY1	C3ZV81	A0A1L8HGF2	-	A0A1D5P1U6
224	KK KGRTQKEKKA ARARSKGK	191	Q9UNL4	Inhibitor of growth protein 4	Component of the HBO1 complex which has a histone H4-specific acetyltransferase activity, a reduced activity toward histone H3 and is responsible for the bulk of histone H4 acetylation in vivo.	Q4VBS0	C3ZSF3	A0A1L8FDZ0	K7FSP4	Q5ZKY4
225	KRRRRR RREKRKR	192	Q96GM8	Target of EGR1 protein 1	Inhibits cell growth rate and cell cycle. Induces CDKN1A expression as well as TGF-beta expression. Mediates the inhibitory growth effect of EGR1. Involved in the maturation of snRNAs and snRNA 3'-tail processing	B8A5U4	C3YIK6	A0A1L8GFN0	-	F1N9P8
226	GKN EAKKRK	193	P52739	Zinc finger protein 131	Plays a role during development and organogenesis as well as in the function of the adult central nervous [illegible]	Q7SXM6	-	A0A1L8I2R2	-	E1C6B5
227	KK QRTGKKIH									

228	KG RRGRLPSKPK	194	P43354	Nuclear receptor subfamily 4 group A member 2	Transcriptional regulator which is important for the differentiation and maintenance of meso-diencephalic dopaminergic (mdDA) neurons during development. It is crucial for expression of a set of genes such as SLC6A3, SLC18A2, TH and DRD2 which are essential for development of mdDA neurons	Q6GMG3	C3YEV5	Q7T0V3	K7FLA5	A0A1D5NYR5
229	KKKP AK	195	Q9BRS8	La-related protein 6	Regulates the coordinated translation of type I collagen alpha-1 and alpha-2 mRNAs, CO1A1 and CO1A2. Stabilizes mRNAs through high-affinity binding of a stem-loop structure in their 5' UTR. This regulation requires VIM and MYH10 filaments, and the helicase DHX9	Q6PHD4	C3ZKS9	A0A1L8H0A5	-	F1NWE6
230	RR R	196	Q9HC62	Sentrin-specific protease 2	Protease that catalyzes two essential functions in the SUMO pathway. The first is the hydrolysis of an alpha-	E7F9K9	C3XYA3	Q804S7	K7G2N0	A0A1D5PCU8
231	PAKRP R									
232	KRRH	197	Q12968	Nuclear factor of activated T-cells, cytoplasmic 3	Acts as a regulator of transcriptional activation. Plays a role in the inducible expression of cytokine genes in T-	E7FE12	C3YFD8	A0A1L8GK58	K7FYL4	A0A1D5P8S9
233	KRKK									
234	KRK	198	O43148	mRNA cap guanine-N7 methyltransferase	Catalytic subunit of the mRNA-capping methyltransferase RNMT:RAMMET complex that methylates the N7 position of the added guanosine to the 5'-cap structure of mRNAs	Q1MTD3	C3ZV89	A0A1L8FYD5	K7FIZ9	E1BYM1

***In silico* predicted NLSs**

1	RKKRR	1	Q9UER7	Death domain-associated protein 6	Transcription corepressor known to repress transcriptional potential of several sumoylated	Q567Z8	-	Q2KHQ3	-	-
2	PCK KSRK									
3	PKPS	2	P54253	Ataxin-1	Chromatin-binding factor that repress Notch signaling in the absence of Notch intracellular domain by acting as a CBF1 corepressor.	Q1L966	-	A0A1L8FSD1	-	E1BZ23
4	VKRRRM	3	Q03001	Dystonin	Cytoskeletal linker protein. Acts as an integrator of intermediate filaments, actin and microtubule cytoskeleton networks.	A0A0G2KQI8	C3YBC4	-	-	A0A1D5P0Y6
5	K KELPSHVQAM HKRKEFL	4	Q7Z333	Probable helicase senataxin	Probable RNA/DNA helicase involved in diverse aspects of RNA metabolism and genomic integrity.	E7FAV9	C3ZM47	A0A1L8HDV6	-	Q5ZKD7
6	RARLA NTQGKKAKRK AREKQLEEAR RLAALQKRRE LRAAGIEIQK KRKRKRGVDY NAEIPFEKKP ALGFYDTSEE NYQALDADFR KLRQQDLDGE LRSEKEGRDR KKDKQHLKRK K	5	Q99459	Cell division cycle 5-like protein	DNA-binding protein involved in cell cycle control. May act as a transcription activator.	E9QIC1	C3YHB1	A1A5J6	K7G2Y2	E1C6A9
7	KKLEV NEAELLRRRN FK	6	Q6NZI2	Caveolae-associated protein 1	Plays an important role in caveolae formation and organization. Essential for the formation of caveolae in	E7F0K3	-	A0A1L8EKH0	K7GED2	Q90885
8	KKAFSKEK MEKTKVRTR									
9	RKKRR	7	Q0VD86	Protein INCA1	cyclin-dependent protein serine/threonine kinase inhibitor activity	E9QDB1	-	-	-	-
10	QVGA GGEPLDACYL KALEGFVMVL	8	Q9Y2N7	Hypoxia-inducible factor 3-alpha	Acts as a transcriptional regulator in adaptive response to low oxygen tension.	Q6EHI4	-	Q6PI54	K7FUG2	Q9YIB9
11	LQA NGPVAKKAKK KA	9	Q14978	Nucleolar and coiled-body phosphoprotein 1	Nucleolar protein that acts as a regulator of RNA polymerase I by connecting RNA polymerase I with enzymes responsible for ribosomal processing and modification	B8JIY2	-	Q91803	K7GC40	A0A1D5PUU2
12	TKNSSNK PAVTTKSPAV KPAAAPKQPV GGGQKLLTRK ADSSSSEEES SSSEEEKTKK MVATTKPKAT AKAALSLPAK QAPQGSRDSS SDSDSSSSEE EEEKTSKSAV KKKPQKVAGG AAPSKPASAK KGKAESSNSS SSDDSSEEEE EKLKGKGSPR PQAPKANGTS ALTAQNGKAA KNSEEEEEEK KKAAVVVSKS GSLKKRK									
13	KIKLQTPNT FPKRKKG									
14	LSPSL SPL	10	Q9Y261	Hepatocyte nuclear factor 3-beta	Transcription factor that is involved in embryonic development, establishment of tissue-specific gene expression and regulation of gene expression in differentiated tissues.	Q6P0C9	C3XR49	A0A1L8FAN2	K7FXK1	Q9PWP8

15	GP RAKVAKLNIQ SLSPVKKKKM VPGALGVPAD LAPVDVEFSF PKFSRLRRGL KAEAVKGPVP AAPARRRLQL PRLRVR	11	Q9BXM0	Periaxin	Scaffolding protein that functions as part of a dystroglycan complex in Schwann cells, and as part of EZR and AHNAK-containing complexes in eye lens fiber cells.	F1QQL4	C3YGB2	A0A1L8G1U2	-	-
16	KPRK	12	Q8TDY2	RB1-inducible coiled-coil protein 1	Involved in autophagy. Regulates early events but also late events of autophagosome formation through direct interaction with Atg16L1	E7FFM2	-	A0A1L8FYZ6	K7G9N3	E1C330
17	RKRKG	13	Q8WYA1	Aryl hydrocarbon receptor nuclear translocator-like protein 2	Transcriptional activator which forms a core component of the circadian clock.	Q9I879	C3Y283	Q6P6Z6	K7FUW5	E1C1C5
18	KQEDKN AMDVIVKVIK R	14	Q13609	Deoxyribonuclease gamma	Has DNA hydrolytic activity. Is capable of both single- and double-stranded DNA cleavage, producing DNA [illegible]	Q6DH05	C3Z4K2	A0A1L8EZN6	K7F8E0	Q3Y8N1
19	LRKKT KSKR									
20	RSKPKK KSKKHKDKE	15	P55199	RNA polymerase II elongation factor ELL	Elongation factor component of the super elongation complex (SEC), a complex required to increase the catalytic rate of RNA polymerase II transcription by suppressing transient pausing by the polymerase at multiple sites along the DNA.	Q6PEG4	C3YQ98	A0A1L8HX53	K7GHT8	F1NBA3
21	KLVPGLFKDE MKRRR	16	P35227	Polycomb group RING finger protein 2	Transcriptional repressor. Binds specifically to the DNA sequence 5'-GACTNGACT-3'.	B0UXV8	C3YW14	Q6NU75	K7FHU3	F1NWQ4
22	KEDSKI LKKARKDGFL HETLLDRRAK L	17	Q14331	Protein FRG1	Binds to mRNA in a sequence-independent manner.	Q566W5	C3XXG6	A0A1L8HU14	-	E1BY86
23	KKKSKDKK RKR									
24	RKAEHW KHKKEESGRE RKKNW	18	Q96AQ6	Pre-B-cell leukemia transcription factor-interacting protein 1	Regulator of pre-B-cell leukemia transcription factors (BPXs) function. Inhibits the binding of PBX1-HOX complex to DNA and blocks the transcriptional activity of E2A-PBX1.	A5WW29	-	A0A1L8FDD5	-	A0A1D5P2Y6
25	KALKK RSGKKDKHSQ SPRAAGPREG									
26	RRTDPECT APIKKQKKR	19	Q8N1G2	Cap-specific mRNA (nucleoside-2'-O-)-methyltransferase 1	S-adenosyl-L-methionine-dependent methyltransferase that mediates mRNA cap1 2'-O-ribose methylation to the 5'-cap structure of mRNAs.	Q803R5	C3Y0P7	Q6GQ76	-	F1NAT2
27	RKKIS NFT	20	Q9H079	KATNB1-like protein 1	Regulates microtubule-severing activity of KATNAL1 in a concentration-dependent manner in vitro.	Q5XJA1	-	Q5EAV1	K7FQB2	Q5ZIG6
28	RKKVHHPF									
29	RDGFRRK	21	Q8NEY8	Periphilin-1	Component of the HUSH complex, a multiprotein complex that mediates epigenetic repression.	A1A5V1	-	Q6DJM5	-	A0A1D5NZJ2
30	KLVTKL QNSERKKRGA RR	22	O60356	Nuclear protein 1	Chromatin-binding protein that converts stress signals into a program of gene expression that empowers cells with resistance to the stress induced by a change in their microenvironment.	E9QCY4	-	A0A1L8H8H5	-	-
31	IKKKQKH	23	P55209	Nucleosome assembly protein 1-like 1	May be involved in modulating chromatin formation and contribute to regulation of cell proliferation.	A0A0R4IDK6	C3Y698	A0A1L8GYL4	K7FK99	E1BZS2
32	KKRKGR	24	Q9H147	Deoxynucleotidyltransferase terminal-interacting protein 1	Increases DNTT terminal deoxynucleotidyltransferase activity (in vitro)	F1Q8X8	C3YCS8	Q6IP16	K7GBG4	-
33	KKQR	25	Q9UFW8	CGG triplet repeat-binding protein 1	Binds to nonmethylated 5'-d(CGG)(n)-3' trinucleotide repeats in the FMR1 promoter. May play a role in regulating FMR1 promoter.	-	-	-	-	A0A1D5NUG1
34	KRRKKE EKR	26	Q9H6E4	Coiled-coil domain-containing protein 134	In extracellular secreted form, promotes proliferation and activation of CD8+ T cells, suggesting a cytokine-like function	A0A0G2KTT4	C3YZU0	A0A1L8GNP2	K7GB64	E1BVM7
35	RKRR	27	Q96EU6	Ribosomal RNA processing protein 36 homolog	Involved in the early processing steps of the pre-rRNA in the maturation pathway leading to the 18S rRNA.	Q1L876	C3ZKP2	A0A1L8G0R1	-	R4GFB9
36	KKRRK	28	O95935	T-box transcription factor TBX18	Acts as transcriptional repressor involved in developmental processes of a variety of tissues and organs, including the heart and coronary vessels, the ureter and the vertebral column. Required for embryonic development of the sino atrial node (SAN) head area.	Q8JFF9	C3XV96	A0A1L8G3B1	K7G3Q5	Q68SB0
37	KRRR	29	Q14153	Protein FAM53B	Acts as a regulator of Wnt signaling pathway by regulating beta-catenin (CTNNB1) nuclear localization.	F1QN48	-	-	-	E1C7N4
38	APK KMKTS	30	Q9NS26	Sperm protein associated with the nucleus on the X chromosome A	spermatogenesis	-	-	-	-	-
39	RKRYGYRL	31	O95478	Ribosome biogenesis protein NSA2 homolog	Involved in the biogenesis of the 60S ribosomal subunit.	F1QDL2	C3Y211	K7G2C3	Q6GP86	Q5ZMH9
40	RKSSHHRKAG ESHRRRAG	32	Q8TC56	Protein FAM71B	May be involved in RNA biogenesis.	-	-	-	-	-

41	RRRRAS APISQWSSSR RSR	33	Q8N699	Myc target protein 1	May regulate certain MYC target genes, MYC seems to be a direct upstream transcriptional activator.	E7FG94	-	A0A1L8G7Z9	K7FBA2	-
42	RRRFK	34	P55786	Puromycin-sensitive aminopeptidase	Aminopeptidase with broad substrate specificity for several peptides. Involved in proteolytic events essential for cell growth and viability.	E7F2N2	C3YCZ6	A0A1L8I1H6	K7FNK2	A0A1D5PAZ7
43	RQKRQARESN SDRVSASKRR SSPSKDGR	35	P61328	Fibroblast growth factor 12	Involved in nervous system development and function. Involved in the positive regulation of voltage-gated sodium channel activity. Promotes neuronal excitability by elevating the voltage dependence of neuronal sodium channel SCN8A fast inactivation.	A0AUQ1	-	A0A1L8F810	K7FYZ7	Q9IAI5
44	KKKKEK	36	Q8NI27	THO complex subunit 2	Required for efficient export of polyadenylated RNA and spliced mRNA.	F1QXF4	C3YRS5	A0A1L8F2J0	K7GAE7	A0A1D5PRL3
45	QQKKQ	37	Q76L83	Putative Polycomb group protein ASXL2	Putative Polycomb group (PcG) protein. PcG proteins act by forming multiprotein complexes, which are required to maintain the transcriptionally repressive state of homeotic genes throughout development. PcG proteins are not required to initiate repression, but to maintain it during later stages of development.	E7FG88	-	-	-	E1BTW9
46	RRKRRRRR	38	Q9BQK8	Phosphatidate phosphatase LPIN3	Regulates fatty acid metabolism. Magnesium-dependent phosphatidate phosphatase enzyme which catalyzes the conversion of phosphatidic acid to diacylglycerol during triglyceride, phosphatidylcholine and phosphatidylethanolamine biosynthesis	F1RAK9	C3YA84	A0A1L8FYL5	K7FWE2	A0A1D5PV53
47	LKKRAR	39	P57772	Selenocysteine-specific elongation factor	Translation factor necessary for the incorporation of selenocysteine into proteins. It probably replaces EF-Tu for the insertion of selenocysteine directed by the UGA codon. SelB binds GTP and GDP.	Q32LX1	C3YLP3	Q6GPS7	-	A0A1D5PXD0
48	KKRRRRARK	40	Q9UMS6	Synaptopodin-2	Has an actin-binding and actin-bundling activity. Can induce the formation of F-actin networks in an isoform-specific manner	E9QFY4	C3Z5E7	A0A1L8FK03	K7FHA8	F1NH40
49	KRK R	41	P49918	Cyclin-dependent kinase inhibitor 1C	Potent tight-binding inhibitor of several G1 cyclin/CDK complexes (cyclin E-CDK2, cyclin D2-CDK4, and cyclin A-CDK2) and, to lesser extent, of the mitotic cyclin B-CDC2.	Q6IQT7	-	Q91603	K7FV40	Q8JIV2
50	RA RARARARA	42	Q9UH62	Armadillo repeat-containing X-linked protein 3	Regulates mitochondrial aggregation and transport in axons in living neurons.	A0A0R4IWE3	C3ZBB8	A0A1L8GZ89	K7FUU6	E1C223
51	KKYGPK	43	Q16527	Cysteine and glycine-rich protein 2	Drastically down-regulated in response to PDGF-BB or cell injury, that promote smooth muscle cell proliferation and dedifferentiation. Seems to play a role in the development of the embryonic vascular system.	Q6P2T6	C3ZK45	A0A1L8GUD0	K7G1K8	A0A1D5NYM7
52	RRRRHRNR	44	P61579	Endogenous retrovirus group K member 25 Rec protein	Retroviral replication requires the nuclear export and translation of unspliced, singly-spliced and multiply-spliced derivatives of the initial genomic transcript.	-	-	-	-	-
53	KRS RRKRRR	45	Q6NSI3	Protein FAM53A	May play an important role in neural development; the dorsomedial roof of the third ventricle.	F1QN48	-	A0A1L8HT13	-	Q5ZKN5
54	KRRKR	46	Q14693	Phosphatidate phosphatase LPIN1	Plays important roles in controlling the metabolism of fatty acids at different levels.	X1WH54	C3YA84	A0A1L8G5J3	K7FWE2	A0A1D5P4T3
55	IKKKRPPV	47	Q8IVN3	Musculoskeletal embryonic nuclear protein 1	May be involved in the development and regeneration of the musculoskeletal system.	Q7ZVB7	C3YAD5	Q7T0M7	K7GIR2	A0A1D5PW18
56	VRKKR KP	48	P49321	Nuclear autoantigenic sperm protein	Required for DNA replication, normal cell cycle progression and cell proliferation. Forms a cytoplasmic complex with HSP90 and H1 linker histones and stimulates HSP90 ATPase activity.	F1QNE0	C3YIL0	-	Q2VPH0	-
57	RQRRSQNKFLRRQRKPQRK	49	Q6NW34	Nucleolus and neural progenitor protein	May play a role in cortex development as part of the Notch signaling pathway.	Q567G6	C3XS37	A0A1L8HH29	K7F2R4	-
58	KRLVPQ KQASVAKKKK	50	Q86SE8	Nucleoplasmin-2	Core histones chaperone involved in chromatin reprogramming, specially during fertilization and early embryonic development. Probably involved in sperm DNA decondensation during fertilization.	Q5BLA5	-	Q6GQG6	K7FKB9	P16039
59	RRQL VHFAWEHFRP RCK	51	Q9NZT2	Opioid growth factor receptor	Receptor for opioid growth factor (OGF), also known as Met-enkephalin. Seems to be involved in growth regulation.	E7EYZ3	C3ZI17	A0A1L8G3Q4	-	F1NEX4

60	RRR SKFANLGRIF KPWKWRKKK	52	Q9C0D0	Phosphatase and actin regulator 1	Binds actin monomers (G actin) and plays a role in the reorganization of the actin cytoskeleton and in formation of actin stress fibers. Plays a role in cell motility. Plays a role in the formation of tubules by endothelial cells. Regulates PPP1CA activity. Required for normal cell survival.	F1R9D7	-	A0A1L8FY03	-	F1NWB1
61	RRRKRR	53	Q6MZT1	Regulator of G-protein signaling 7-binding protein	Regulator of G protein-coupled receptor (GPCR) signaling. Regulatory subunit of the R7-Gbeta5 complexes that acts by controlling the subcellular location of the R7-Gbeta5 complexes.	B2GRF5	-	A0A1L8I255	K7FKC4	R4GI70
62	KKIKKKKKK KHR	54	Q5VWQ0	Lysine-specific demethylase 9	Histone demethylase that specifically demethylates dimethylated 'Lys-20' of histone H4 (H4K20me2), thereby modulating chromosome architecture.	A0A0G2KVT3	-	A0A1L8HEY8	-	A0A1D5PEL9
63	VARRR	55	Q7Z7C7	Stimulated by retinoic acid gene 8 protein homolog	Meiosis-inducer required for the transition into meiosis for both female and male germ cells. In female germ cells, required for premeiotic DNA replication and subsequent events in meiotic prophase.	-	C3Y531	A0A1L8GQI6	-	E1C4S4
64	KKKRK	56	O75683	Surfeit locus protein 6	Binds to both DNA and RNA in vitro, with a stronger binding capacity for RNA. May represent a nucleolar [illegible]	Q7T3A6	-	Q0IH42	-	Q5ZHN7
65	RKKRK									
66	KLKKKKMA	57	Q05952	Nuclear transition protein 2	Plays a key role in the replacement of histones to protamine in the elongating spermatids of mammals. In condensing spermatids, loaded onto the nucleosomes, where it promotes the recruitment and processing of protamines, which are responsible for histone eviction.	XP_021324227.1	-	-	-	-
67	KKWK	58	Q15431	Synaptonemal complex protein 1	Major component of the transverse filaments of synaptonemal complexes (SCS), formed between homologous chromosomes during meiotic prophase. [illegible]	F1QYS1	-	-	-	-
68	LE EV									
69	KK KRK									
70	KRK R	59	Q8IZU3	Synaptonemal complex protein 3	Component of the synaptonemal complexes (SCS), formed between homologous chromosomes during meiotic prophase. Required for centromere pairing during meiosis in male germ cells	Q1RLP7	C3ZM03	A0A1L8GIK4	-	E1BWM3
71	RKRLAG	60	Q9P016	Thymocyte nuclear protein 1	Specifically binds 5-hydroxymethylcytosine (5hmC), suggesting that it acts as a specific reader of 5hmC.	F1QDP5	C3XST2	A0A1L8FL07	K7GB28	Q90679
72	KYKEGK	61	O60688	Protein yippee-like 1	May play a role in epithelioid conversion of fibroblasts.	Q6AXK6	C3YVP3	Q6DDQ0	K7F5P3	F1NA62
73	RAKWR RHQRA	62	P0C7M4	Rhox homeobox family member 2B	Transcription factor maybe involved in reproductive processes. Modulates expression of target genes encoding proteins involved in processes relevant to spermatogenesis.	Q6P295	-	-	-	-
74	VHFKISGE KRPSADPGKK AKNPKKKKKKDP	63	Q96NM4	TOX high mobility group box family member 2	Putative transcriptional activator involved in the hypothalamo-pituitary-gonadal system.	B0V356	-	A0A1L8FZ33	K7FWE5	A0A1D5PSC7
75	RPKGRQRK	64	O15234	Protein CASC3	Core component of the splicing-dependent multiprotein exon junction complex (EJC) deposited at splice [illegible]	Q1ECZ4	C3XUF7	A0JMU8	-	A0A1D5PM89
76	KPRRIRK PR									
77	A RVCRRK	65	Q7Z2W4	Zinc finger CCCH-type antiviral protein 1	Antiviral protein which inhibits the replication of viruses by recruiting the cellular RNA degradation machineries to degrade the viral mRNAs.	A0A0R4IH74	C3YKR0	A0A1L8G4F7	K7FBX9	A0A1D5PEY4
78	KKR IRPLETTQQI RKRHC	66	Q9BX63	Fanconi anemia group J protein	DNA-dependent ATPase and 5' to 3' DNA helicase required for the maintenance of chromosomal stability.	B7ZDD5	C3YXX3	A0A1L8HG08	K7G641	Q3YK19
79	RKR RRR	67	Q8N693	Homeobox protein ESX1	May coordinately regulate cell cycle progression and transcription during spermatogenesis.	Q6P984	-	-	-	-
80	KKRK	68	Q14320	Protein FAM50A	May be a DNA-binding protein or transcriptional factor.	Q568K9	C3ZVJ9	Q2VPH1	-	-
81	PKVKRSRKA	69	P41223	Protein BUD31 homolog	Involved in the pre-mRNA splicing process	A0A0R4I9A3	C3Y9A1	A0A1L8EXA3	K7FWH2	E1BV47
82	KKKR KR	70	Q7LFL8	CXXC-type zinc finger protein 5	May indirectly participate in activation of the NF-kappa-B and MAPK pathways.	E7FC24	-	A0A1L8HV25	-	F1NCW6
83	KAKRVSRN KSEKKRR	71	O15516	Circadian locomoter output cycles protein kaput	Transcriptional activator which forms a core component of the circadian clock.	B3DH92	C3XWF6	Q9I9Q6	K7FXZ9	Q8QGQ6
84	KRKHV VK	72	Q8N9N8	Probable RNA-binding protein EIF1AD	Plays a role into cellular response to oxidative stress. Decreases cell proliferation.	Q7SY07	C3XYZ9	A0A1L8GK34	K7F4B4	Q6K1L7
85	KYRKN IWIKR									
86	KRKKRRHR	73	Q9NZR4	Visual system homeobox 1	Binds to the 37-bp core of the locus control region (LCR) of the red/green visual pigment gene cluster. May regulate the activity of the LCR and the cone opsin genes at earlier stages of development.	O42250	C3XZK7	Q0P031	-	Q9IAL2

87	RRR	74	Q9BSI4	TERF1-interacting nuclear factor 2	Component of the shelterin complex (telosome) that is involved in the regulation of telomere length and protection.	F1R9M2	-	-	-	-
88	KT KRK	75	Q8IX15	Homeobox and leucine zipper protein Homez	May function as a transcriptional regulator.	A5D8R5	C3Z1B2	A0A1L8G021	K7FK68	A0A1D5PYW2
89	RKRR K	76	Q00653	Nuclear factor NF-kappa-B p100 subunit	NF-kappa-B is a pleiotropic transcription factor present in almost all cell types and is the endpoint of a series of signal transduction events that are initiated by a vast array of stimuli related to many biological processes such as inflammation, immunity, differentiation, cell growth, tumorigenesis and apoptosis.	Q0II00	-	P51510	K7FNJ5	P98152
90	R KKRGR	77	Q96JN0	Ligand-dependent corepressor	May act as transcription activator that binds DNA elements with the sequence 5'-CCCTATCGATCGATCTCTACCT-3'	A0PJQ8	-	A0A1L8FEN4	-	-
91	KRRKK RQKLKEKK	78	Q9H875	PRKR-interacting protein 1	Binds double-stranded RNA. Inhibits EIF2AK2 kinase activity	B2GRY3	C3Z487	A0A1L8HDZ0	-	Q5ZKU0
92	ELRLKEELLK GIYA	79	Q9UHL0	ATP-dependent RNA helicase DDX25	ATP-dependent RNA helicase. Required for mRNA export and translation regulation during spermatid development	Q8JGR1	C3YRH2	A0A1L8FPD8	-	A0A1D5NVY9
93	AR RRR	80	Q9NUL7	Probable ATP-dependent RNA helicase DDX28	Plays an essential role in facilitating the proper assembly of the mitochondrial large ribosomal subunit and its helicase activity is essential for this function	Q08BL1	C3XRL7	A0A1L8GKB9	-	-
94	RRAKWR RHQR	81	Q9BQY4	Rhox homeobox family member 2	Transcription factor maybe involved in reproductive processes. Modulates expression of target genes encoding proteins involved in processes relevant to spermatogenesis.	Q6P295	-	-	-	-
95	R KHVKAHSAKE QQVRKK	82	Q8NBF1	Zinc finger protein GLIS1	Acts as both a repressor and activator of transcription. Binds to the consensus sequence 5'-GACCACCCAC-3'	F1QRD5	C3XY75	A0A1L8HY67	-	F1ND41
96	KRKR	83	Q04206	Transcription factor p65	NF-kappa-B is a pleiotropic transcription factor present in almost all cell types and is the endpoint of a series of signal transduction events that are initiated by a vast array of stimuli related to many biological processes such as inflammation, immunity, differentiation, cell growth, tumorigenesis and apoptosis.	Q6DGE6	C3XR39	A0A1L8GK02	K7FNJ5	P98152
97	RLKRIWNWRA CEAAKKTKAD DR	84	O60563	Cyclin-T1	Regulatory subunit of the cyclin-dependent kinase pair (CDK9/cyclin-T1) complex, also called positive transcription elongation factor B (P-TEFb), which is proposed to facilitate the transition from abortive to productive elongation by phosphorylating the CTD (carboxy-terminal domain) of the large subunit of RNA polymerase II (RNA Pol II).	Q803Q8	-	A0A1L8HHN4	K7F433	F1NIJ9
98	KRRKKK	85	Q01201	Transcription factor RelB	NF-kappa-B is a pleiotropic transcription factor which is present in almost all cell types and is involved in many biological processed such as inflammation, immunity, differentiation, cell growth, tumorigenesis and apoptosis.	Q0II00	C3XR39	P51510	K7FNJ5	Q04861
99	MPPKGKSGSG KAGKGGAASG SDSADKK	86	Q9Y237	Peptidyl-prolyl cis-trans isomerase NIMA-interacting 4	Involved as a ribosomal RNA processing factor in ribosome biogenesis. Binds to tightly bent AT-rich stretches of double-stranded DNA	Q503Y7	-	-	-	-
100	RRRR	87	Q9UGN5	Poly [ADP-ribose] polymerase 2	Involved in the base excision repair (BER) pathway, by catalyzing the poly(ADP-ribosyl)ation of a limited [illegible]	Q7ZVB0	C3Z2P3	Q5M9A2	-	E1BSI0
101	KKTR R									
102	K AKKQK	88	Q04864	Proto-oncogene c-Rel	Proto-oncogene that may play a role in differentiation and lymphopoiesis.	Q6K199	-	Q91833	K7FNJ5	-
103	KKLQEQ EK	89	O75391	Sperm-associated antigen 7	Nucleic acid binding	Q7SYJ9	C3XPX9	A0A1L8EK33	-	-
104	RRGEEWDPQ KAEEKRKLK									
105	KKGR RNRFK	90	P20823	Hepatocyte nuclear factor 1-alpha	Transcriptional activator that regulates the tissue specific expression of multiple genes, especially in pancreatic islet cells and in liver. Required for the expression of several liver specific genes. Binds to the inverted palindrome 5'-GTTAATNATTAAC-3'.	Q8UVH4	-	-	-	-
106	RK KVKYRK	91	O94983	Calmodulin-binding transcription activator 2	Transcription activator. May act as tumor suppressor.	UPI0004F4AA16	C3ZIH0	A0A1L8H472	-	A0A1D5PBI0
107	KKQESNHLQI HLCRKK	92	Q9NZ71	Regulator of telomere	ATP-dependent DNA helicase implicated in telomere-	P0C928	C3YXX3	A0A1L8ET84	K7G641	F1NE49

108	RGGRKK			elongation helicase 1	length regulation, DNA repair and the maintenance of				
109	RKKVKYRK	93	Q9Y6Y1	Calmodulin-binding transcription activator 1	Transcriptional activator. May act as a tumor suppressor.	UPI00038359AD	C3ZIH0	A0A1L8H472	-	A0A1D5PBI0
110	KIKEL YRRR	94	O75925	E3 SUMO-protein ligase PIAS1	Functions as an E3-type small ubiquitin-like modifier (SUMO) ligase, stabilizing the interaction between	X1WDM3	C3Z9X9	Q5XHH3	K7FKR8	A0A1D5P1W4
111	KKP TWVCPVCDKK									
112	KRKADGY NQPDSKRR	95	O60506	Heterogeneous nuclear ribonucleoprotein Q	Heterogenous nuclear ribonucleoprotein (hnRNP) implicated in mRNA processing mechanisms. Component of the CRD-mediated complex that promotes MYC mRNA stability.	Q7SZC9	C3ZLB6	Q6NS22	-	A0A1D5PJG4
113	KD KKKK	96	Q08211	ATP-dependent RNA helicase A	Multifunctional ATP-dependent nucleic acid helicase that unwinds DNA and RNA in a 3' to 5' direction and that plays important roles in many processes, such as DNA replication, transcriptional activation, post-transcriptional RNA regulation, mRNA translation and RNA-mediated gene silencing	E7F525	C3Y332	A0A1L8I2C1	K7FFE8	E1C388
114	KR KQLHQLLPNH VLQKKKKH	97	P51003	Poly(A) polymerase alpha	Polymerase that creates the 3'-poly(A) tail of mRNA's. Also required for the endoribonucleolytic cleavage reaction at some polyadenylation sites. May acquire specificity through interaction with a cleavage and polyadenylation specificity factor (CPSF) at its C-terminus.	Q5SNY2	C3Z3M0	A0A1L8F081	-	A0A1D5P5T8
115	KKRVHR	98	O75840	Krueppel-like factor 7	Transcriptional activator. Binds in vitro to the CACCC motif of the beta-globin promoter and to the SP1 recognition sequence.	Q502L8	C3ZT77	A0A1L8EX25	K7FC91	F1P0T6
116	KRKEVKR	99	Q14872	Metal regulatory transcription factor 1	Activates the metallothionein I promoter. Binds to the metal responsive element (MRE).	A0A0G2KFP7	C3YX65	Q0VGW3	K7FDS6	Q5ZLE7
117	KRKNAKMI SDIEKKRQRM	100	Q71F23	Centromere protein U	Component of the CENPA-NAC (nucleosome-associated) complex, a complex that plays a central role	E7FCV0	-	A0A1L8HU11	-	Q2Z1W2
118	RRRPR PHRSEGARRS KNT									
119	KRPR	101	O15151	Protein Mdm4	Inhibits p53/TP53- and TP73/p73-mediated cell cycle arrest and apoptosis by binding its transcriptional activation domain. Inhibits degradation of MDM2.	F1QD25	C3Y7L6	Q7ZYI3	K7GDW4	F1NGX6
120	KKFGAQNV ARRIEFRKK	102	Q9NYB0	Telomeric repeat-binding factor 2-interacting protein 1	Acts both as a regulator of telomere function and as a transcription regulator.	A0A0R4IRZ2	C3XRX4	A0A1L8ERQ2	K7FQB6	Q7T0L4
121	AVSVI TISSDTDEEE TSQRHSLREC KGSLDCEACQ STLNIDRMCS LSSPDST	103	Q9H422	Homeodomain-interacting protein kinase 3	Serine/threonine-protein kinase involved in transcription regulation, apoptosis and steroidogenic gene expression.	F6NWN5	C3Y5H6	A0A1L8GJ10	K7G117	E1BUN6
122	KRPR	104	Q06587	E3 ubiquitin-protein ligase RING1	Constitutes one of the E3 ubiquitin-protein ligases that mediate monoubiquitination of 'Lys-119' of histone H2A, thereby playing a central role in histone code and gene regulation.	A0A0H2UKC6	C3ZPC6	A0A1L8F8A8	K7G2D5	A0A1D5P1F4
123	KKTSGL QQKNVE	105	Q9UBU8	Mortality factor 4-like protein 1	Component of the NuA4 histone acetyltransferase (HAT) complex which is involved in transcriptional activation of select genes principally by acetylation of nucleosomal histones H4 and H2A.	A8E7S8	C3ZKT2	Q66J61	K7GG49	A0A1L1RTQ0
124	KKERKRL KEEKQKRKKE	106	Q8TAE8	Growth arrest and DNA damage-inducible proteins-interacting protein 1	Acts as a negative regulator of G1 to S cell cycle phase progression by inhibiting cyclin-dependent kinases.	UPI0002026CE4	C3YM53	-	-	-
125	RCVLAACSTY FKKLFKK	107	O43829	Zinc finger and BTB domain-containing protein 14	Transcriptional activator of the dopamine transporter (DAT), binding it's promoter at the consensus sequence 5'-CCTGCACAGTTCACGGA-3'.	Q7SYJ3	C3ZBA4	A0A1L8F220	K7FD47	Q92010
126	KKKRLR	108	Q5T1R4	Transcription factor HIVEP3	Plays a role of transcription factor; binds to recognition signal sequences (Rss heptamer) for somatic recombination of immunoglobulin and T-cell receptor gene segments; Binds also to the kappa-B motif of gene such as S100A4, involved in cell progression and differentiation.	A0A286Y903	-	Q5MYB9	K7GD73	F1P1B9
127	KTPR K	109	Q02241	Kinesin-like protein KIF23	Component of the centralspindlin complex that serves as a microtubule-dependent and Rho-mediated signaling required for the myosin contractile ring formation during the cell cycle cytokinesis.	A0A286Y967	C3Z0T9	A0A1L8H194	-	A0A1D5PCT6
128	SKP KKK	110	Q9NZJ0	Denticleless protein homolog	Substrate-specific adapter of a DCX (DDB1-CUL4-X-box) E3 ubiquitin-protein ligase complex required for cell cycle control, DNA damage response and translesion DNA synthesis.	Q5RHI5	C3Y089	Q6GPU3	K7FX52	Q5ZJW8
129	KK SFSTLFLETV KRK	111	Q03188	Centromere protein C	Component of the CENPA-NAC (nucleosome-	UPI0004572D87	-	C8YR60	K7F4L0	O57392

130	KKSSTRK DKEESKKKR				associated) complex, a complex that plays a central role in assembly of kinetochore proteins, mitotic progression and chromosome segregation.				
131	RKS TKKTNQSSKN IRKK								
132	KRKAKENIGKV NKKSNKKR								
133	RQ RKRHK	112	Q00987	E3 ubiquitin-protein ligase Mdm2	E3 ubiquitin-protein ligase that mediates ubiquitination of p53/TP53, leading to its degradation by the [illegible]	A0A0R4ISQ1	C3Y7L6	A0A1L8GYI7	K7GDW4	F1NGX6
134	KKLKK RNK									
135	RKRPRR	113	Q96EB6	NAD-dependent protein deacetylase sirtuin-1	NAD-dependent protein deacetylase that links transcriptional regulation directly to intracellular [illegible]	A1L2B7	C3Z3T3	Q5U250	-	A0A1P8D6T9
136	KRKKRK									
137	KRKAKA	114	Q15025	TNFAIP3-interacting protein 1	Inhibits NF-kappa-B activation and TNF-induced NF-kappa-B-dependent gene expression by regulating A20/TNFAIP3-mediated deubiquitination of IKBKG; proposed to link A20/TNFAIP3 to ubiquitinated IKBKG.	A1L1N2	C3Z5J9	A0A1L8GWT2	K7G737	A0A1D5PHA8
138	RRKRSKSE DMDNVQSKRR R	115	Q6VMQ6	Activating transcription factor 7-interacting protein 1	Recruiter that couples transcriptional factors to general transcription apparatus and thereby modulates transcription regulation and chromatin formation.	A0JME2	-	A0A1L8GNC0	K7F6D4	-
139	KKRK PK	116	Q13769	THO complex subunit 5 homolog	Acts as component of the THO subcomplex of the TREX complex which is thought to couple mRNA transcription, processing and nuclear export, and which specifically associates with spliced mRNA and not with unspliced pre-mRNA.	Q6NY52	C3Z469	A0A1L8HZU7	K7FFP2	Q5ZJK1
140	KLLKDLPEL	117	Q53EL6	Programmed cell death protein 4	Inhibits translation initiation and cap-dependent translation.	Q7SYL0	C3Y3Z6	Q7T0M4	K7FSZ2	Q98TX3
141	KAK RRLRK									
142	RRKH F	118	Q8N9U0	Tandem C2 domains nuclear protein	Many C2 domains are Ca2+-dependent membrane-targeting modules that bind a wide variety of substances including bind phospholipids, inositol polyphosphates, and intracellular proteins. Most C2 domain proteins are either signal transduction enzymes that contain a single C2 domain, such as protein kinase C, or membrane trafficking proteins which contain at least two C2 domains, such as synaptotagmin 1.	UPI0004F450CE	-	A0A1L8F9H6	K7G6Y4	A0A1D5NW06
143	K IK	119	Q16644	MAP kinase-activated protein kinase 3	Stress-activated serine/threonine-protein kinase involved in cytokines production, endocytosis, cell [illegible]	Q6DHN7	C3YQW4	Q6IRB4	K7F3M5	E1BT35
144	KRRKK									
145	RRR TVQEARALLR CRRAG	120	Q96S44	EKC/KEOPS complex subunit TP53RK	Component of the EKC/KEOPS complex that is required for the formation of a threonylcarbamoyl group on adenosine at position 37 (t6A37) in tRNAs that read codons beginning with adenine	F1QB90	C3ZAV8	Q4V7S0	-	-
146	KKQALKEKE LGNDAYKKK	121	P31948	Stress-induced-phosphoprotein 1	Acts as a co-chaperone for HSP90AA1	Q5RKM3	C3Y009	Q7ZWU1	-	-
147	RRLRIWR	122	Q7KZF4	Staphylococcal nuclease domain-containing protein 1	Endonuclease that mediates miRNA decay of both protein-free and AGO2-loaded miRNAs	Q5RGK8	C3Y7K6	Q7ZY98	-	-
148	KKL RP									
149	KKER RVGTPQSTKK KKESRR	123	P54274	Telomeric repeat-binding factor 1	Binds the telomeric double-stranded 5'-TTAGGG-3' repeat and negatively regulates telomere length.	Q6NW47	C3ZQ21	Q1WM12	-	F1NSI5
150	K RRK	124	Q01658	Protein Dr1	The association of the DR1/DRAP1 heterodimer with TBP results in a functional repression of both activated and basal transcription of class II genes. This interaction precludes the formation of a transcription-competent complex by inhibiting the association of TFIIA and/or TFIIB with TBP.	Q08BR1	C3ZFY4	A0A1L8GMS5	-	Q5ZMV3
151	RKGKGQ IEKRKLREKR R	125	Q96IZ0	PRKC apoptosis WT1 regulator protein	Pro-apoptopic protein capable of selectively inducing apoptosis in cancer cells, sensitizing the cells to diverse apoptotic stimuli and causing regression of tumors in animal models.	Q5XJ95	-	Q66JA2	-	F1NA74
152	KRRQTSMTDF YHSKRR	126	P38936	Cyclin-dependent kinase inhibitor 1	May be involved in p53/TP53 mediated inhibition of cellular proliferation in response to DNA damage.	Q5RGY9	C3YUU6	Q91646	K7FV40	Q8JIV2
153	KKNAEKEKQ QRNQKKKK	127	Q9BXJ9	N-alpha-acetyltransferase 15, NatA auxiliary subunit	Auxillary subunit of the N-terminal acetyltransferase A (NatA) complex which displays alpha (N-terminal) acetyltransferase activity.	F1QE25	C3XX70	A0A1L8HUJ1	K7FHQ9	F1NVR6
154	KRSVVSFDKVKE PRKSR	128	Q15424	Scaffold attachment factor B1	Binds to scaffold/matrix attachment region (S/MAR) DNA and forms a molecular assembly point to allow the formation of a 'transcriptosomal' complex (consisting of SR proteins and RNA polymerase II) coupling transcription and RNA processing	Q803X6	C3Z0S4	A0A1L8HWX4	K7FD64	H9L022
155	KKRK	129	Q04724	Transducin-like enhancer protein 1	Transcriptional corepressor that binds to a number of transcription factors.	O13168	C3ZWE3	B1H1Y7	-	A0A1D5PGR2

156	RASIER KRQRALMLRQ ARLAARP	130	P23025	DNA repair protein complementing XP-A cells	Involved in DNA excision repair. Initiates repair by binding to damaged sites with various affinities, depending on the photoproduct and the transcriptional state of the region. Required for UV-induced CHEK1 phosphorylation and the recruitment of CEP164 to cyclobutane pyrimidine dimmers (CPD), sites of DNA damage after UV irradiation.	Q7SY02	C3YQX9	P27088	-	R4GJS9
157	KKAKLR	131	Q99661	Kinesin-like protein KIF2C	In complex with KIF18B, constitutes the major microtubule plus-end depolymerizing activity in mitotic cells	F1RAK1	C3YLZ9	Q91636	K7F465	A0A1D5PBU5
158	KRKR	132	Q9H814	Phosphorylated adapter RNA export protein	A phosphoprotein adapter involved in the XPO1-mediated U snRNA export from the nucleus. Bridge	Q68EH5	C3YBG9	Q5U568	-	Q5ZLY0
159	KRK R									
160	KK RSR	133	Q8IXJ9	Putative Polycomb group protein ASXL1	Probable Polycomb group (PcG) protein involved in transcriptional regulation mediated by ligand-bound nuclear hormone receptors, such as retinoic acid receptors (RARs) and peroxisome proliferator-activated receptor gamma (PPARG).	F1Q5H5	C3YBN3	A0A1L8ERK1	-	A0A1D5PLQ0
161	GGKPCSQHI ISVTGFVDS	134	Q6ZW49	PAX-interacting protein 1	Involved in DNA damage response and in transcriptional regulation through histone methyltransferase (HMT) complexes.	A0A0R4ID17	C3YDM2	A0A1L8FQI7	-	A0A1D5NZ11
162	KQRA	135	Q04725	Transducin-like enhancer protein 2	Transcriptional corepressor that binds to a number of transcription factors. Inhibits the transcriptional activation mediated by CTNNB1 and TCF family members in Wnt signaling. The effects of full-length TLE family members may be modulated by association with dominant-negative AES	F1R8R4	C3ZWE3	A0A1L8HPC6	-	A0A1D5PVL6
163	R LRRAARPHRR LGPTGKEVHA LKRLR	136	Q6NXT1	Ankyrin repeat domain-containing protein 54	Plays an important role in regulating intracellular signaling events associated with erythroid terminal differentiation.	Q6DGX3	C4A083	A0A1L8GNU4	-	F1NBL4
164	RLKAK	137	Q99697	Pituitary homeobox 2	Controls cell proliferation in a tissue-specific manner and is involved in morphogenesis. During embryonic development, exerts a role in the expansion of muscle progenitors.	Q9W5Z2	C3Z639	Q9PWR3	K7F389	O93385
165	RLKSK	138	P78337	Pituitary homeobox 1	Sequence-specific transcription factor that binds gene promoters and activates their transcription.	B2LT56	C3Z639	Q6GNM5	K7F389	A0A1D5P373
166	RRARGGAV SARYVLPDEA ARAR	139	Q16690	Dual specificity protein phosphatase 5	Dual specificity protein phosphatase; active with phosphotyrosine, phosphoserine and phosphothreonine residues. The highest relative activity is toward ERK1.	Q6DBR5	C3Z8W6	A0A1L8HWG3	-	Q9PW71
167	RKVGRSLQRG CRALR	140	Q15628	Tumor necrosis factor receptor type 1-associated DEATH domain protein	The nuclear form acts as a tumor suppressor by preventing ubiquitination and degradation of isoform p19ARF/ARF of CDKN2A by TRIP12: acts by interacting with TRIP12, leading to disrupt interaction between TRIP12 and isoform p19ARF/ARF of CDKN2A	B2GNQ4	-	A0A1L8GKL2	K7F8S3	-
168	RL KAK	141	Q9Y2V3	Retinal homeobox protein Rx	Plays a critical role in eye formation by regulating the initial specification of retinal cells and/or their subsequent proliferation. Binds to the photoreceptor conserved element-I (PCE-1/Ret 1) in the photoreceptor cell-specific arrestin promoter.	O42358	C3ZEF8	Q0ZLI0	K7FM49	Q9PVY0
169	RKRK	142	Q9UKZ4	Teneurin-1	Teneurin C-terminal-associated peptide: Plays a role in the regulation of neuroplasticity in the limbic system. Mediates a rapid reorganization of actin- and tubulin-based cytoskeleton elements with an increase in dendritic arborization and spine density formation of neurons in the hippocampus and amygdala.	A0A286Y8Z9	C3YZB2	A0A1L8HUA7	-	F1NN19
170	RK AKKDDQMLKR RNVS	143	P52292	Importin subunit alpha-1	Functions in nuclear protein import as an adapter protein for nuclear receptor KPNB1.	Q6DI01	C3XTJ8	Q6IRB7	K7FQJ3	Q5ZLF7
171	RKPVTA QERQREREEK RRRRQERAKE REKRRQERER	144	Q13164	Mitogen-activated protein kinase 7	Plays a role in various cellular processes such as proliferation, differentiation and cell survival. The upstream activator of MAPK7 is the MAPK kinase MAP2K5. Upon activation, it translocates to the nucleus and phosphorylates various downstream targets including MEF2C.	Q7ZVK8	C3Z484	Q6DFK6	K7FFZ9	F1N862
172	QLFKRRNV A	145	P52294	Importin subunit alpha-5	Functions in nuclear protein import as an adapter protein for nuclear receptor KPNB1.	A0A0R4I9E5	C3ZM42	Q70PC5	K7GFN3	Q5ZML1

173	KKS ILKKKEQSH	146	Q9HBY8	Serine/threonine-protein kinase Sgk2	Serine/threonine-protein kinase which is involved in the regulation of a wide variety of ion channels, membrane transporters, cell growth, survival and proliferation. Up-regulates Na+ channels: SCNN1A/ENAC, K+ channels: KCNA3/Kv1.3, KCNE1 and KCNQ1, amino acid transporter: SLC6A19, glutamate transporter: SLC1A6/EAAT4, glutamate receptors: GRIA1/GLUR1 and GRIK2/GLUR6, Na+/H+ exchanger: SLC9A3/NHE3, and the Na+/K+ ATPase.	Q08BW4	C3Y291	A0A1L8FZ68	K7G4F8	A0A1D5PWW9
174	KKVK	147	P06748	Nucleophosmin	Involved in diverse cellular processes such as ribosome biogenesis, centrosome duplication, protein chaperoning, histone assembly, cell proliferation, and regulation of tumor suppressors p53/TP53 and ARF.	E7FCU0	-	Q7ZTP6	-	P16039
175	KYLPQ QQKKK	148	Q9UKK3	Poly [ADP-ribose] polymerase 4	NAD+ + (ADP-D-ribosyl)(n)-acceptor = nicotinamide + (ADP-D-ribosyl)(n+1)-acceptor.	UPI0007EE98CA	C3Y4W7	A0A1L8HJ59	-	A0A1D5PL17
176	SAKRPK	149	Q09472	Histone acetyltransferase p300	Functions as histone acetyltransferase and regulates transcription via chromatin remodeling	A0A0R4IVZ7	C3ZUG6	A0A1L8GGS2	-	E1BSS0
177	KK KYHN	150	O43683	Mitotic checkpoint serine/threonine-protein kinase BUB1	Serine/threonine-protein kinase that performs 2 crucial functions during mitosis: it is essential for spindle-assembly checkpoint signaling and for correct chromosome alignment.	F1QNS6	C3XYZ6	Q8JGT8	-	A0A1D5PR30
178	K KKK	151	Q13107	Ubiquitin carboxyl-terminal hydrolase 4	Hydrolase that deubiquitinates target proteins such as the receptor ADORA2A, PDPK1 and TRIM21.	E7EZD6	C3Z9Q5	A0A1L8GHB6	K7G710	Q5ZKD8
179	KKKRR	152	P21675	Transcription initiation factor TFIID subunit 1	Largest component and core scaffold of the TFIID basal transcription factor complex	Q1LYC2	C3Y3A3	A0A1L8F6Z1	-	B5AHE5
180	RRSKL RKPKPVPLRK	153	O75410	Transforming acidic coiled-coil-containing protein 1	Likely involved in the processes that promote cell division prior to the formation of differentiated tissues.	Q6Y8G7	-	A0JMR5	K7FXY6	A0A1D5PMU3
181	KKAKS RLITSGCKVK K									
182	NKGWSGPKPG F	154	Q9NUL3	Double-stranded RNA-binding protein Staufen homolog 2	RNA-binding protein required for the microtubule-dependent transport of neuronal RNA from the cell body to the dendrite.	F1QJT6	C3ZTW2	A0A1L8FT79	K7FV25	A0A1D5PAM6
183	KKLPPLPV VEKPKLFFKK RPK									
184	KRKPQEESERAKSD ESIKEEDKDQ DEKRRR	155	P26358	DNA (cytosine-5)-methyltransferase 1	Methylates CpG residues. Preferentially methylates hemimethylated DNA.	B3DKL7	C3YZQ2	P79922	-	Q92072
185	KRGL SVDSAQEVKR FR	156	Q9Y4A5	Transformation/transcription domain-associated protein	Adapter protein, which is found in various multiprotein chromatin complexes with histone acetyltransferase activity (HAT), which gives a specific tag for epigenetic transcription activation.	A0A0R4ITC5	C3ZR41	A0A1L8EQF0	-	E1C796
186	LGRR R	157	O95831	Apoptosis-inducing factor 1, mitochondrial	Functions both as NADH oxidoreductase and as regulator of apoptosis. In response to apoptotic stimuli, it is released from the mitochondrion intermembrane space into the cytosol and to the nucleus, where it functions as a proapoptotic factor in a caspase-independent pathway.	F1RBU0	C3YZW2	A0A1L8F6P6	K7FF97	Q5ZHL7
187	MANKRMKVKH	158	Q86T24	Transcriptional regulator Kaiso	Transcriptional regulator with bimodal DNA-binding specificity. Binds to methylated CpG dinucleotides in the consensus sequence 5'-CGCG-3' and also binds to the non-methylated consensus sequence 5'-CTGCNA-3' also known as the consensus kaiso binding site (KBS).	Q6PUQ9	C3ZBA4	A0A1L8F758	K7GIX9	F6U492
188	KRK VSSSKPEENK IR	159	Q03933	Heat shock factor protein 2	DNA-binding protein that specifically binds heat shock promoter elements (HSE) and activates transcription. In [illegible]	Q90XA8	C3ZBQ1	Q5EG56	K7G0A2	A0A1D5P919
189	KRKRPL LLNTNGAQKK									
190	RLK AK	160	O75364	Pituitary homeobox 3	Transcriptional regulator which is important for the differentiation and maintenance of meso-diencephalic dopaminergic (mdDA) neurons during development.	B2GRZ8	C3Z639	Q9I8K3	K7GG06	E1C143
191	RREELAQ IRKEEKEKKR RR	161	Q9Y5A7	NEDD8 ultimate buster 1	Specific down-regulator of the NEDD8 conjugation system. Recruits NEDD8, UBD, and their conjugates to the proteasome for degradation. Isoform 1 promotes the degradation of NEDD8 more efficiently than isoform 2	A8WFT9	C3YYI8	A0A1L8FV51	K7FNN9	E1BZ00
192	HLLKRRN VP	162	O00629	Importin subunit alpha-3	Functions in nuclear protein import as an adapter protein for nuclear receptor KPNB1.	Q6DI01	C3XTJ8	Q6IRB7	K7FQJ3	Q5ZLF7

193	K KRKK	163	P23528	Cofilin-1	Binds to F-actin and exhibits pH-sensitive F-actin depolymerizing activity. Regulates actin cytoskeleton dynamics. Important for normal progress through mitosis and normal cytokinesis. Plays a role in the regulation of cell morphology and cytoskeletal organization. Required for the up-regulation of atypical chemokine receptor ACKR2 from endosomal compartment to cell membrane, increasing its efficiency in chemokine uptake and degradation	Q6TH32	C4A075	A0A1L8GCW3	K7F4Y0	P21566
194	RGKRKR	164	P20700	Lamin-B1	Lamins are components of the nuclear lamina, a fibrous layer on the nucleoplasmic side of the inner nuclear membrane, which is thought to provide a framework for the nuclear envelope and may also interact with chromatin.	Q803N8	C3ZXL7	Q8AVZ3	K7FK39	P14731
195	K KKIQKQLEHL KKLRK	165	O15553	Pyrin	Involved in the regulation of innate immunity and the inflammatory response in response to IFNG/IFN-gamma. Organizes autophagic machinery by serving as a platform for the assembly of ULK1, Beclin 1/BECN1, ATG16L1, and ATG8 family members and recognizes specific autophagy targets, thus coordinating target recognition with assembly of the autophagic apparatus and initiation of autophagy. Acts as an autophagy receptor for the degradation of several inflammasome components, including CASP1, NLRP1 and NLRP3, hence preventing excessive IL1B- and IL18-mediated inflammation	Q7ZU70	-	A0A1L8HI93	K7FKU0	A5HUK4
196	KLRK	166	Q2VIQ3	Chromosome-associated kinesin KIF4B	Motor protein that translocates PRC1 to the plus ends of interdigitating spindle microtubules during the metaphase to anaphase transition, an essential step for the formation of an organized central spindle midzone and midbody and for successful cytokinesis.	F8W409	C3Z125	Q91784	K7FYR1	Q90640
197	RK HKK	167	Q8IWS0	PHD finger protein 6	Transcriptional regulator that associates with ribosomal RNA promoters and suppresses ribosomal RNA (rRNA)	Q803W6	C3ZZY1	A0A1L8F3A3	-	V9GVP7
198	KSKK KSRKGRPRK									
199	RRMI NKIDKNEDRK KDLS	168	Q96RN5	Mediator of RNA polymerase II transcription subunit 15	Component of the Mediator complex, a coactivator involved in the regulated transcription of nearly all RNA polymerase II-dependent genes.	F1QFA9	C3YXZ1	A0A1L8I0E1	-	F1P1D8
200	KEVGVGFAT RK	169	P15090	Fatty acid-binding protein, adipocyte	Lipid transport protein in adipocytes. Binds both long chain fatty acids and retinoic acid. Delivers long-chain fatty acids and retinoic acid to their cognate receptors in the nucleus.	Q503X5	C3Y8B1	Q6PGR8	K7G6I3	Q90X55
201	KR KRKRV	170	Q9UHL9	General transcription factor II-I repeat domain-containing protein 1	May be a transcription regulator involved in cell-cycle progression and skeletal muscle differentiation.	E7F1R4	-	A0A1L8HG89	K7FZF2	A0A1D5NWB4
202	PAKRKC IF	171	O75928	E3 SUMO-protein ligase PIAS2	Functions as an E3-type small ubiquitin-like modifier (SUMO) ligase, stabilizing the interaction between UBE2I and the substrate, and as a SUMO-tethering factor.	A2CE48	C3Z9X9	Q6IRN4	K7FKR8	A0A1D5P1W4
203	KRTW VQEEEDIHKE KKI	172	Q8NG31	Kinetochore scaffold 1	Performs two crucial functions during mitosis: it is essential for spindle-assembly checkpoint signaling and for correct chromosome alignment.	Q56A53	-	-	-	-
204	KKKKRK	173	Q9ULX6	A-kinase anchor protein 8-like	Could play a role in constitutive transport element (CTE)-mediated gene expression by association with	A2CEZ5	-	A0A1L8GMX3	K7F381	Q5ZJ02
205	KRKL									
206	RKRK	174	Q16665	Hypoxia-inducible factor 1-alpha	Functions as a master transcriptional regulator of the adaptive response to hypoxia.	F1QNB9	C3XS48	Q6GP97	K7FUG2	Q9YIB9
207	RRKEKSKR DREKDKKR	175	Q6GQQ9	OTU domain-containing protein 7B	Negative regulator of the non-canonical NF-kappa-B pathway that acts by mediating deubiquitination of TRAF3, an inhibitor of the NF-kappa-B pathway, thereby acting as a negative regulator of B-cell responses.	X1WEZ5	C3YXE8	-	K7G872	H9L017
208	KKRKK	176	P42568	Protein AF-9	Chromatin reader component of the super elongation complex (SEC), a complex required to increase the catalytic rate of RNA polymerase II transcription by suppressing transient pausing by the polymerase at multiple sites along the DNA	Q568M5	C3Y1V7	Q68EY8	K7FL35	F1NLL5

209	KKRKK	177	Q9UIS9	Methyl-CpG-binding domain protein 1	Transcriptional repressor that binds CpG islands in promoters where the DNA is methylated at position 5 of cytosine within CpG dinucleotides.	Q7SZX5	-	A0A1L8F5I6	-	-
210	PSKRPKA	178	P78347	General transcription factor II-I	Interacts with the basal transcription machinery by coordinating the formation of a multiprotein complex at the C-FOS promoter, and linking specific signal responsive activator complexes.	E7F1R4	-	A0A1L8HG89	K7FZF2	A0A1D5NWB4
211	RRYGPK	179	P50461	Cysteine and glycine-rich protein 3	Positive regulator of myogenesis. Acts as cofactor for myogenic bHLH transcription factors such as MYOD1, and probably MYOG and MYF6.	F1QJC0	C3ZK45	Q68FI9	K7FLU1	F1NWZ2
212	KAKTEEK DLKKLKKQEK EEKDFRKKFK	180	O15117	FYN-binding protein 1	Acts as an adapter protein of the FYN and LCP2 signaling cascades in T-cells. Modulates the expression of interleukin-2 (IL-2). Involved in platelet activation. Prevents the degradation of SKAP1 and SKAP2. May play a role in linking T-cell signaling to remodeling of the actin cytoskeleton.	UPI0004F429CD	C3YID4	A0A1L8I2G1	-	E1C908
213	RKRRK	181	Q8WW38	Zinc finger protein ZFPM2	Transcription regulator that plays a central role in heart morphogenesis and development of coronary vessels from epicardium, by regulating genes that are essential during cardiogenesis.	Q2QKJ8	C3YFM2	A0A1L8FZV8	K7F9W8	A0A1D5PTA6
214	RKR GKPASGAGAG AGAGKRRRKA DSAGDRGKSK	182	O43818	U3 small nucleolar RNA-interacting protein 2	Component of a nucleolar small nuclear ribonucleoprotein particle (snoRNP) thought to participate in the processing and modification of pre-ribosomal RNA.	F1R3Q7	C3Z2P5	A0A1L8GHE5	-	-
215	KRSE SWTLKKPKSV SKKLK	183	Q8IYM9	E3 ubiquitin-protein ligase TRIM22	Interferon-induced antiviral protein involved in cell innate immunity. The antiviral activity could in part be mediated by TRIM22-dependent ubiquitination of viral proteins.	A0A0R4ISE2	-	A0A1L8HI93	K7FKU0	A0A1D5PYD2
216	KKKTEGLVKL TPIDKRK	184	O15164	Transcription intermediary factor 1-alpha	Transcriptional coactivator that interacts with numerous nuclear receptors and coactivators and modulates the transcription of target genes.	Q5RHY8	C3ZKJ9	Q56R14	-	F1NBP6
217	RAKKGRR	185	Q15326	Zinc finger MYND domain-containing protein 11	Chromatin reader that specifically recognizes and binds histone H3.3 trimethylated at 'Lys-36' (H3.3K36me3) and regulates RNA polymerase II elongation.	Q08CT0	C3Z8L5	A0A1L8FVB2	K7FYY9	E1BUC1
218	PPS RRHHCRSKAK RSR	186	O15534	Period circadian protein homolog 1	Transcriptional repressor which forms a core component of the circadian clock.	F1QUT1	C3XQ83	Q9DG29	-	A0A1D5P3Z1
219	KRGI TNTLEESSSL KRKR	187	P28715	DNA repair protein complementing XP-G cells	Single-stranded structure-specific DNA endonuclease involved in DNA excision repair.	F1QJS3	C3ZF00	A0A1L8HH45	K7GJZ2	Q4AEJ2
220	KLVPGLFKNE MKRRR	188	P35226	Polycomb complex protein BMI-1	Component of a Polycomb group (PcG) multiprotein PRC1-like complex, a complex class required to maintain the transcriptionally repressive state of many genes, including Hox genes, throughout development.	B0UXV8	C3YW14	Q640D5	K7FHU3	F1NWQ4
221	RADRD KKKSRKRH	189	P19447	General transcription and DNA repair factor IIH helicase subunit XPB	ATP-dependent 3'-5' DNA helicase, component of the general transcription and DNA repair factor IIH (TFIIH) core complex, which is involved in general and transcription-coupled nucleotide excision repair (NER) of damaged DNA and, when complexed to CAK, in RNA transcription by RNA polymerase II.	Q6PFM0	C3ZFA3	A0A1L8EUJ7	K7FSE8	Q5ZMK5
222	KKKKR K	190	Q92889	DNA repair endonuclease XPF	Catalytic component of a structure-specific DNA repair endonuclease responsible for the 5-prime incision during DNA repair. Involved in homologous recombination that assists in removing interstrand cross-link	Q7SYE8	C3ZNX8	A0A1L8EXK1	-	F1NAV2
223	KRPRD DEEEEQKMRR KQT	191	Q8WYA6	Beta-catenin-like protein 1	Component of the PRP19-CDC5L complex that forms an integral part of the spliceosome and is required for activating pre-mRNA splicing. Participates in AID/AICDA-mediated Ig class switching recombination (CSR). May induce apoptosis.	Q7SYE8	C4A0X5	A0A1L8EXK1	K7FZ60	E1BW50
224	KKRKAP	192	Q9P0K7	Ankycorbin	Plays a role in actin regulation at the ectoplasmic specialization, a type of cell junction specific to testis. Important for establishment of sperm polarity and normal spermatid adhesion. May also promote integrity of Sertoli cell tight junctions at the blood-testis barrier.	F1R8J7	C3YNR4	A0A1L8I2I5	K7G1V5	A0A1D5P991

225	KRFARGDKR GKLPRW	193	P18074	General transcription and DNA repair factor IIH helicase subunit XPD	ATP-dependent 5'-3' DNA helicase, component of the general transcription and DNA repair factor IIH (TFIIH) core complex, which is involved in general and transcription-coupled nucleotide excision repair (NER) of damaged DNA and, when complexed to CAK, in RNA transcription by RNA polymerase II.	F1R345	C3ZNC0	A0A1L8HG08	-	F1NN99
226	RRRVRSF ISPIPSKR	194	Q9UGU0	Transcription factor 20	Transcriptional activator that binds to the regulatory region of MMP3 and thereby controls stromelysin expression. It stimulates the activity of various transcriptional activators such as JUN, SP1, PAX6 and [illegible]	E7F726	-	0A1L8GGN6	-	E1BXI6
227	KPKKQ RQRRERRKPG AQPRKRKTKQ									
228	RFKRRH RS									
229	RAKKVRR	195	Q9UEW8	STE20/SPS1-related proline-alanine-rich protein kinase	May act as a mediator of stress-activated signals. Mediates the inhibition of SLC4A4, SLC26A6 as well as CFTR activities by the WNK scaffolds, probably through phosphorylation.	A5PLJ7	C3ZQX3	A0JPG0	-	F1NSM5
230	KKLY QVKGKK	196	P40121	Macrophage-capping protein	Calcium-sensitive protein which reversibly blocks the barbed ends of actin filaments but does not sever preformed actin filaments. May play an important role in macrophage function. May play a role in regulating cytoplasmic and/or nuclear structures through potential interactions with actin. May bind DNA.	E7FC92	C3YDZ9	Q6GMC0	K7FNI5	P02640
231	KRK PLP	197	P42681	Tyrosine-protein kinase TXK	Non-receptor tyrosine kinase that plays a redundant role with ITK in regulation of the adaptive immune response.	O42200	C3Z633	A0A1L8HTV0	-	E1BTT3
232	KKRSK	198	Q01831	DNA repair protein complementing XP-C cells	Involved in global genome nucleotide excision repair (GG-NER) by acting as damage sensing and DNA-binding factor component of the XPC complex.	Q1LVE4	C3YAV9	A0A1L8GPS3	K7GA72	E1BUG1
233	KRPMEEDGEE KSPSKKKKK	199	Q12906	Interleukin enhancer-binding factor 3	RNA-binding protein that plays an essential role in the biogenesis of circular RNAs (circRNAs) which are produced by back-splicing circularization of pre-mRNAs.	Q6NXA4	C4A0T6	Q6DCD0	-	A0A1D5NXI6
234	RKRKK	200	P54132	Bloom syndrome protein	ATP-dependent DNA helicase that unwinds single- and double-stranded DNA in a 3'-5' direction	Q498Z7	C3YKR1	Q32NN1	K7F4Y6	Q8AYS4
235	KKKKK	201	P38935	DNA-binding protein SMUBP-2	5' to 3' helicase that unwinds RNA and DNA duplices in an ATP-dependent reaction.	M9MM98	C3YJY8	A0A1L8GJ95	-	E1BY42
236	KDEVRRGD TSTEDIQEEK DKK	202	Q7Z401	C-myc promoter-binding protein	Probable guanine nucleotide exchange factor (GEF) which may activate RAB10. Promotes the exchange of GDP to GTP, converting inactive GDP-bound Rab proteins into their active GTP-bound form.	A0A286Y972	C3Y2D3	A0A1L8HXV3	K7FZL9	A0A1D5PD21
237	KKKKK	203	P00519	Tyrosine-protein kinase ABL1	Non-receptor tyrosine-protein kinase that plays a role in many key processes linked to cell growth and survival such as cytoskeleton remodeling in response to [illegible]	A0A0R4IYB8	-	Q6P282	K7FHZ2	F1NCD9
238	S KRFLR									
239	PRKRAGE									
240	KKR REKQRRR	204	O43823	A-kinase anchor protein 8	Anchoring protein that mediates the subcellular compartmentation of cAMP-dependent protein kinase (PKA type II)	A0A0H2UKW6	-	A0A1L8GMX3	K7F381	A0A1D5PZG6
241	K RKRKSR	205	Q8IWZ8	SURP and G-patch domain-containing protein 1	Plays a role in pre-mRNA splicing.	E7FGI4	C3ZA13	Q4V7M9	-	A0A1D5P6Z4
242	KKK RLR	206	P31629	Transcription factor HIVEP2	This protein specifically binds to the DNA sequence 5'-GGGACTTTCC-3' which is found in the enhancer elements of numerous viral promoters such as those of SV40, CMV, or HIV1.	F1Q5D6	-	Q5MYB9	K7FQK1	F1P1B9
243	KKEMIAQQR EYKKKKALKK	207	O75940	Survival of motor neuron-related-splicing factor 30	Necessary for spliceosome assembly. Overexpression causes apoptosis.	Q9W6S8	C3Y8H2	Q6NU78	K7G033	Q98SU9
244	KRCIWSTPKR RHKKK	208	Q9HB58	Sp110 nuclear body protein	Transcription factor. May be a nuclear hormone receptor coactivator. Enhances transcription of genes [illegible]	F1R6N2	-	A0A1L8GBC5	-	-
245	KKK EKDICSSSKR RFQK									
246	PGLTKRVK KSK	209	Q96EZ8	Microspherule protein 1	Modulates the transcription repressor activity of DAXX by recruiting it to the nucleolus	Q6P0E3	C3YYL7	Q7T0W8	-	A0A1D5PQU2
247	KKRKR	210	Q15637	Splicing factor 1	Necessary for the ATP-dependent first step of spliceosome assembly. Binds to the intron branch point sequence (BPS) 5'-UACUAAC-3' of the pre-mRNA. May act as transcription repressor.	A0A0R4IBT0	C3YFS6	-	-	-

248	KKRNKKKK	211	Q9UBZ9	DNA repair protein REV1	Deoxycytidyl transferase involved in DNA repair. Transfers a dCMP residue from dCTP to the 3'-end of a DNA primer in a template-dependent reaction. May assist in the first step in the bypass of abasic lesions by the insertion of a nucleotide opposite the lesion. Required for normal induction of mutations by physical and chemical agents.	B0UY97	C3Y6K1	A0A1L8HGZ7	-	F1P5H4
249	RRWNK LR	212	Q03468	DNA excision repair protein ERCC-6	Essential factor involved in transcription-coupled nucleotide excision repair which allows RNA [illegible]	F1RDN1	C3Z749	A0A1L8FKT9	-	E1BYA8
250	KRRI QPAFGADHDV PKRKK									
251	RKRSR	213	O43502	DNA repair protein RAD51 homolog 3	Essential for the homologous recombination (HR) pathway of DNA repair. Involved in the homologous recombination repair (HRR) pathway of double-stranded DNA breaks arising during DNA replication or induced by DNA-damaging agents.	F1QQC9	-	A0A1L8HG81	K7G3U0	A0A1L1RWC9
252	KDKFN	214	Q9UBB9	Tuftelin-interacting protein 11	Involved in pre-mRNA splicing, specifically in spliceosome disassembly during late-stage splicing events.	Q6DI35	-	Q66J74	-	Q5ZII9
253	KEKFKENGKM FKDK	215	Q03112	MDS1 and EVI1 complex locus protein	Functions as a transcriptional regulator binding to DNA sequences in the promoter region of target genes and regulating positively or negatively their expression. Oncogene which plays a role in development, cell proliferation and differentiation. May also play a role in apoptosis through regulation of the JNK and TGF-beta signaling. Involved in hematopoiesis.	F1Q833	C3XYB0	-	K7FB80	A0A1D5P950
254	PSRKRPR	216	Q92833	Protein Jumonji	Regulator of histone methyltransferase complexes that plays an essential role in embryonic development, including heart and liver development, neural tube fusion process and hematopoiesis.	Q1LVC2	C3ZYE0	A0A1L8FY27	-	Q5F363
255	KK TGKNRKLKSK RVKPR	217	O15055	Period circadian protein homolog 2	Transcriptional repressor which forms a core component of the circadian clock.	F1QUT1	C3XQ83	Q9DG29	-	A0A1D5P3Z1
256	RK KRRRQKSRK	218	Q5H9F3	BCL-6 corepressor-like protein 1	Transcriptional corepressor. May specifically inhibit gene expression when recruited to promoter regions by sequence-specific DNA-binding proteins such as BCL6. This repression may be mediated at least in part by histone deacetylase activities which can associate with this corepressor.	F1RCL8	-	A0A1L8F7G7	K7G927	A0A1D5P6S5
257	RKRPL EAPPEAGSTK RTN	219	P51513	RNA-binding protein Nova-1	May regulate RNA splicing or metabolism in a specific subset of developing neurons	Q1LYC7	-	Q6GPZ4	K7FI95	R4GG35
258	KRK MMQPFNKPSG TFIKKPKLAK	220	Q5BKZ1	DBIRD complex subunit ZNF326	Core component of the DBIRD complex, a multiprotein complex that acts at the interface between core mRNP particles and RNA polymerase II (RNAPII) and integrates transcript elongation with the regulation of alternative splicing: the DBIRD complex affects local transcript elongation rates and alternative splicing of a large set of exons embedded in (A + T)-rich DNA regions.	A2CEZ5	-	A0A1L8GMX3	K7F381	Q5ZJ02
259	KRKRE KKEKKKKRK	221	P25440	Bromodomain-containing protein 2	Binds hyperacetylated chromatin and plays a role in the regulation of transcription, probably by chromatin remodeling. Regulates transcription of the CCND1 gene. Plays a role in nucleosome assembly.	A8CYR1	C3ZPB3	A0A1L8H2F1	-	F1NS89
260	KKP KRKKRRRR	222	Q96CK0	Zinc finger protein 653	Transcriptional repressor. May repress NR5A1, PPARG, NR1H3, NR4A2, ESR1 and NR3C1 [illegible]	UPI00038372B4	C3ZAX3	Q08B30	-	-
261	KEPEKRR RSKRSR									
262	KRKIR	223	P0C1S8	Wee1-like protein kinase 2	Oocyte-specific protein tyrosine kinase that phosphorylates and inhibits CDK1 and acts as a key regulator of meiosis during both prophase I and metaphase II.	Q6DRI0	C3Y7K0	Q8AYK6	K7FYG3	Q5F400
263	RKRRR	224	Q8WXF8	DNA-binding death effector domain-containing protein 2	May play a critical role in death receptor-induced apoptosis and may target CASP8 and CASP10 to the [illegible]	Q6DHN2	C3XZG0	Q5PQ51	K7GE20	A0A1D5PXF2
264	KRQRRS RGRPSGGARR RRR									
265	KDKEKEK QRKEKDKTR	225	Q8TE49	OTU domain-containing protein 7A	Has deubiquitinating activity towards 'Lys-11'-linked polyubiquitin chains.	X1WEZ5	C3YXE8	A0A1L8H082	K7G872	E1C900
266	RKK	226	Q9Y473	Zinc finger protein 175	Down-regulates the expression of several chemokine receptors. Interferes with HIV-1 replication by suppressing Tat-induced viral LTR promoter activity.	A9JTD1	C3XPD3	A0A1L8FUG5	K7FQR3	Q5F3E3
267	KKKTGARK K	227	Q9Y3S2	Zinc finger protein 330	zinc ion binding	Q7ZUZ8	C3YZ79	Q7ZXJ2	K7GHZ0	E1C2N4

268	KKHK	228	Q6ECI4	Zinc finger protein 470	May be involved in transcriptional regulation.	Q4V8R6	C3XSV2	Q6GN31	K7GAQ9	-
269	RHRR									
270	KRRKF	229	Q86X24	HORMA domain-containing protein 1	Plays a key role in meiotic progression. Regulates 3 different functions during meiosis: ensures that sufficient numbers of processed DNA double-strand breaks (DSBs) are available for successful homology search by increasing the steady-state numbers of single-stranded DSB ends.	A2BF66	C3Y7J4	A0A1L8F4F2	-	E1C2C4
271	K NKKKKEK	230	Q96T21	Selenocysteine insertion sequence-binding protein 2	Binds to the SECIS element in the 3'-UTR of some mRNAs encoding selenoproteins. Binding is stimulated by SELB.	Q7ZV23	C3ZJL2	A0A1L8HYH8	K7GDC3	F1NS30
272	RREREAEHGY ASMLPYNNKD RDALKRRNK	231	Q05195	Max dimerization protein 1	Transcriptional repressor. MAD binds with MAX to form a sequence-specific DNA-binding protein complex which recognizes the core sequence 5'-CAC[GA]TG-3'. MAD thus antagonizes MYC transcriptional activity by competing for MAX.	A3KQG0	C3Y472	B7ZPQ0	K7G942	Q5ZJ78
273	KKTDKLKKKK KRKRR	232	Q86YI8	PHD finger protein 13	Modulates chromatin structure. Required for normal chromosome condensation during the early stages of mitosis. Required for normal chromosome separation during mitosis.	A0A0G2L648	C3ZH10	Q5PQ67	K7FYN5	R4GGG1
274	R KHVKAHSSKE QQARKKLR	233	Q8NEA6	Zinc finger protein GLIS3	Acts as both a repressor and activator of transcription. Binds to the consensus sequence 5'-GACCACCCAC-3'	A0A0R4IU95	C3XY75	A0A1L8HY67	-	E1C1Q5
275	KRPA SDMGKKPKTP KKKKKK	234	O94900	Thymocyte selection-associated high mobility group box protein TOX	May play a role in regulating T-cell development.	B0V356	-	A0A1L8FZ33	K7FWE5	A0A1D5PSC7
276	KKKTTKL	235	Q96NK8	Neurogenic differentiation factor 6	Activates E box-dependent transcription in collaboration with TCF3/E47. May be a trans-acting factor involved in the development and maintenance of the mammalian nervous system. Transactivates the promoter of its own gene	O42202	C3ZXA2	A0A1L8HIF8	K7EYZ4	P79766
277	KKRRH x{9}HK KERKVY	236	Q13627	Dual specificity tyrosine-phosphorylation-regulated kinase 1A	Dual-specificity kinase which possesses both serine/threonine and tyrosine kinase activities. May play a role in a signaling pathway regulating nuclear functions of cell proliferation. Modulates alternative splicing by phosphorylating the splice factor SRSF6	A1L1U2	C3Z2G7	A0A1L8HCE8	K7FXM2	Q8JG32
278	KKVRK	237	P15923	Transcription factor E2-alpha	Transcriptional regulator. Involved in the initiation of neuronal differentiation.	B1H1L4	C3ZAG1	A0A1L8HWH1	K7F8X5	A0A1D5P7W4
279	VKKSIR	238	P06748	Nucleophosmin	Involved in diverse cellular processes such as ribosome biogenesis, centrosome duplication, protein chaperoning, histone assembly, cell proliferation, and regulation of tumor suppressors p53/TP53 and ARF.	E7FCU0	-	Q7ZTP6	-	P16039
280	KRKH RKIPFSKRK	239	Q9UKK3	Poly [ADP-ribose] polymerase 4	NAD+ ADP-ribosyltransferase activity	UPI0007EE98CA	C3Y4W7	A0A1L8HJ59	-	A0A1D5PL17
281	KKNK	240	Q86Z02	Homeodomain-interacting protein kinase 1	Serine/threonine-protein kinase involved in transcription regulation and TNF-mediated cellular apoptosis.	E7F544	C3Y5H6	Q5U4V9	K7F7D3	E1BW82
282	KKERNS SGMARKAKRT K	241	P35659	Protein DEK	Involved in chromatin organization.	F1R6C4	-	Q2KHP5	K7G4P3	Q5F422
283	KRRKK	242	O75398	Deformed epidermal autoregulatory factor 1 homolog	Transcription factor that binds to sequence with multiple copies of 5'-TTC[CG]G-3' present in its own promoter and that of the HNRPA2B1 gene.	B3DHN2	C3ZC54	A0A1L8GJK0	-	A0A1L1RRQ7
284	KKK	243	P35251	Replication factor C subunit 1	The elongation of primed DNA templates by DNA polymerase delta and epsilon requires the action of the accessory proteins PCNA and activator 1.	F1Q5P6	C3Y4Y3	A0A1L8HTJ6	-	F1NEP9
285	KIPKFKK	244	Q09666	Neuroblast differentiation-associated protein AHNAK	May be required for neuronal cell differentiation.	F1QZ50	C3YDK3	A0A1L8F3W5	-	-
286	KK SKFKMPK									
287	KIKAKK									
288	KLKKS KIKMPK									
289	KRPRK	245	Q15554	Telomeric repeat-binding factor 2	Binds the telomeric double-stranded 5'-TTAGGG-3' repeat and plays a central role in telomere maintenance and protection against end-to-end fusion of chromosomes.	Q6NW47	C3ZQ21	Q1WM12	-	F1NSI5

290	KK RRR	246	Q86Y91	Kinesin-like protein KIF18B	In complex with KIF2C, constitutes the major microtubule plus-end depolymerizing activity in mitotic cells. Its major role may be to transport KIF2C and/or MAPRE1 along microtubules	Q7ZUW9	C3Y5L4	A0A1L8GJ38	K7F9Y7	Q5ZLK6
291	KKKKPK	247	Q8IX01	SURP and G-patch domain-containing protein 2	May play a role in mRNA splicing.	E7FGI4	C3ZA13	Q4V7M9	-	E1C5N4
292	KAKKRKR	248	O14776	Transcription elongation regulator 1	Transcription factor that binds RNA polymerase II and inhibits the elongation of transcripts from target promoters. Regulates transcription elongation in a TATA box-dependent manner. Necessary for TAT-dependent activation of the human immunodeficiency virus type 1 (HIV-1) promoter.	E7F3A6	C3Y4A6	-	K7GCU0	A0A1D5NTY1
293	R RREAAAEARK SHSPVKRPRK	249	P78549	Endonuclease III-like protein 1	Bifunctional DNA N-glycosylase with associated apurinic/apyrimidinic (AP) lyase function that catalyzes the first step in base excision repair (BER), the primary repair pathway for the repair of oxidative DNA damage.	F1QBP9	C3Z7H4	-	-	A7M7B9
294	KK PKIRRKKS	250	Q7Z7C8	Transcription initiation factor TFIID subunit 8	Transcription factor TFIID is one of the general factors required for accurate and regulated initiation by RNA polymerase II.	Q6P0T2	C3ZC47	Q7ZYA2	K7FSI0	R4GIW1
295	HRKRK R	251	Q7Z2E3	Aprataxin	DNA-binding protein involved in single-strand DNA break repair, double-strand DNA break repair and base excision repair	P61799	C3ZFB4	Q7T287	-	A0A1D5PAK1
296	RPRRRSRR	252	Q7Z628	Neuroepithelial cell-transforming gene 1 protein	Acts as guanine nucleotide exchange factor (GEF) for RhoA GTPase. May be involved in activation of the [illegible]	Q1LXH8	C3Z2Q7	A0A1L8GZH7	K7FZV1	Q5ZJM0
297	KRKRR EK									
298	KR RRPSEGR	253	Q7Z5J4	Retinoic acid-induced protein 1	Transcriptional regulator of the circadian clock components: CLOCK, ARNTL/BMAL1, [illegible]	E7F726	-	A0A1L8EYP3	-	-
299	KRKSAFMA PVPTKKRNLV									
300	KKSSQTK KKKKAFNSPK	254	Q9NPC7	Myoneurin	RNA polymerase II transcription factor activity, sequence-specific DNA binding	A9JSZ9	C3XQ44	A0A1L8HK33	K7F9H0	E1C3P7
301	KRKR GK									
302	KRLEH TERQFRNRRK	255	Q8IY67	Ribonucleoprotein PTB-binding 1	Cooperates with PTBP1 to modulate regulated alternative splicing events. Promotes exon skipping. Cooperates with PTBP1 to modulate switching between mutually exclusive exons during maturation of the TPM1 pre-mRNA	F1QAP4	C3YIG5	A0A1L8GLV5	-	A0A1D5P0I3
303	KMISRKK KHQA	256	Q14517	Protocadherin Fat 1	Plays an essential role for cellular polarization, directed cell migration and modulating cell-cell contact.	A2BGT8	C3Y3A1	A0A1L8GWK1	K7FFG3	F1NWW5
304	KRPAEPG PARVGKKGKK	257	Q8N543	Prolyl 3-hydroxylase OGFOD1	Prolyl 3-hydroxylase that catalyzes 3-hydroxylation of 'Pro-62' of small ribosomal subunit uS12 (RPS23), thereby regulating protein translation termination efficiency. Involved in stress granule formation	F1QTC6	-	Q6DE73	-	-
305	RK KF	258	P07992	DNA excision repair protein ERCC-1	Non-catalytic component of a structure-specific DNA repair endonuclease responsible for the 5'-incision during DNA repair.	Q6NY87	C3Y937	A0A1L8FKK3	-	-
306	KKKK MTK	259	Q13562	Neurogenic differentiation factor 1	Acts as a transcriptional activator: mediates transcriptional activation by binding to E box-containing promoter consensus core sequences 5'-CANNTG-3'.	O42202	-	A0A1L8ERW7	K7F0H1	P79765
307	KKRKKK	260	O94842	TOX high mobility group box family member 4	Component of the PTW/PP1 phosphatase complex, which plays a role in the control of chromatin structure and cell cycle progression during the transition from mitosis into interphase.	F1QXS5	-	A0A1L8HPM5	K7FLV4	A0A1D5PEI4
308	RKKRGRK	261	Q14781	Chromobox protein homolog 2	Component of a Polycomb group (PcG) multiprotein PRC1-like complex, a complex class required to maintain the transcriptionally repressive state of many genes, including Hox genes, throughout development	Q8JIQ9	-	A0A1L8EM82	-	-
309	KRKRx{21}KFRKSKKKKR	262	P57052	Splicing regulator RBM11	Tissue-specific splicing factor with potential implication in the regulation of alternative splicing during neuron and germ cell differentiation.	Q6NWB3	C3Z7L7	Q6GM50	K7FCD7	A0A1L1RL91
310	RKRLSD EKNLPLDGKR QRF	263	Q6PIY7	Poly(A) RNA polymerase GLD2	Cytoplasmic poly(A) RNA polymerase that adds successive AMP monomers to the 3'-end of specific RNAs, forming a poly(A) tail. In contrast to the canonical nuclear poly(A) RNA polymerase, it only adds poly(A) to selected cytoplasmic mRNAs	Q503I9	C3Y1Z0	A0A1L8I240	K7FPN3	E1C833

311	RKPR	264	Q9NR83	SLC2A4 regulator	Transcription factor involved in SLC2A4 and HD gene transactivation. Binds to the consensus sequence 5'-GCCGGCG-3'	F1R143	C3YKU5	A0A1L8GC36	K7FHK4	A0A1D5PYE8
312	KPKRKRR KKRGHGWSRM RMRR	265	Q13342	Nuclear body protein SP140	Component of the nuclear body, also known as nuclear domain 10, PML oncogenic domain, and KR body (PubMed:8910577). May be involved in the pathogenesis of acute promyelocytic leukemia and viral infection	A0A0R4IMH4	-	A0A1L8GBC5	-	-
313	KRKGPDS LSDGPACKR	266	Q9BYH8	NF-kappa-B inhibitor zeta	Involved in regulation of NF-kappa-B transcription factor complexes.	UPI0007EEA726	C3ZHK3	A0A1L8H8D6	K7GAL9	F1NL80
314	H KRKR	267	Q8NEA9	Germ cell-less protein-like 2	Possible function in spermatogenesis. Probable substrate-specific adapter of an E3 ubiquitin-protein [illegible]	Q6NYQ2	C3Y031	A0A1L8H1G4	K7FJA3	-
315	RKKL R									
316	KKRGPKKRK MTK	268	Q15784	Neurogenic differentiation factor 2	Transcriptional regulator implicated in neuronal determination. Mediates calcium-dependent transcription activation by binding to E box-containing promoter.	Q9W6C8	C3ZXA2	Q6GPW1	K7EXA8	P79765
317	H KRKR	269	Q96IK5	Germ cell-less protein-like 1	Possible function in spermatogenesis. Enhances the degradation of MDM2 and increases the amount of p53 [illegible]	Q6NYQ2	C3Y031	A0A1L8H1G4	K7FJA3	-
318	PRRKKL K									
319	KRKNE KAGSKRKK	270	Q9Y3E1	Hepatoma-derived growth factor-related protein 3	Enhances DNA synthesis and may play a role in cell proliferation.	A0A0R4I9P3	-	A0A1L8HXV6	-	R4GLJ4
320	FKRR	271	Q86VE0	Myb-related transcription factor, partner of profilin	Transcriptional repressor; DNA-binding protein that specifically recognizes the core sequence 5'-YAAC[GT]G-3'. Dimerization with PFN1 reduces its [illegible]	UPI00038387C8	-	A0A1L8F865	-	-
321	HKRRK									
322	R KRR									
323	KK R	272	Q92630	Dual specificity tyrosine-phosphorylation-regulated kinase 2	Serine/threonine-protein kinase involved in the regulation of the mitotic cell cycle, cell proliferation, apoptosis, organization of the cytoskeleton and neurite outgrowth.	A0A0R4ITS5	C3Y4L0	Q5PPW5	K7F6T0	Q5ZIU3
324	KKEHKKVK	273	P42166	Lamina-associated polypeptide 2, isoform alpha	May be involved in the structural organization of the nucleus and in the post-mitotic nuclear assembly. Plays an important role, together with LMNA, in the nuclear anchorage of RB1.<br>TP and TP5 may play a role in T-cell development and function. TP5 is an immunomodulating pentapeptide	Q5RGX2	-	-	-	-
325	QKRKT	274	P41218	Myeloid cell nuclear differentiation antigen	May act as a transcriptional activator/repressor in the myeloid lineage. Plays a role in the granulocyte/monocyte cell-specific response to interferon. Stimulates the DNA binding of the transcriptional repressor protein YY1	UPI00038371A7	-	-	-	-
326	KKTQRRH	275	Q9P0U3	Sentrin-specific protease 1	Protease that catalyzes two essential functions in the SUMO pathway	A4IFY7	C3Z396	-	K7GGL1	A0A1D5PIW4
327	YF RKR									
328	KKRK									
329	RKKREK	276	P22626	Heterogeneous nuclear ribonucleoproteins A2/B1	Heterogeneous nuclear ribonucleoprotein (hnRNP) that associates with nascent pre-mRNAs, packaging them into hnRNP particles.	Q803K3	-	Q6IP29	-	Q5ZME1
330	KKRKER	277	O43390	Heterogeneous nuclear ribonucleoprotein R	Component of ribonucleosomes, which are complexes of at least 20 other different heterogenious nuclear ribonucleoproteins (hnRNP). hnRNP play an important role in processing of precursor mRNA in the nucleus.	Q7SZC9	C3ZLB6	A0A1L8G9H1	-	A0A1D5P155
331	RRRR	278	Q9H4L4	Sentrin-specific protease 3	Protease that releases SUMO2 and SUMO3 monomers from sumoylated substrates, but has only weak activity [illegible]	A2BEN4	C3XYA3	A0A1L8G3T3	K7GGL1	F1NYZ3
332	WGRHRGRRR									
333	HKVLPR	279	P06401	Progesterone receptor	The steroid hormones and their receptors are involved in the regulation of eukaryotic gene expression and affect cellular proliferation and differentiation in target tissues. Depending on the isoform, progesterone receptor functions as transcriptional activator or repressor.	A4GT83	-	Q9DDU9	-	P07812
334	KKKKRK R	280	Q9NQB0	Transcription factor 7-like 2	Participates in the Wnt signaling pathway and modulates MYC expression by binding to its promoter in a sequence-specific manner.	Q9YHE8	C3Z5P5	A0A1L8HM07	K7GJ95	A0A1D5P979
335	KRKAASQER EAKETERKRR K	281	O94850	Dendrin	Promotes apoptosis of kidney glomerular podocytes. Podocytes are highly specialized cells essential to the ultrafiltration of blood, resulting in the extraction of urine and the retention of protein	E7FAR1	-	-	-	-

336	K RTSSPHKEES PKKTK	282	P51003	Poly(A) polymerase alpha	Polymerase that creates the 3'-poly(A) tail of mRNA's. Also required for the endoribonucleolytic cleavage reaction at some polyadenylation sites.	Q5SNY2	C3Z3M0	A0A1L8F081	-	A0A1D5P5T8
337	KKKRR	283	Q8IZX4	Transcription initiation factor TFIID subunit 1-like	May act as a functional substitute for TAF1/TAFII250 during male meiosis, when sex chromosomes are transcriptionally silenced.	Q1LYC2	C3Y3A3	A0A1L8F6Z1	-	B5AHE5
338	KRKR	284	Q9NZC7	WW domain-containing oxidoreductase	Putative oxidoreductase. Acts as a tumor suppressor and plays a role in apoptosis. Required for normal bone development	Q803A8	C3ZVC1	Q63ZM3	-	Q5F389
339	KKKR K	285	Q9Y3Y4	Pygopus homolog 1	Involved in signal transduction through the Wnt pathway.	A8WG61	C3Z441	A0A1L8H0D5	-	E1BWE1
340	KP RKR	286	Q9H4Z3	Phosphorylated CTD-interacting factor 1	May play a role in transcription elongation or in coupling transcription to pre-mRNA processing through its association with the phosphorylated C-terminal domain (CTD) of RNAPII largest subunit	A0A0R4IKJ1	C3YV22	A0A1L8ETX1	-	E1BR74
341	RRVKEEVLKQ LPPKK	287	Q9H4L7	SWI/SNF-related matrix-associated actin-dependent regulator of chromatin subfamily A containing DEAD/H box 1	DNA helicase that possesses intrinsic ATP-dependent nucleosome-remodeling activity and is both required for DNA repair and heterochromatin organization.	E7F1C4	C3ZNR8	A0A1L8HMC3	K7FTH0	A0A1D5PHR6
342	KKKKKKDR KKKKF	288	Q86X95	Corepressor interacting with RBPJ 1	May modulate splice site selection during alternative splicing of pre-mRNAs	Q504G8	C3Z8J5	A0A1L8EW84	K7GHQ5	E1BVI7
343	RKKRKKNK									
344	NRLRR KR	289	Q9BTC0	Death-inducer obliterator 1	Putative transcription factor, weakly pro-apoptotic when overexpressed	A1A5T6	C3YI02	A0A1L8GA10	K7G4A0	A0A1D5NXL6
345	RLRK KRR									
346	KS PEKKRRK	290	Q9BRQ0	Pygopus homolog 2	Involved in signal transduction through the Wnt pathway.	A8WG61	C3Z441	Q8JHB8	-	E1BWE1
347	KRRPGPG PGVPPKRAR	291	P28340	DNA polymerase delta catalytic subunit	As the catalytic component of the trimeric (Pol-delta3 complex) and tetrameric DNA polymerase delta complexes (Pol-delta4 complex), plays a crucial role in high fidelity genome replication, including in lagging strand synthesis, and repair.	Q2KNE0	C3Y5K6	Q642R8	-	F1NQT0
348	KKKGR	292	Q9Y6F1	Poly [ADP-ribose] polymerase 3	Involved in the base excision repair (BER) pathway, by catalyzing the poly(ADP-ribosyl)ation of a limited number of acceptor proteins involved in chromatin architecture and in DNA metabolism.	Q7ZVB0	C3Z2P3	Q5M9A2	-	E1BSI0
349	KKKRRSR	293	P36402	Transcription factor 7	Transcriptional activator involved in T-cell lymphocyte differentiation. Necessary for the survival of CD4+ CD8+ immature thymocytes. Isoforms lacking the N-terminal CTNNB1 binding domain cannot fulfill this role.	A5A8K1	-	-	-	-
350	KKKRKHR	294	Q92800	Histone-lysine N-methyltransferase EZH1	Polycomb group (PcG) protein. Catalytic subunit of the PRC2/EED-EZH1 complex, which methylates 'Lys-27' of histone H3, leading to transcriptional repression of the affected target gene.	Q08BS4	C3YCV4	Q4V863	-	A0A1D5PN50
351	KREGDDW SQLNVLKKRR	295	Q9BWG6	Sodium channel modifier 1	Plays a role in RNA splicing, possibly contributing to the recognition of non-consensus donor sites.	Q2YDS5	-	A0A1L8FCL5	K7GHG9	-
352	KEDPPNV GKVKKAAKR	296	Q8IZA3	Histone H1oo	May play a key role in the control of gene expression during oogenesis and early embryogenesis, presumably through the perturbation of chromatin structure.	Q4V956	-	Q5XHJ2	-	-
353	KKKKRKREK	297	Q9HCS4	Transcription factor 7-like 1	Participates in the Wnt signaling pathway. Binds to DNA and acts as a repressor in the absence of CTNNB1, and as an activator in its presence.	Q9YHE8	C3Z5P5	A0A1L8GRU8	K7GJ95	A0A1D5P979
354	KRRKKRRH SKVNGLQRGR KK	298	P23497	Nuclear autoantigen Sp-100	Together with PML, this tumor suppressor is a major constituent of the PML bodies, a subnuclear organelle involved in a large number of physiological processes including cell growth, differentiation and apoptosis.	F1R6N2	-	A0A1L8GBC5	-	-
355	KKRW QQRGRKANTR PLKRRRKRGPR									
356	KKCS ETWKTIFAKE KGKF									
357	RRKRR	299	Q8NDT2	Putative RNA-binding protein 15B	RNA-binding protein that acts as a key regulator of N6-methyladenosine (m6A) methylation of RNAs, thereby regulating different processes, such as alternative splicing of mRNAs and X chromosome inactivation mediated by Xist RNA	F1RCY7	-	A0A1L8GHD9	-	F1NRZ4

358	K QKKK	300	Q8IXJ9	Putative Polycomb group protein ASXL1	Probable Polycomb group (PcG) protein involved in transcriptional regulation mediated by ligand-bound nuclear hormone receptors, such as retinoic acid receptors (RARs) and peroxisome proliferator-activated receptor gamma (PPARG).	F1Q5H5	C3YBN3	A0A1L8ERK1	-	A0A1D5PLQ0
