# Supplementary material for: Origin of the nuclear proteome on the basis of pre-existing nuclear localization signals in prokaryotic proteins
Source: Biol Direct. 2020 Apr 28;15:9. doi: 10.1186/s13062-020-00263-6 (PMC7189692; doi:10.1186/s13062-020-00263-6)
Supplement: Supplementary file 2 — Additional file 2: Supplementary Table S2. Prokaryotic proteins with or without predicted NLSs. [file 13062_2020_263_MOESM2_ESM.pdf]

Supplementary Table S2. Prokaryotic proteins with or without predicted NLSs.

Protein	UniProt ID	Organism	Molecular weight, kDa	Molecular weight of EGFP fusion, kDa	Predicted NLSs	In-domain localization of predicted NLS	F_{nuc}/F_{cyt}, mean±s.d.
EGFP	-	-	29.4	-	no	no	1.16±0.10
Prokaryotic proteins with predicted NLSs							
KiaC (S.sp)	Q79PF4	*Synechococcus sp.*	58.0	87.4	KYRARR	KaiC 1 domain (DNA binding)	0.10±0.05
					RRELFRLVAR		
					RRRRT		
					FKMRGSWH	KaiC 2 domain (DNA binding)	
Alr4331	Q8YP69	*Anabaena sp.*	76.2	105.5	RLKRT	S1 domain (interaction with pre-RNA)	0.29±0.07
					RRARTRRSR	no	
					RRRRRR	no	
KiaC (A.sp)	Q8YT40	*Anabaena sp.*	57.9	87.3	RLQYAIRKYKAKR	KaiC 1 domain (DNA binding)	0.33±0.08
LigA (E.coli)	C3T152	*Escherichia coli*	74.0	103.4	RTTLRHHEYLYH	DNA ligase	0.37±0.09
					RITAKRP	Nucleotide-binding pocket	
GyrB	D0XEB0	*Vibrio harveyi*	89.0	118.4	RRGLSLQRYKGLGEMNPDQLWETTMDPETRR	DNA gyrase B subunit	0.48±0.09
LigA (S.sp)	Q5N2P4	*Synechococcus sp.*	74.3	103.7	RSWDQRWRK	nucleotide binding pocket	0.56±0.18
PriA	Q31QI6	*Synechococcus*	89.2	118.6	RRSQRRIRAR	no	1.19±0.27

		sp			RIARRHRW	Primosomal DNA replication, repair, and recombination domains	
RecQ	P15043	*Escherichia coli*	70.0	99.4	RIVALKPK	no	1.26±0.2
					RKLFAKLRKLRKS	The HRDC (interactions with DNA and protein)	
Lig	C6A2U9	*Thermococcus sibiricus*	63.4	92.8	RRFRRKY	DNA-binding site	1.35±0.25
					RLKGGR	no	
PolB	P21189	*Escherichia coli*	90.0	119.4	RLVYRKRLRRPLSEY	DNA polymerase type-II subfamily catalytic domain	1.62±0.30
SigA1	P38023	*Synechococcus sp.*	45.7	75.1	KAKAKVRKTY	no	4.89±1.84
					RRRLFRGRR	Sigma-70 factor domain-2, RNA synthesis	
					KKYMNR	no	
Dcm	P0AED9	*Escherichia coli*	53.0	82.4	WKYLYRYAKKH	SAM-dependent MTase C5-type (DNA interaction, methylation)	7.30±2.71
Control prokaryotic proteins (wighout predicted NLSs)							
NblS	Q8RQ68	*Synechococcus sp.*	73.9	103.3	no	no	0.20±0.06
Tuf	Q8YP63	*Anabaena sp.*	44.8	74.2	no	no	0.26±0.05
ClpB	P63284	*Escherichia coli*	95.6	125.0	no	no	0.28±0.10
GlpK	P0A6F3	*Escherichia coli*	56.2	85.6	no	no	0.31±0.08
Pgi	Q8YY05	*Anabaena sp.*	57.8	87.2	no	no	0.31±0.07

YgiK	P42592	*Escherichia coli*	88.3	117.7	no	no	0.42±0.09
NifJ	Q06879	*Anabaena sp.*	132.2	161.6	no	no	0.44±0.12
AccC	P24182	*Escherichia coli*	49.3	78.7	no	no	0.45±0.13
TynA	P46883	*Escherichia coli*	84.4	113.8	no	no	0.48±0.08
FusA	P0A6M8	*Escherichia coli*	77.6	107.0	no	no	0.50±0.13
Ppc	Q31KY7	*Synechococcus sp.*	117.3	146.7	no	no	0.51±0.12
AchB	Q31PT6	*Synechococcus sp.*	92.3	121.7	no	no	0.63±0.15
DmsA	P18775	*Escherichia coli*	90.4	119.8	no	no	0.79±0.12
PrfC	Q8YP23	*Anabaena sp.*	61.3	90.7	no	no	1.06±0.11
CasA	Q46901	*Escherichia coli*	55.5	84.9	no	no	1.71±0.25
