# Supplementary material for: Origin of the nuclear proteome on the basis of pre-existing nuclear localization signals in prokaryotic proteins
Source: Biol Direct. 2020 Apr 28;15:9. doi: 10.1186/s13062-020-00263-6 (PMC7189692; doi:10.1186/s13062-020-00263-6)
Supplement: Supplementary file 3 — Additional file 3: Supplementary Table S3. Predicted NLSs from prokaryotic proteins are able to target EGFP to the cell nucleus. [file 13062_2020_263_MOESM3_ESM.pdf]

Supplementary Table S3. Predicted NLSs from prokaryotic proteins are able to target EGFP to the cell nucleus.

Protein	Predicted NLSs	In-domain localization of predicted NLS	F_{nuc}/F_{cyt}, mean±s.d.
EGFP	-	-	1.16±0.10
LigA (S. sp.)	RSWDQRWRK	Nucleotide binding pocket	1.29±0.11
PriA	RRSQRRIRAR	no	3.24±0.69
	RIARRHRW	Primosomal DNA replication, repair, and recombination domains	1.98±0.52
RecQ	RIVALKPK	no	1.31±0.14
	RKLFAKLRKLRKS	The HRDC (interactions with DNA and proteins)	4.01±0.49
Lig	RRFRRKY	DNA-binding site	2.32±0.29
	RLKGGR	no	1.34±0.49
PolB	RLVYRKRLRRPLSEY	DNA polymerase type-II subfamily catalytic domain	4.03±0.46
SigA1	KAKAKVRKTY	no	1.74±0.30
	RRRLFRGRR	Sigma-70 factor domain-2, RNA synthesis	2.87±0.41
	KKYMNR	no	1.80±0.58
Dcm	WKYLYRYAKKH	SAM-dependent MTase C5-type (DNA interaction, methylation)	1.80±0.27
