# Supplementary material for: Origin of the nuclear proteome on the basis of pre-existing nuclear localization signals in prokaryotic proteins
Source: Biol Direct. 2020 Apr 28;15:9. doi: 10.1186/s13062-020-00263-6 (PMC7189692; doi:10.1186/s13062-020-00263-6)
Supplement: Supplementary file 4 — Additional file 4: Supplementary Table S4. Detection of NLSs inside prokaryotic proteins by site-directed mutagenesis. [file 13062_2020_263_MOESM4_ESM.pdf]

Supplementary Table S4. Detection of NLSs inside prokaryotic proteins by site-directed mutagenesis.

Protein	Protein F_{nuc}/F_{cyt}, mean±s.d.	Mutant	Mutant F_{nuc}/F_{cyt}, mean±s.d.
PriA	1.19±0.27	mNLS1	1.05±0.15
		mNLS2	1.23±0.31
		mNLS1+mNLS2	1.14±0.18
SigA1	4.89±1.84	mNLS1	1.52±0.27
		mNLS2	0.96±0.16
		mNLS3	1.28±0.16
		mNLS1+ mNLS2	0.66±0.16
		mNLS1+ mNLS3	1.10±0.12
		mNLS2+ mNLS3	0.49±0.15
		mNLS1+ NLS2+mNLS3	0.90±0.21
LigA (S.sp)	0.56±0.18	mNLS	0.79±0.17
PolB	1.62±0.30	mNLS	1.07±0.14
Dcm	7.30±2.71	mNLS	0.76±0.12
RecQ	1.26±0.2	mNLS1	1.32±0.12
		mNLS2	0.96±0.17
		mNLS1+mNLS2	1.01±0.08
Lig	1.35±0.25	mNLS1	1.09±0.14
		mNLS2	1.10±0.12
		mNLS1+mNLS2	1.06±0.12
