# Supplementary material for: Origin of the nuclear proteome on the basis of pre-existing nuclear localization signals in prokaryotic proteins
Source: Biol Direct. 2020 Apr 28;15:9. doi: 10.1186/s13062-020-00263-6 (PMC7189692; doi:10.1186/s13062-020-00263-6)
Supplement: Supplementary file 5 — Additional file 5: Supplementary Fig. S1. Comparison of nuclear accumulation (Fnuc/ Fcyt) of all predicted NLSs and nuclear accumulation (Fnuc /Fcyt) of the full-length proteins fused with EGFP in living HeLa cells. Supplementary Fig. S2. Decrease in nuclear accumulation of prokaryotic proteins by a peptide inhibitor of karyopherin-α (Bimax2). NLS from the T antigen of SV40 virus (NLSSV40) fused with EGFP was used as a positive control. Expression of TagRFP-Bimax2 leads to a decrease in the nuclear accumulation of NLSSV40. A decrease in nuclear accumulation was also detected for five prokaryotic proteins, namely, PriA, Lig, PolB, SigA1 and Dcm. Supplementary Fig. S3. Decrease in the nuclear accumulation of prokaryotic proteins by a peptide inhibitor of karyopherin-β2 (M9M). The NLS from FUS protein (NLSFUS) fused with EGFP was used as a positive control. The expression of TagRFP-M9M leads to a decrease in the nuclear accumulation of NLSFUS. A decrease in nuclear accumulation was also detected for five prokaryotic proteins, namely, PriA, RecQ, Lig, PolB and SigA1. [file 13062_2020_263_MOESM5_ESM.pdf]

Supplementary figures

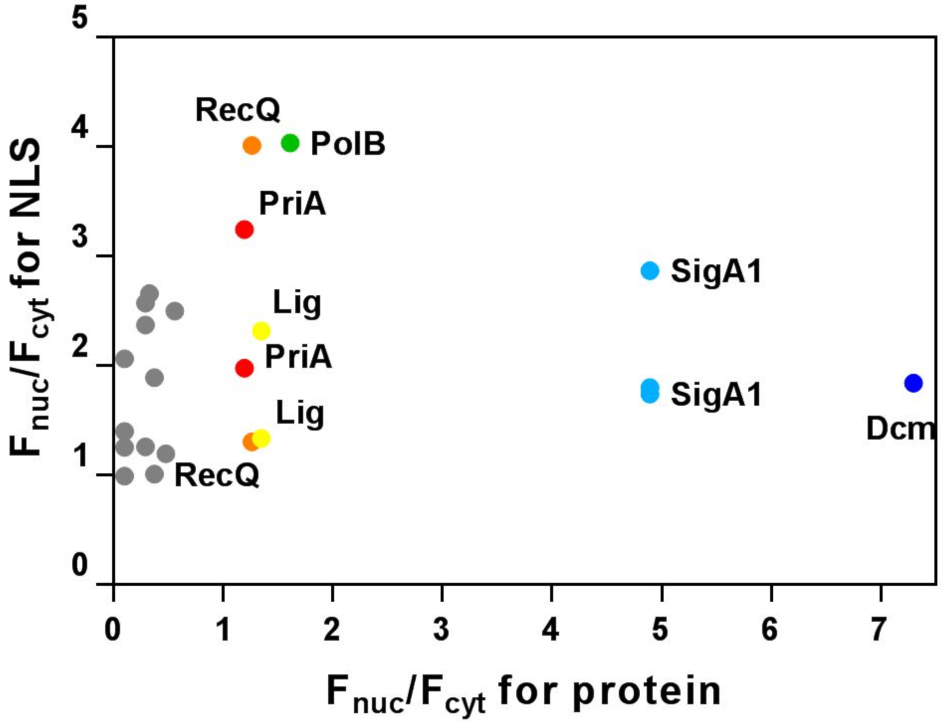

Supplementary Fig. S1. Comparison of nuclear accumulation (F_{nuc}/F_{cyt}) of all predicted NLSs and nuclear accumulation (F_{nuc}/F_{cyt}) of the full-length proteins fused with EGFP in living HeLa cells.

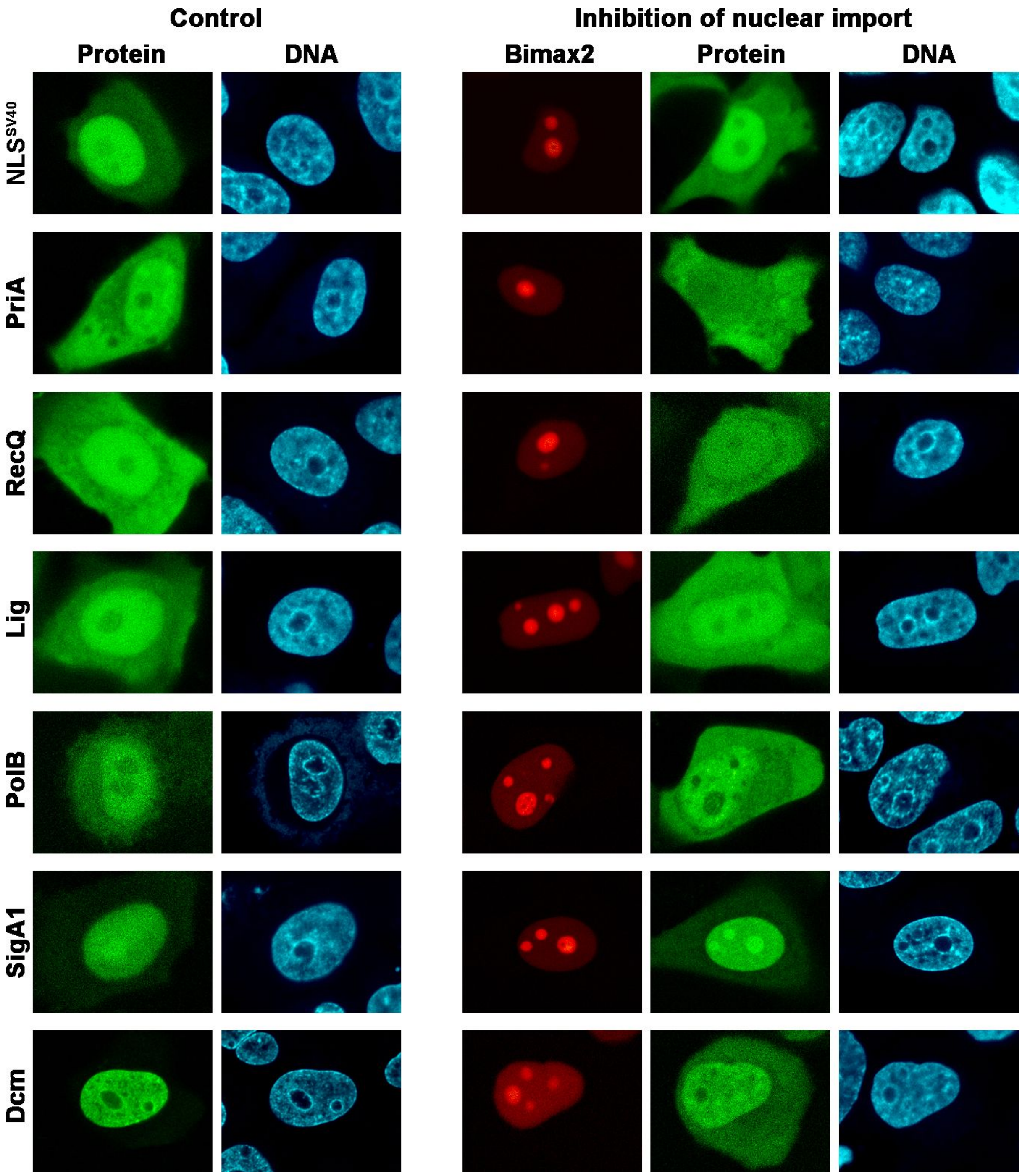

Supplementary Fig. S2. Decrease in nuclear accumulation of prokaryotic proteins by a peptide inhibitor of karyopherin-*α* (Bimax2). NLS from the T antigen of SV40 virus (NLS^{SV40}) fused with EGFP was used as a positive control. Expression of TagRFP-Bimax2 leads to a decrease in the nuclear accumulation of NLS^{SV40}. A decrease in nuclear accumulation was also detected for five prokaryotic proteins, namely, PriA, Lig, PolB, SigA1 and Dcm.

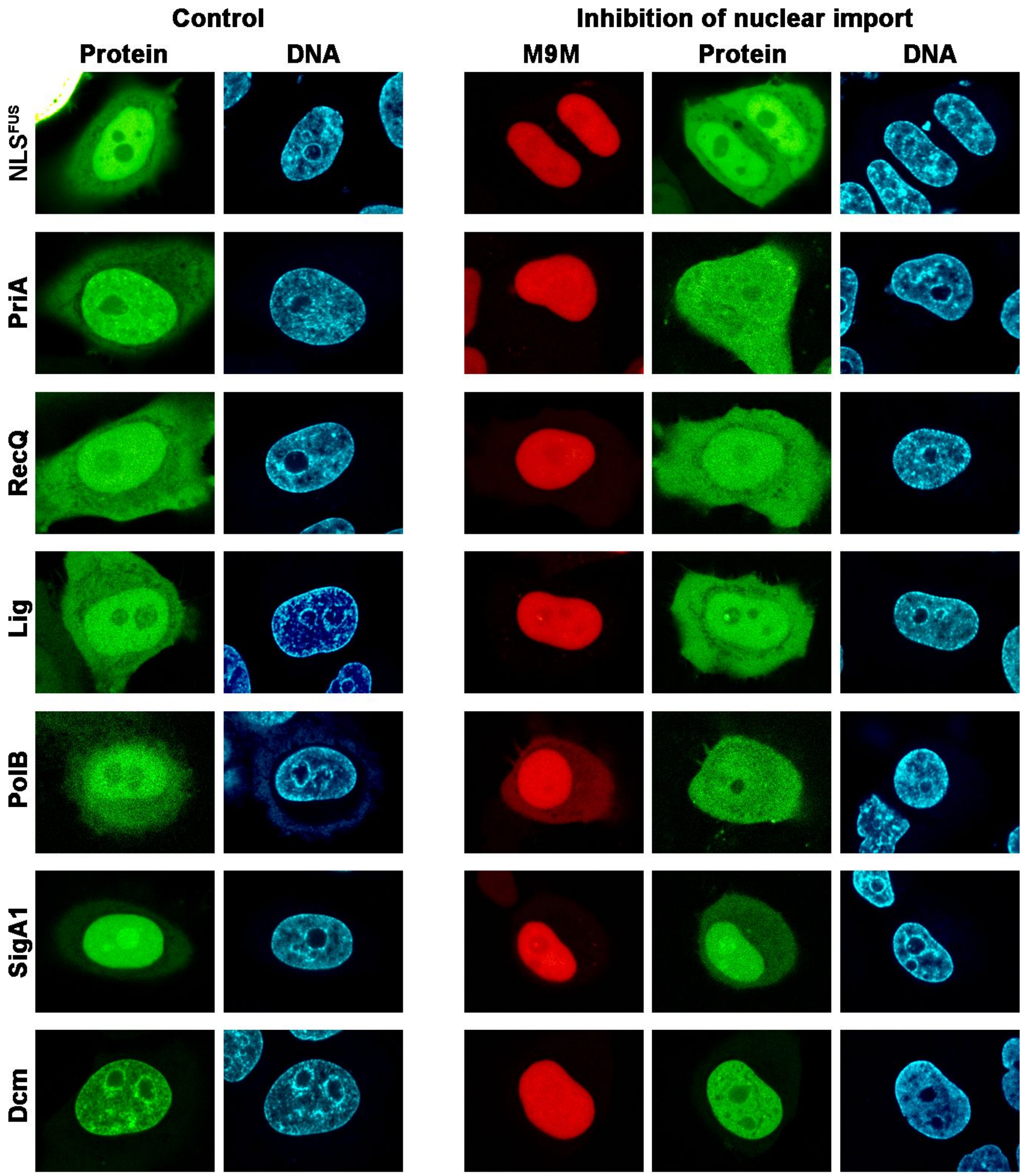

Supplementary Fig. S3. Decrease in the nuclear accumulation of prokaryotic proteins by a peptide inhibitor of karyopherin-*β2* (M9M). The NLS from FUS protein (NLS^{FUS}) fused with EGFP was used as a positive control. The expression of TagRFP-M9M leads to a decrease in the nuclear accumulation of NLS^{FUS}. A decrease in nuclear accumulation was also detected for five prokaryotic proteins, namely, PriA, RecQ, Lig, PolB and SigA1.
